# Single-cell profiling identifies obesity-disrupted brain glucose sensing

**DOI:** 10.1038/s41467-026-77116-9

**Published:** 2026-08-31

**Authors:** Jenny M. Brown, Marie A. Bentsen, Anja Moltke Jørgensen, Thomas Svava Nielsen, Dylan M. Belmont-Rausch, Din Selmanovic, Oksana Dmytriyeva, Charlotte Høy Kruse, Nadia N. Aalling, Dana C. Schwalbe, Oliver Knights Møller, John N. Campbell, Anna Secher, Michael W. Schwartz, Tune H. Pers

**Affiliations:** 1 Novo Nordisk Foundation Center for Basic Metabolic Research, University of Copenhagen, Copenhagen, Denmark; 2 Department of Biomedical Informatics, Harvard Medical School, Boston, MA USA; 3 Department of Biology, University of Virginia, Charlottesville, VA USA; 4 Research and Development, Novo Nordisk A/S, Måløv, Denmark; 5 University of Washington Medicine Diabetes Institute, Department of Medicine, University of Washington, Seattle, WA USA

**Keywords:** Type 2 diabetes, Transcriptomics, Transcriptomics

## Abstract

Neuronal nutrient sensing in the hypothalamus and brainstem is thought to regulate energy and glucose homeostasis, yet how these brain regions respond to elevated adiposity or blood glucose remains unclear. We performed single-nucleus RNA-sequencing on 75,787 cells from the arcuate nucleus, ventromedial hypothalamus, and dorsal vagal complex of control diet- and high-fat diet (HFD)-fed male mice following intravenous glucose or saline infusion. HFD broadly altered gene expression across cell types, including Th/Slc6a3, Agrp, Ghrh, and Trh/Cxcl12 neurons. In control diet-fed mice, hyperglycemia triggered transcriptional responses conserved across brain regions in astrocytes and oligodendrocytes, and a response in *Ghrh* neurons, consistent with reduced neuronal activity. The transcriptional responses to hyperglycemia were largely absent or reversed after HFD. Genes enriched in both mouse and human *GHRH* neurons were located in loci genetically linked to human glycemic traits. These findings point to the brain’s intrinsic capacity to sense hyperglycemia and suggest that this detection system is profoundly impaired in obesity.

## Abstract (web summary)

How obesity disrupts central glucose sensing remains poorly understood. Here, the authors use single-cell transcriptomics to identify hypothalamic and hindbrain neurons that respond to elevated blood glucose and show these responses are impaired in obesity.

## Introduction

Obesity and type 2 diabetes (T2D) are closely linked metabolic disorders characterized by the defended elevation of key metabolic variables (levels of stored and circulating fuel, respectively)^1^. Mounting evidence implicates dysfunction of neurocircuits controlling fuel homeostasis in the pathogenesis of these disorders^2–5^. While the brain clearly plays a key role in the adaptive response to hypoglycemia^6–8^, and while evidence from ex vivo slice electrophysiology^9^ and in vivo recordings indicates that neuronal activity can be affected by glucose levels^10–13^, the question of how the brain detects and responds to hyperglycemia remains poorly understood. Glucose-responsive neurons are concentrated in hypothalamic and hindbrain regions involved in energy homeostasis, including the arcuate nucleus/ventromedial hypothalamus (ARC/VMH) and dorsal vagal complex (DVC) of the caudal hindbrain^14–16^. These regions regulate food intake and energy expenditure as well as glucose metabolism, and in preclinical models, dysfunction localized to these brain areas can promote obesity as well as hyperglycemia^1^. Clarifying how neural circuits in these key brain regions respond to hyperglycemia and how these responses are impacted by obesity has the potential to shed new light on neuronal mechanisms linking obesity to impaired glucose homeostasis.

Commonly used models of obesity are induced by switching animals from standard chow or a purified low-fat diet onto a high-fat diet (HFD), and this diet switch can have untoward metabolic effects independently of obesity per se^17^. Thus, we sought in the current work to focus more on exposure to HFD than on obesity per se in assessing how critical brain cell populations respond to hyperglycemia. To this end, we performed single-nuclei RNA sequencing (snRNA-seq) on both the ARC/VMH and DVC obtained from control diet- and HFD-fed mice following intravenous infusion of either saline or glucose to maintain hyperglycemia for one hour. Compared to saline, hyperglycemia induced robust transcriptional changes across both brain regions in control diet-fed mice. Among glial cells, astrocytes and oligodendrocytes were most responsive, and these responses were conserved across the two brain regions. Among neurons, growth hormone-releasing hormone (*Ghrh*) and agouti-related peptide (*Agrp*)-expressing populations in the ARC exhibited the strongest responses to hyperglycemia, each exhibiting 40 differentially expressed genes enriched in pathways related to synaptic transmission and neurotransmitter release.

Overall, these responses were suggestive of dampened activity of both neuronal populations, offering direct evidence of how neurons in key areas of the brain detect and respond to an elevated blood glucose level. Strikingly, each of these cellular responses to hyperglycemia was either suppressed or inverted in mice fed an HFD. For example, *Ghrh* neurons showed transcriptomic signatures consistent with neuronal inhibition by hyperglycemia in control diet-fed mice, and this effect was lost in HFD-fed mice. Notably, human genetic variants associated with both fasting and random glucose levels were enriched near genes expressed in mouse and human *GHRH* neurons, underscoring the potential translational relevance of these findings.

Our results suggest that in control diet-fed mice, *Ghrh*- and *Agrp*-expressing neurons in the ARC detect and respond to an elevated blood glucose level, and that consuming HFD profoundly impairs this neuronal response. These findings raise the possibility of a previously less well-recognized role of *Ghrh* neurons in fuel homeostasis, and they extend published evidence that HFD feeding blunts the acute inhibitory effect on *Agrp* neuron activity of various nutritional cues^18^. These findings collectively justify further investigation into the relationship between neuronal hyperglycemia detection and obesity-associated glucose metabolic impairment.

## Results

### A comprehensive map of transcriptional responses to nutritional perturbations across hypothalamus and hindbrain cell populations

**Fig. 1:**
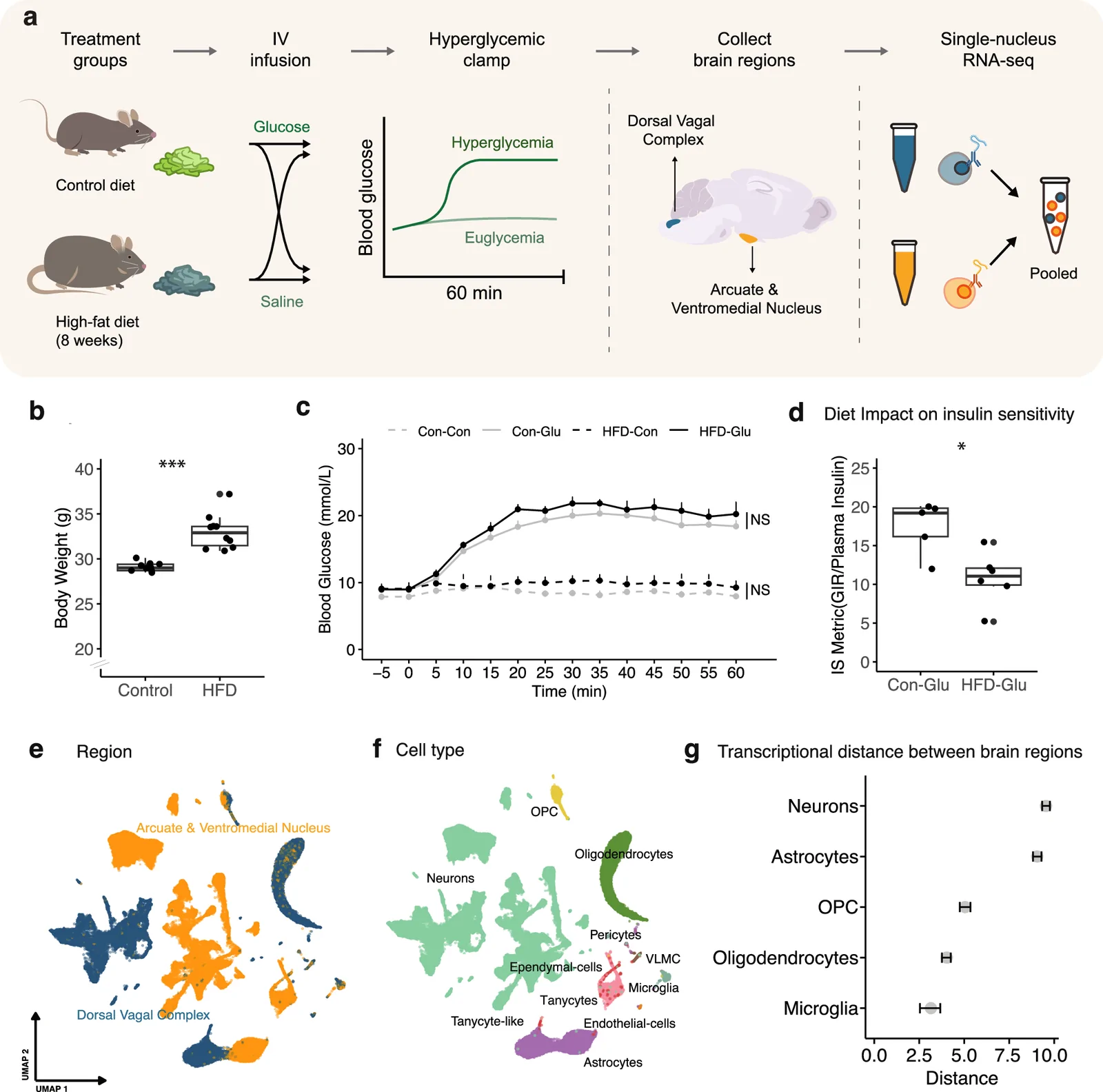
Experimental setup to study glucoregulatory gene expression in the hypothalamus and hindbrain at single-cell resolution. **a** An overview of the in vivo study design. **b** Body weight of control (*n* = 9) and high-fat diet (HFD) fed mice (*n* = 11) after 8-weeks (two-sided t-test t = 12.53, df = 28.4, *P* = 4.24 × 10^−13^). **c** Blood glucose levels during clamp procedure across four treatment groups data are presented as mean values ± SEM: Control diet + control infusion (Con-Con) *n* = 4, Control diet + glucose infusion (Con-Glu) *n* = 5, HFD + control infusion (HFD-Con) *n* = 5, and HFD + glucose infusion (HFD-Glu) *n* = 6. No statistical difference Con-Con:HFD-Con or Con-Glu:HFD-Glu (repeated measures nparLD ANOVA test statistic = 1.68, df = 1, *P* = 0.19, and Statistic = 0.26, df = 1, *P* = 0.089). **d** Insulin sensitivity (IS) (glucose infusion rate (GIR) / plasma insulin during the steady state) in Con-Glu (*n* = 5) versus HFD-Glu (*n* = 6) groups (two-sided t-test t = 4.26, df = 2.97, *P* = 0.024). **e**,** f** Uniform manifold approximation and projection (UMAP) visualization of single-nucleus data colored by brain region and cell types (VLMC vascular and leptomeningeal cells; OPC oligodendrocyte progenitor cells). **g** Transcriptional distance between ARC/VMH and DVC cell populations represented by the point. Error bars indicate the 95% confidence interval of the estimated distance (*n* = 20). Box plots show the median, first and third quartiles, and whiskers extend to 1.5× the interquartile range. Individual points represent animals.

Our studies had three primary objectives: *1)* determine the impact of HFD feeding on cell populations in the ARC/VMH and DVC, *2)* identify cell populations that are responsive to hyperglycemia in control diet-fed mice, and *3)* test whether HFD feeding alters these normal responses. To this end, we employed a modified hyperglycemic clamp protocol in freely moving adult male C57BL6J mice after they had been fed either a control diet matched for sucrose and fiber or an HFD for eight weeks to induce modest weight gain (Fig. 1a, b and Supplementary Fig. 1a). Thus, four groups were studied: *i)* control diet-fed mice receiving intravenous saline, *ii)* control diet-fed mice receiving intravenous glucose, *iii)* HFD-fed mice receiving intravenous saline, and *iv)* HFD-fed mice receiving intravenous glucose. By design, the glucose infusion protocol raised the blood glucose level to 20 mmol/L by the 30-min time point and was clamped at that value for another 30 min (Fig. 1c and Supplementary Fig. 1b). Consistent with previous reports^19^, HFD was associated with elevated plasma insulin levels (Supplementary Fig. 1c) and reduced insulin sensitivity (Fig. 1d).

To eliminate the confounding effect of variation in food intake immediately prior to the study, food was removed at the beginning of the light cycle, 4 h before initiating intravenous infusion. At the conclusion of the 60-min glucose infusion protocol, animals were euthanized, and samples from ARC/VMH and DVC were collected from all mice. Nuclei were isolated from these regions and processed for snRNA-seq. To assess transcriptional responses while minimizing technical batch effects, we used oligonucleotide-based barcodes to tag cells from a given sample, pooled all cells, and sequenced the ARC/VMH and DVC samples from the four treatment groups together across three sequencing runs (Supplementary Fig. 2a). These barcodes enable the identification of both the sample and the originating brain region for each captured nucleus. Canonical markers were used to classify cell populations across broad classes, and quality control was assessed separately on neurons and non-neuronal cells. Following quality control analyses, a total of 47,703 neurons and 28,084 non-neuronal cells with a median number of 3548 and 9972 transcripts and a median number of 3992 and 1790 unique genes, respectively, were included in the downstream analysis (Supplementary Fig. 2b).

To further classify neuronal and non-neuronal cell populations, we used cell type-specific marker genes from published hypothalamic and hindbrain single-cell studies (Fig. 1e, f and Supplementary Fig. 2c). While the percentage of each cell population in each treatment group did not differ by group, as expected, the percentage did vary by brain region with for instance oligodendrocytes comprising only 3.06% of the total cells from the ARC/VMH *vs*. 26.68% in the DVC (Fig. 1 and Supplementary Fig. 2d). Using scDist, a tool to quantify transcriptional differences between groups of cells^20^, we also identified significant transcriptional differences between neurons, astrocytes, oligodendrocyte precursor cells, oligodendrocytes, and microglia isolated from the two brain areas of control diet-fed mice that received intravenous saline infusion (Fig. 1g). Based on a total of 75,787 nuclei, these data demonstrate how the transcriptome of a given cell type differs markedly depending on whether it was sampled from the ARC/VMH *vs*. the DVC.

### High-fat diet feeding induces transcriptional changes predominantly in ARC cell populations

**Fig. 2:**
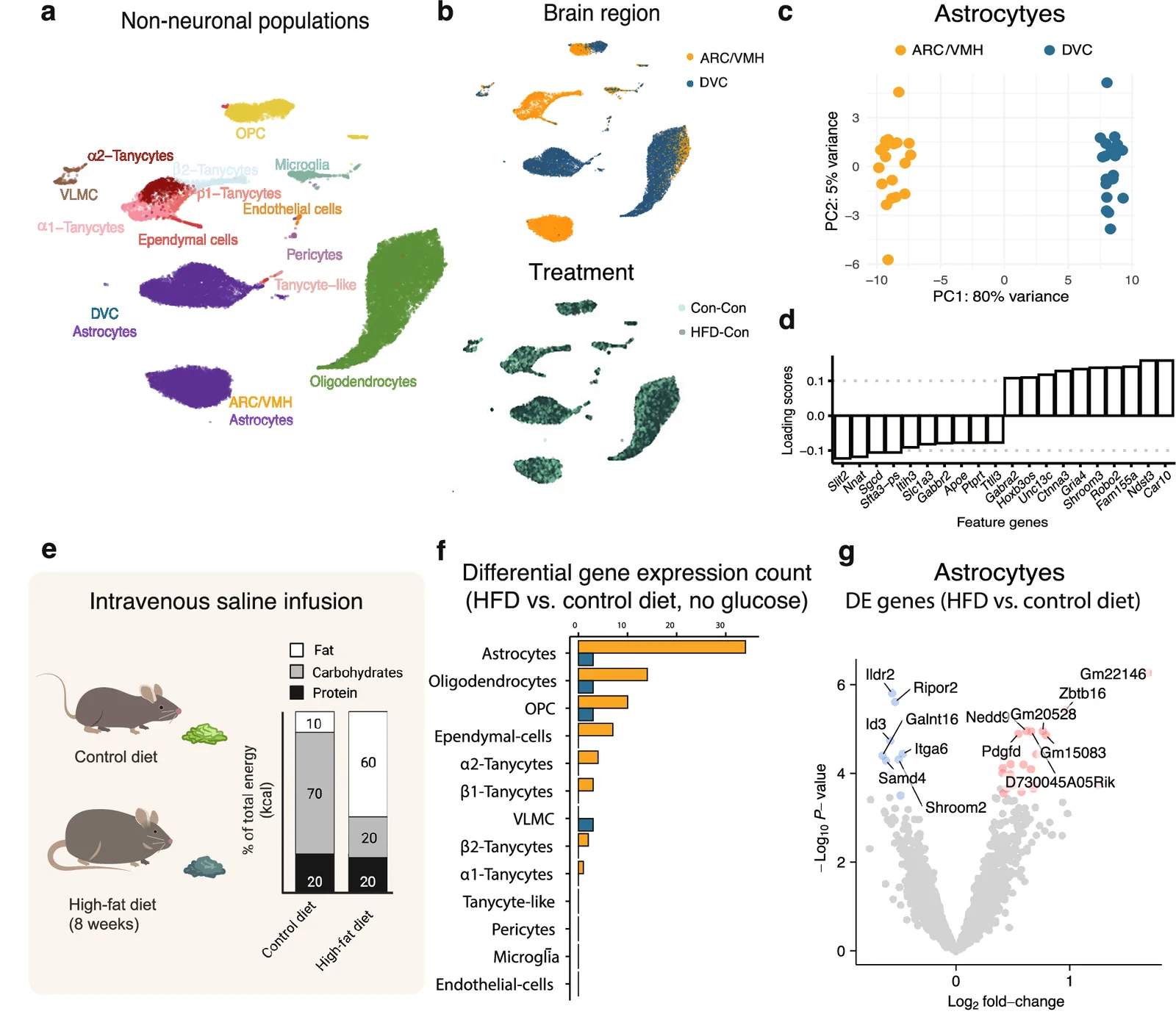
Diet comparison between saline-treated control and HFD-fed mice reveals region-specific transcriptional responses. UMAP plot of non-neuronal cells colored by **a** cell type, **b** brain region (ARC/VMH and DVC), and treatment group (Con-Con and HFD-Con). **c** Principal components (PC) plot showing astrocyte pseudobulk expression patterns separated by the brain region for individual animals. **d** Bar plot showing genes with the highest variation across principal components. **e** Experimental design schematic comparing control diet (Con-Con) and high-fat diet (HFD-Con) mice treated with IV saline. **f** Number of differentially expressed genes identified in each non-neuronal cell population in response to HFD *vs*. control diet-fed mice in the saline infusion group, with ARC/VMH (orange) and DVC (blue) indicated by color. Benjamini-Hochberg -adjusted edgeR likelihood ratio tests FDR < 0.05. **g** Volcano plot showing differentially expressed genes in ARC/VMH astrocytes comparing HFD vs. control diet mice, Benjamini-Hochberg-adjusted edgeR FDR < 0.05.

After eight weeks of HFD feeding, the animals displayed modest weight gain but showed higher plasma insulin levels than chow-fed controls during the glucose infusion (Supplementary Fig. 1c), indicating an early stage of insulin resistance. This physiological profile is well-suited for probing how diet exposure, independent of gross adiposity, affects hypothalamic and hindbrain transcriptional responses to HFD-feeding and acute hyperglycemia. To better label and characterize the glial response to obesity (comparing HFD-fed *vs*. control-fed in saline infused mice), we first identified and re-clustered non-neuronal cells from both brain regions **(**Fig. 2a**)**. Astrocytes from the ARC/VMH and DVC formed separate clusters that did not differ between HFD and control-fed mice (Fig. 2b). To understand how astrocytes from the ARC/VMH differ from the DVC transcriptionally we ran a principal component (PC) analysis and found that PC1 perfectly separated astrocytes by brain regions explaining 80% of the variation across astrocytes (Fig. 2c). Genes with higher expression in ARC/VMH astrocytes *Slc1a3, Gabbr2*, *Apoe, Sgcd*, *Slit2, Ptprt*, and *Itih3*; Fig. 2d). By comparison genes with higher expression in DVC astrocytes were *Gria4, Gabra2*, *Robo2, Ctnna3*, *Unc13c*, *Ndst3*, and *Car10*; Fig. 2d). These observations extend previous findings that astrocytes are not only transcriptionally diverse across forebrain regions but also across the ARC/VMH and DVC^21^.

To investigate how HFD feeding reshapes neuronal and glial states across these two brain regions, we compared the transcriptome from chow- and HFD-fed mice treated with intravenous saline (i.e., in the absence of glucose infusion; Fig. 2e). For each sample in a given brain region, we combined the cells mapping to a given cluster into a “pseudobulk” sample and used the edgeR tool^22^ to identify differentially expressed genes between cells from HFD *vs*. control diet-fed mice. Genes with a Benjamini–Hochberg (BH)-adjusted false-discovery rate (FDR) *P*-value < 0.05 were considered differentially expressed (throughout all analyses reported in this manuscript). In response to HFD, we detected a total of 75 differentially expressed genes across all non-neuronal cell populations in the ARC/VMH and 12 in the DVC. Among glial cells, astrocytes, oligodendrocytes, and oligodendrocyte precursor cells exhibited the greatest number of differentially expressed genes (Fig. 2f, Supplementary Data 1 and 2). These findings add to growing evidence that astrocytes exhibit region-specific transcriptional profiles, highlighting functional specialization across brain areas.

Consistent with previous studies, we confirmed that in the ARC/VMH, HFD feeding was associated with increased GFAP protein content compared to control diet^23^. However, this effect was not observed in the DVC (Supplementary Fig. 3a). This was also reflected in the difference in differentially expressed genes that were detected in the ARC/VMH astrocytes compared to the DVC. Pathway analysis of differentially expressed genes in ARC/VMH astrocytes from HFD-fed vs. chow-fed mice (Fig. 2g) suggested down-regulation in cell-to-cell communication and tight junctions (Supplementary Fig. 3b). They also showed that relative to the substantial variability driven by cell identity and anatomical location, the transcriptional state of astrocytes was only modestly impacted after 8 weeks of HFD feeding. These findings suggest that the DVC, relative to the ARC/VMH, to a lesser extent, is subject to transcriptional changes following HFD.

**Fig. 3:**
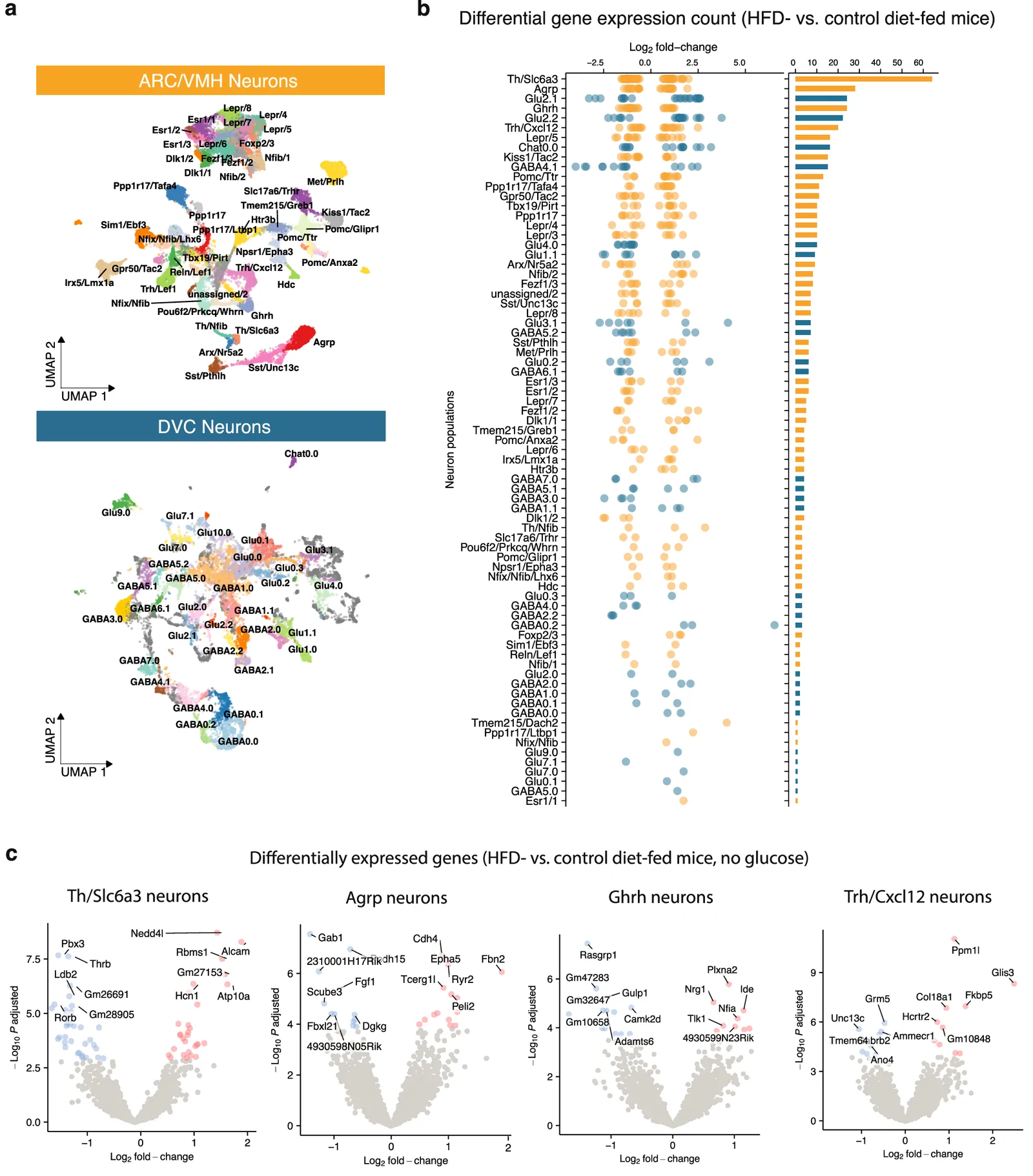
Diet comparison between saline-treated control and HFD-fed mice reveals region-specific transcriptional responses. **a** UMAP plot of ARC/VMH and DVC neuron populations colored by cell type. **b** Number of differentially expressed genes (FDR < 0.05) in neuronal populations comparing HFD *vs*. control diet-fed mice in the saline infusion group, with ARC/VMH (orange) and DVC (blue) indicated by color. **c** Volcano plots showing differentially expressed (DE) genes in HFD *vs*. control diet-fed across the top four ARC neuronal populations. Differentially expressed genes Benjamini-Hochberg -adjusted edgeR likelihood ratio tests FDR < 0.05 are colored in red (upregulated in HFD) and blue (downregulated).

To investigate transcriptional responses to HFD feeding across neurons, we first used label transfer from three publicly available datasets to classify neurons into previously defined neuron populations from the ARC/VMH and DVC (cell populations with less than 25 cells from each treatment group were excluded from further analysis). This analysis identified a total of 31,126 and 16,577 neurons retained across the 48 hypothalamic neuronal ARC/VMH and 32 DVC cell types, respectively (Fig. 3a and Supplementary Data 3). In the ARC/VMH of mice receiving intravenous saline, transcriptional responses to HFD-feeding were observed predominantly in three GABAergic cell populations (co-expressing *Slc32a1*), namely *Agrp, Ghrh*, and *Trh/Cxcl12* neurons and one dopaminergic population, namely *Th/Slc6a3* (Fig. 3b, c); and in DVC neurons in two glutamatergic populations, Glu2.1 and Glu2.2, a cholinergic Chat0.0 and GABAergic GABA4.1 (Fig. 3b and Supplementary Fig. 4a, b). Two of these populations (*Th/Slc6a3* and *Agrp* neurons) have previously been shown to be transcriptionally regulated by HFD feeding and fasting^24–26^. As *Ghrh* neurons are reported to project onto *Agrp* neurons^27^ in this context, it is notable that *Agrp* and *Ghrh* neurons have also been linked to obesity and T2D in non-human primates^27,28^. By comparison, *Trh/Cxcl12* neurons (also referred to as *Bnc2* and *Lepr/Glp1r* neurons in the literature) are implicated in energy homeostasis and taste perception^29,30^. Based on spatial transcriptomics data from a recent DVC-focused study^31^, Glu2.1 and Glu2.2 are located in the nucleus of the solitary tract, while Chat0.0 is located in the dorsal motor nucleus of the vagus, a part of the DVC that has previously been linked to changes in food intake and blood glucose^32,33^. Together, these results show that HFD feeding transcriptionally impacts several key neuronal populations involved in energy homeostasis in the ARC/VMH and DVC.

### Conserved responses to hyperglycemia in astrocytes across brain regions

**Fig. 4:**
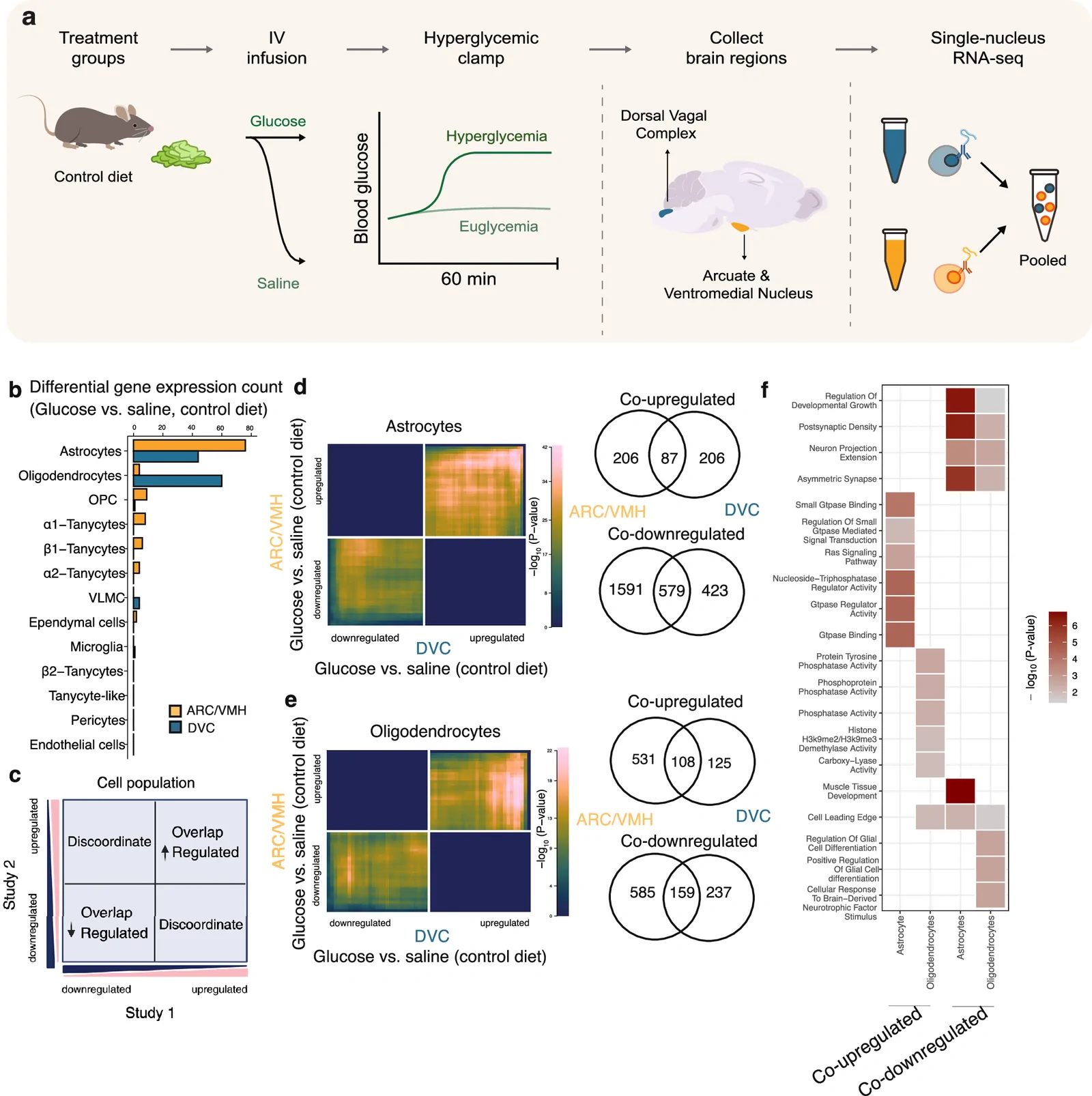
Conserved glial responses to hyperglycemia across brain regions in lean control diet-fed mice. **a** Experimental design schematic showing glucose or saline infusion protocol in lean mice maintained on a control diet. **b** Quantification of differentially expressed genes (FDR < 0.05) across cell populations, comparing glucose versus saline infusion conditions in control diet-fed mice. **c** Schematic explaining interpretation of rank-rank hypergeometric overlap analysis, where intense pink regions (“hotspots”) indicate strongly coordinated or discordant transcriptional responses between experimental conditions. **d** Rank-rank hypergeometric overlap analysis of all expressed genes between ARC/VMH and DVC astrocytes, shown as a heatmap, with accompanying Venn diagram depicting shared upregulated and downregulated genes. **e** Same analysis as in (**d**) but for oligodendrocytes from ARC/VMH and DVC regions. **f** Most significantly enriched GO-terms identified from co-upregulated and co-downregulated genes in astrocytes and oligodendrocytes, based on overlap analyses shown in (**d**, **e**), performed using g:Profiler (gost) with a cell type-specific background gene set. Statistical significance was determined using hypergeometric tests with Benjamini–Hochberg-adjusted FDR < 0.05.

Having defined the effects of HFD feeding under baseline (saline) conditions, we next examined how the brain responds to acute hyperglycemia in control diet-fed mice, to identify glucose-responsive cell populations and establish a reference point for evaluating HFD-induced changes (Fig. 4a). Focusing on by-region responses to hyperglycemia among glial cells, astrocytes exhibited the strongest response to hyperglycemia in control diet-fed mice, with 76 differentially expressed genes in the ARC/VMH and 44 genes in the DVC (Fig. 4b). Despite the fundamentally different transcriptome of astrocytes in these two brain areas, there was substantial overlap in the effect of hyperglycemia on astrocyte gene expression between the two brain regions (Supplementary Fig. 5a). These results were confirmed by a rank-rank hypergeometric overlap analysis, a statistical approach that quantifies the significance of shared gene expression changes between ranked gene lists, thereby identifying conserved or divergent transcriptional responses for all genes across regions (Fig. 4c, d). Gene Ontology analysis of these co-upregulated genes revealed enrichment for pathways related to small GTPase binding, regulation of small GTPase-mediated signal transduction, and Ras signaling (Fig. 4f). Among co-downregulated genes, pathways were associated with post-synaptic density and asymmetric synapses. These findings suggest that *i)* in normal mice, astrocytes in both hypothalamus and hindbrain can detect and respond to a sustained increase of blood sugar; and *ii)* that the astrocytic response involves genes implicated in intracellular signaling, cytoskeletal dynamics, and synapse biology.

In the DVC, oligodendrocytes exhibited the strongest response to hyperglycemia in control diet-fed mice, exhibiting 61 differentially expressed genes (Fig. 4b). Although fewer differentially expressed genes were detected in the ARC/VMH oligodendrocytes, we used the rank-rank hypergeometric overlap analysis to compare the rank fold changes of all genes. Interestingly, oligodendrocytes exhibited a similar coordinated response in ARC/VMH (Fig. 4e) with 108 overlapping upregulated and 159 downregulated. Co-upregulated genes in ARC/VMH and DVC oligodendrocytes were associated with protein tyrosine and phosphoprotein phosphatases activity (Fig. 4e, f). Among co-downregulated three of the top 10 pathways were shared between oligodendrocytes and astrocytes across the two brain areas (Fig. 4f). These include processes related to postsynaptic density, neuron projection extension, and asymmetric synapse formation, suggestive of a conserved reduction in synaptic structural components in response to hyperglycemia. The finding that in control diet-fed mice, oligodendrocytes in the DVC exhibit the strongest transcriptional response to hyperglycemia indicates that glial activation may partially drive neuronal responses under healthy conditions. Further studies are needed to determine whether these changes reflect physiological oligodendrocyte adaptations.

In addition to increasing brain exposure to glucose, hyperglycemia also increased plasma insulin levels (Supplementary Fig. 1c). We considered that in insulin receptor–expressing cell types, transcriptional effects of glucose infusion might reflect insulin signaling. Since astrocytes show variable insulin receptor expression (Supplementary Fig. 5b), we examined whether this influenced their response to hyperglycemia in the ARC/VMH and DVC. Upon stratifying astrocytes based on insulin receptor expression, we found that the astrocyte transcriptional response to hyperglycemia did not differ based on insulin receptor status in either of these regions (Supplementary Fig. 5c, d). We therefore infer that conserved astrocytic responses to hyperglycemia across hypothalamic and DVC populations in the present study are less likely to be driven by increased insulin action.

### Hyperglycemia inhibits activity-associated cellular responses in both *Ghrh* and *Agrp* neurons

**Fig. 5:**
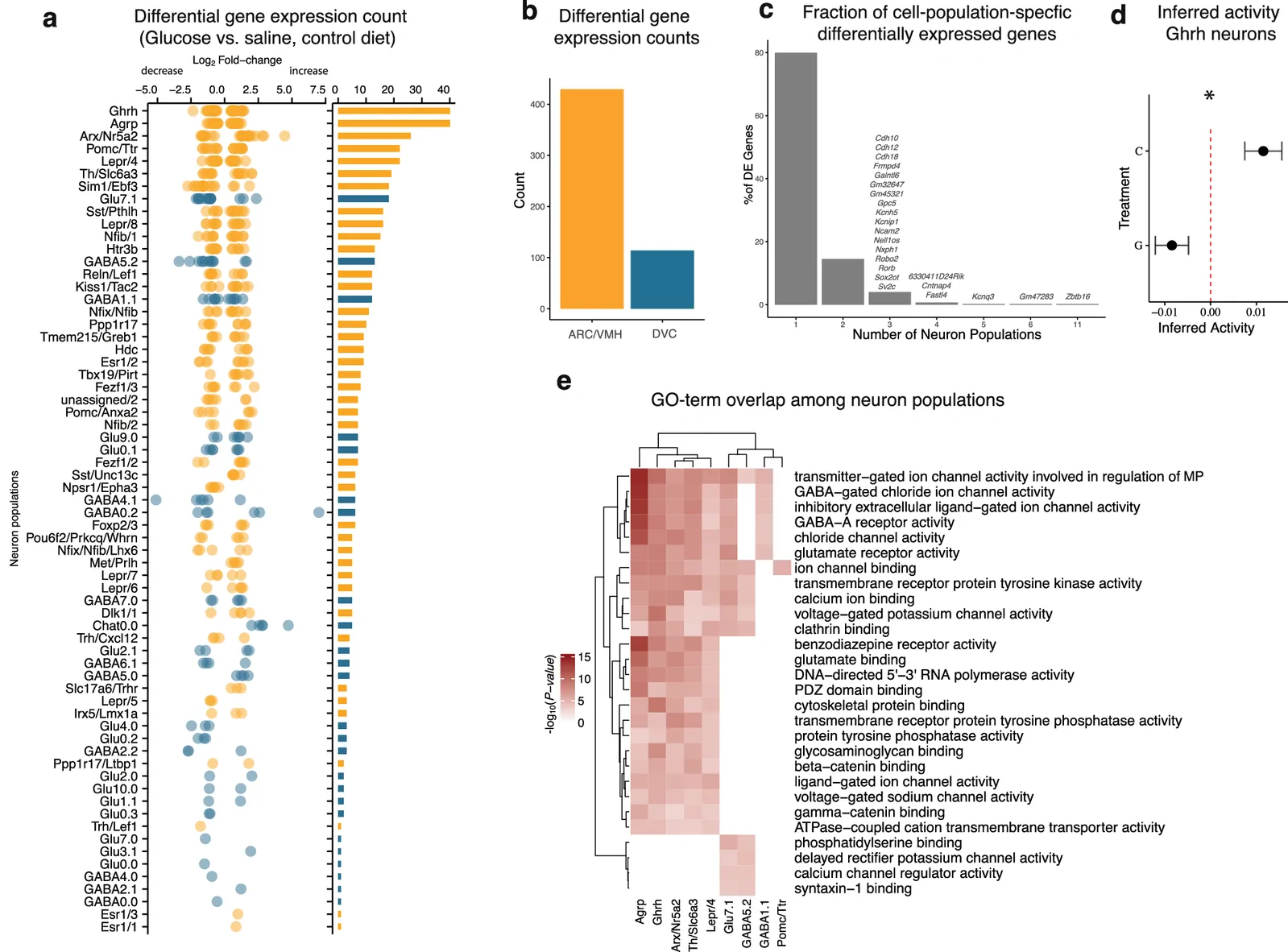
Glucose-induced transcriptional effects in neurons identified with single-nucleus transcriptomics across the ARC/VMH and DVC. **a** Number of differentially expressed genes (FDR < 0.05) comparing glucose vs. saline infusion in control diet-fed mice across neuronal populations with ARC/VMH (orange) and DVC (blue) indicated by color. **b** Number of differentially expressed genes comparing ARC/VMH (orange) and DVC (blue) brain regions in total. **c** Percentage of differentially expressed genes that are unique to single neuronal populations vs. shared across multiple populations. **d** Estimated marginal mean immediate early genes activity score in Ghrh neurons from saline-infused (C) *n* = 4 and glucose-infused (G) *n* = 5, and error bars represent ± SE (*, FDR < 0.05). **e** Heatmap showing overlap of molecular function GO terms in response to glucose across neuronal populations with the highest number of differentially expressed genes from ARC/VMH and DVC, with “MP” indicating postsynaptic membrane potential. Functional enrichment analysis of the input gene list was performed using the MetaBrain Network. Significantly enriched terms are shown (Benjamini–Hochberg-adjusted FDR < 0.05).

To investigate cellular responses to hyperglycemia across neurons in ARC/VMH and DVC of control diet-fed control mice, we first compared the total transcriptional response to intravenous glucose *vs*. saline infusion across both brain regions. The most pronounced differential gene expression responses to hyperglycemia were in ARC/VMH neurons (430 vs. 130; Fig. 5a, b and Supplementary Data 4, 5), with *Ghrh* and *Agrp* neurons responding the most. Among the differentially expressed genes identified in *Ghrh* neurons, *Foxp2* (Supplementary Fig. 6a), a marker of tuberal VMH neurons^34^, was detected in approximately half of *Ghrh*-positive cells (50.8%) in wild-type mice by RNAscope (Supplementary Fig. 6b, c), confirming its expression in *Ghrh* neurons in situ.

Among DVC neuron populations, Glu7.1, GABA5.2 and GABA1.1 cells were the strongest responders to hyperglycemia. Based on a recent spatial transcriptomics dataset from rat DVC^31^, they maybe localized to a hindbrain area dorsal to the DVC.

Using the expression of immediate-early genes as a proxy, we next asked whether *Ghrh* neuron activity is likely to be impacted by hyperglycemia. We found that expression of immediate-early genes was reduced by hyperglycemia, specifically in *Ghrh*-expressing cells, suggesting that the activity of these neurons was reduced following hyperglycemia (Fig. 5d and Supplementary Fig. 7a–c). We also investigated whether neurons exhibiting a significant transcriptional response to hyperglycemia also express canonical glucose-sensing machinery (e.g., *Gck*, *Slc2a2*, *Abcc8*, and *Kcnj11)*, which would be an indication of their direct glucose-sensing capability. Because these genes are lowly expressed and hence hard to detect in snRNA-seq data, we used adaptively-thresholded low-rank approximation^35^ to impute missing values in snRNA-seq data across all ARC/VMH neuron populations. Based on the imputed expression of these genes, we found that *Ghrh*-expressing neurons were among the top five ARC/VMH populations with the highest expression of these genes combined (Supplementary Fig. 8a). To determine whether glucose-responsive neuronal populations express core glucose-sensing machinery in situ, we performed RNAscope and found that the majority of both *Ghrh* and *Th/Slc6a3*-positive neurons co-expressed *Gck* (Ghrh⁺: 88.2%; *Th/*Slc6a3⁺: 84.1%; Supplementary Fig. 8b–d). This observation is consistent with findings that glucose-sensing properties intrinsic to *Ghrh*-expressing neurons may enable them to detect and respond to hyperglycemia^36^.

To determine the extent to which the neuronal responses to hyperglycemia were cell-population-specific *vs*. shared between neuron populations, we first assessed the amount of differentially expressed genes in one or more cell populations. Across the ARC/VMH and DVC, 80% of hyperglycemia-induced differentially expressed genes were unique to one cell population (Fig. 5c). To assess whether the differentially expressed genes are likely to act within the same molecular pathways, we used a brain-specific gene set enrichment tool, MetaBrain^37^, focusing on the most responsive cell populations (>10 differentially expressed genes) from the two brain regions (Supplementary Data 4, 5). A substantial number of gene set terms were enriched in at least five out of 10 cell populations, including transmitter-gated ion channel activity regulating postsynaptic membrane potential, GABA-gated chloride ion channel activity, GABA-A receptor activity, chloride channel activity, and glutamate receptor activity (Fig. 5d and Supplementary Data 6).

These collective findings suggest that broadly speaking, the neuronal response to hyperglycemia was linked to changes in GABAergic signaling and inhibitory neurotransmission. CellChat^38^ and Weighted Kolmogorov Smirnov testing analyses suggested that GABA-A receptor activity is upregulated in *Agrp* cells (Supplementary Fig. 9a–c), suggesting that in control diet-fed mice, hyperglycemia reduced their activity. This finding is consistent with evidence that *Agrp* neuron activity (measured in vivo by fiber photometry) is inhibited by cues relevant to nutritional status^18^. In contrast, the downregulation of this pathway in *Ghrh* neurons (Supplementary Fig. 9a, d, e) could reflect a recovery phase following an initial increase of inhibitory tone, consistent with our observation of decreased immediate-early gene expression following the glucose clamp.

### High-fat diet feeding attenuates the oligodendrocyte response in the DVC and alters glucose sensing in hypothalamic tanycytes and astrocytes

**Fig. 6:**
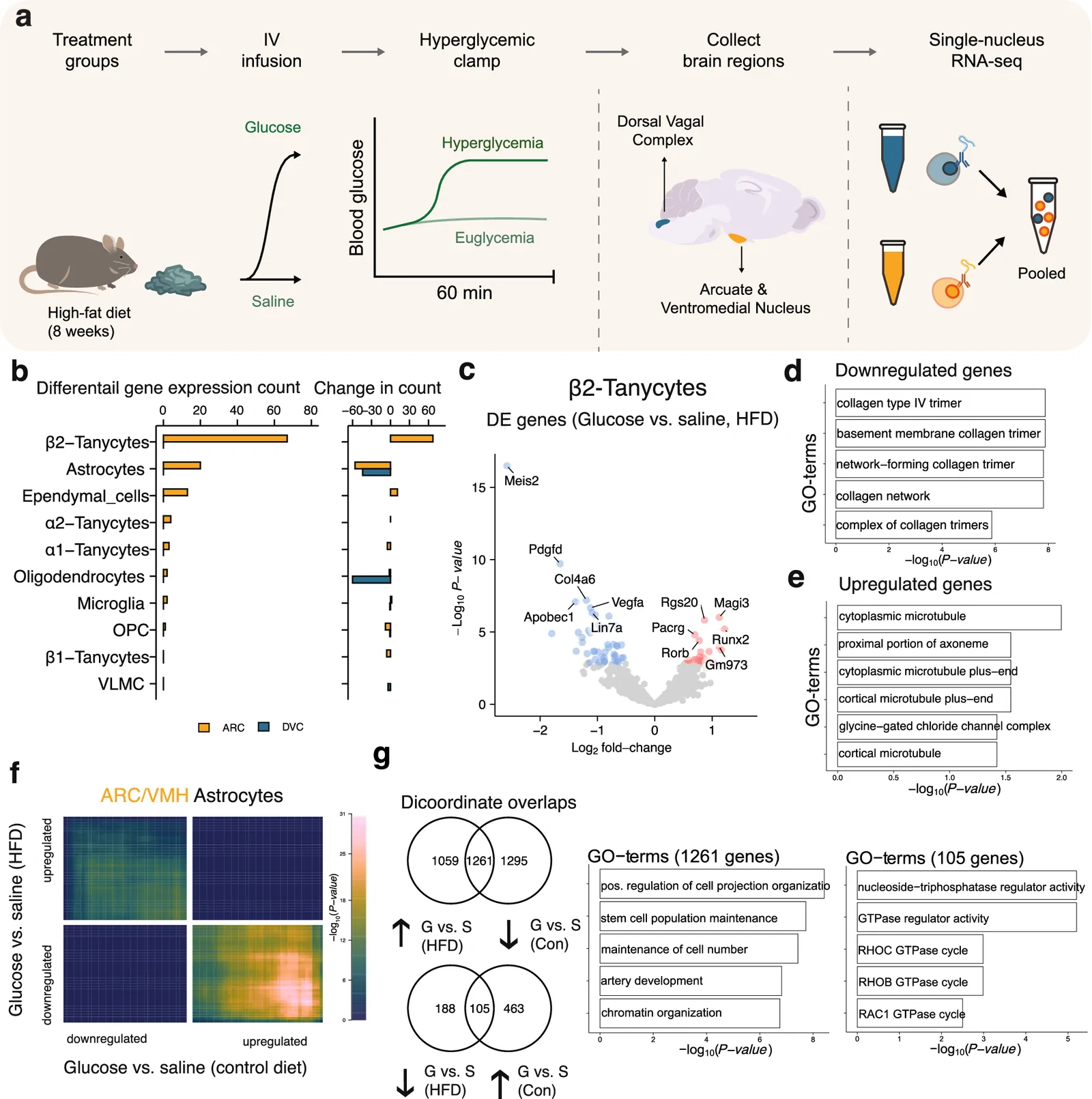
High-fat diet suppresses glucose sensing in non-neuronal cells in the DVC and alters glucose sensing in β-tanycytes and astrocytes in the ARC/VMH. **a** Overview of the in vivo infusion study design in mice fed HFD. **b** Bar plot showing differentially expressed genes (FDR < 0.05) comparing glucose vs. control infusion from HFD-fed mice in each non-neuronal population and the change in differentially expressed genes between glucose responses in control vs. high-fat diet fed mice, with ARC/VMH (orange) and DVC (blue) indicated by color. **c** Volcano plot of differentially expressed (DE) genes in β2-tanycytes. Benjamini-Hochberg -adjusted edgeR likelihood ratio tests FDR < 0.05. **d** The top significant GO terms in β2-tanycytes based on downregulated genes in response to glucose in HFD-fed mice. **e** The top significant GO terms in β2-tanycytes based on upregulated genes in response to glucose in HFD-fed mice. **f** Heatmap of overlapping ranked glucose-responsive genes between the high-fat diet and the control diet in astrocytes by rank-rank hypergeometric overlap analysis. **g** A Venn diagram of discordant overlapping genes and the top significant GO terms in astrocytes from the overlap analysis of the discordant genes from f (G, glucose; S, saline). GO terms determined using g:Profiler (gost) with a cell type-specific background gene set. Statistical significance was determined using hypergeometric tests with Benjamini–Hochberg-adjusted FDR < 0.05.

**Fig. 7:**
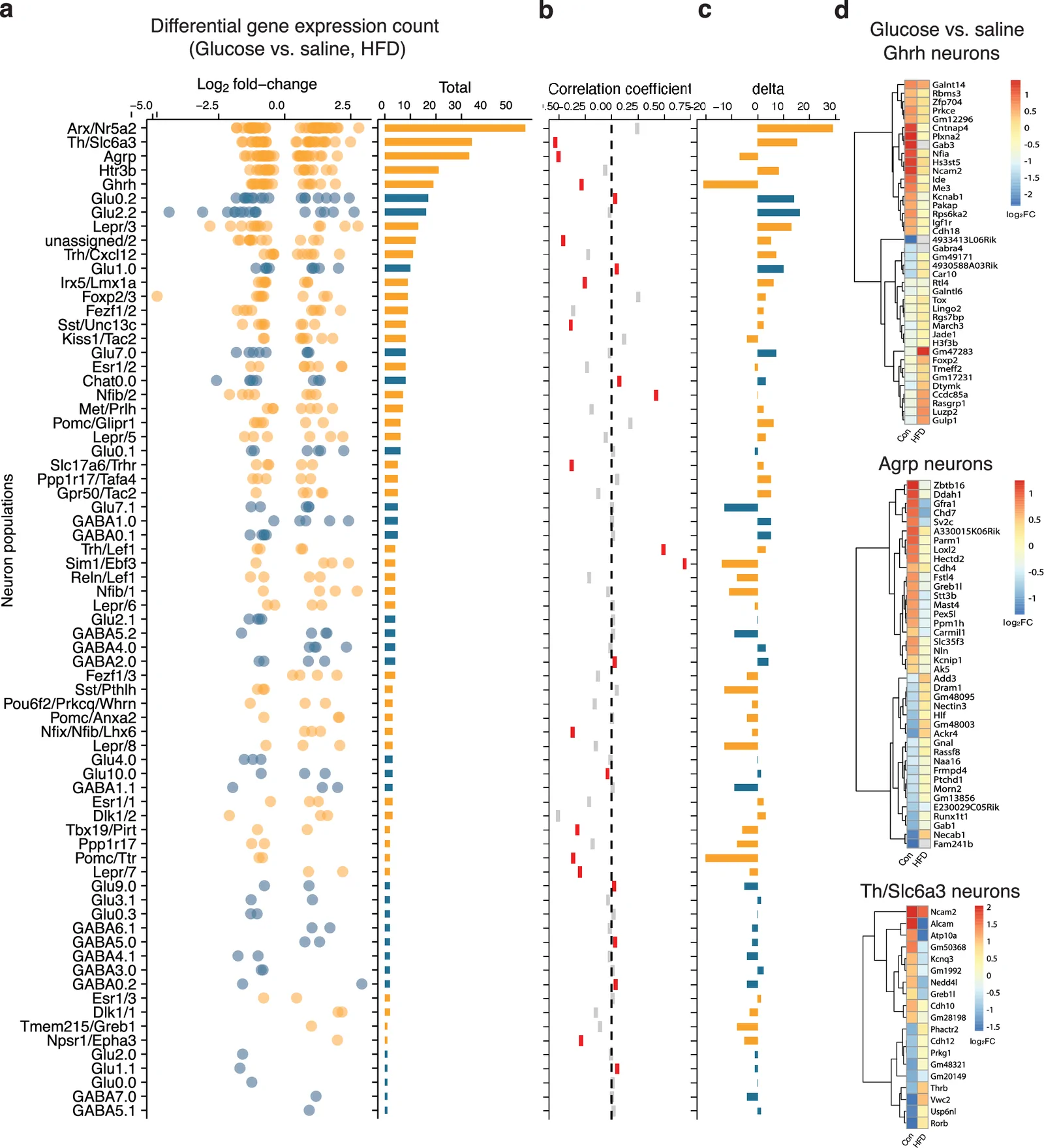
High-fat diet feeding alters glucose sensing in neurons in the ARC/VMH and DVC. **a** Number of differentially expressed genes (FDR < 0.05) comparing glucose vs. saline infusion in HFD-fed mice across neuronal populations with ARC/VMH (orange) and DVC (blue) indicated by color. **b** Kendall correlation analysis of glucose-induced transcriptional responses between control diet and HFD-fed mice. **c** Change in number of differentially expressed genes in response to glucose, comparing control diet vs. HFD-fed mice with ARC/VMH (orange) and DVC (blue), indicated by color. **d** Heatmaps showing differentially expressed genes Benjamini-Hochberg -adjusted edgeR likelihood ratio tests FDR < 0.05 log_2_ fold change (FC) in response to glucose, comparing control diet vs. high-fat diet conditions in Ghrh, Agrp, and Th/Slc6a3 neurons, respectively.

Across both brain regions, we observed striking effects of HFD feeding on glial and neuronal responses to hyperglycemia (Figs. 6a, b and 7a). In control diet-fed mice, astrocytes showed the strongest transcriptional response to hyperglycemia in the ARC/VMH, based on the number of differentially expressed genes (Fig. 3b). By contrast, in HFD-fed mice, β2-tanycytes emerged as the most responsive ARC/VMH cell type (Fig. 6b). Notably, these tanycytes showed no significant transcriptional response to hyperglycemia in control diet-fed mice (Fig. 3b). In HFD-fed mice, hyperglycemia predominantly downregulated genes in β2-tanycytes (Fig. 6c), with enrichment for Gene Ontology terms related to collagen type IV trimer, basement membrane collagen, and the collagen network, suggesting changes affecting the extracellular matrix (Fig. 6d). Conversely, β-tanycytes genes upregulated by hyperglycemia under HFD were associated with cytoplasmic microtubule, proximal portion of axoneme, cytoplasmic microtubule plus-end, cortical microtubule plus-end, glycine-gated chloride channel complex (Fig. 6e). These enrichments suggest cytoskeletal remodeling, tanycytes process extension and altered ciliary or vesicular dynamics, potentially reflecting structural adaptations associated with chronic metabolic stress^39,40^. Notably, previous studies have reported HFD-induced morphological and chemical alterations in β2-tanycytes, including changes in microtubule organization and junctional complexity^39^.

Astrocytes were also strongly impacted by hyperglycemia in the ARC/VMH in control diet-fed mice, but in a manner that differed markedly in HFD-fed mice. Many genes that were upregulated by hyperglycemia in control mice were downregulated in HFD-fed mice, with enrichment for Gene Ontology terms related to GTPase regulator activity (Fig. 6f, g). Small GTPases are known to coordinate endocytic and exocytic vesicle trafficking in astrocytes, including the regulation of membrane transporter localization and recycling^41^. This includes transporters like Glut1, which mediates glucose uptake across the blood-brain barrier^42^. This observation supports a model wherein astrocyte-mediated glucose transport into the brain is disrupted in HFD-fed mice, potentially contributing to dysfunctional neuronal glucose sensing.

Together, these findings indicate that in HFD-fed mice, astrocytes and oligodendrocytes in the DVC fail to mount the normal transcriptional response to hyperglycemia, whereas in the ARC/VMH, β2-tanycytes, which showed no response to hyperglycemia in control diet-fed animals, exhibit numerous differentially expressed genes in HFD-fed mice and the astrocytic response to hyperglycemia in control diet-fed mice was reversed in HFD-fed mice. Additional studies are needed to more fully characterize the functional consequences of HFD-mediated changes in the response to hyperglycemia across glial cell types.

### High-fat diet feeding blunts or reverses neuronal responses to hyperglycemia in both the hypothalamus and the hindbrain

We next determined the effect of HFD on hyperglycemia-induced transcriptional responses in neuronal clusters from the two brain areas. In the DVC, HFD feeding had a generally suppressive effect on transcriptional responses to hyperglycemia, with only two neuronal populations Glu0.2 and Glu2.2 (located in the nucleus of the solitary tract) displaying more than 10 differentially expressed genes (Fig. 7a). We infer from this finding that HFD feeding suppresses hyperglycemia-induced transcriptional activity in most DVC cell types (glial and neuronal) (Figs. 6b and 7a).

A comparison of the response of ARC/VMH neurons to hyperglycemia between HFD and control diet-fed mice revealed pronounced differences (Fig. 7c, d). For example, the effect of hyperglycemia to reduce the activity of *Ghrh* neurons (based on expression of immediate early genes) in control diet-fed mice was absent in HFD-fed mice, suggesting that HFD feeding blocks the change in transcriptional signature consistent with neuronal inhibition effect of hyperglycemia on these neurons (Supplementary Fig. 7a). We found cell-type-specific global transcriptional responses across numerous ARC/VMH populations, *Arx/Nr5a2* and *Th/Slc6a3* neurons being most responsive to hyperglycemia, while *Agrp* and *Ghrh* exhibited a reduced number of differentially expressed genes in response to glucose after HFD-feeding (Fig. 7a, b).

To better visualize the relationship between transcriptional changes induced by hyperglycemia in control diet-fed mice and the impact of HFD feeding on the neuronal response to hyperglycemia, we plotted a heatmap of log-fold changes of the differentially expressed genes in *Ghrh*, *Agrp*, and *Th/Slc6a3* neurons in response to glucose in the control-diet-fed animals compared to the same genes in response to glucose in HFD-fed mice (Fig. 7d**)**. This analysis revealed a generalized reversal of the neuronal responses to hyperglycemia by HFD feeding. We confirmed this inverted pattern in *Ghrh*, *Agrp* and *Th/Slc6a3* neurons by rank-rank correlation analysis (Supplementary Fig. 10a–c). Gene Ontology enrichment analysis of the (inverted) genes showed that in HFD-fed mice, hyperglycemia upregulates pathways related to neuron-to-neuron synapses, postsynaptic specialization, and postsynaptic density across these three ARC/VMH cell populations—pathways that in control diet-fed mice were down-regulated by hyperglycemia. Although not direct evidence of synaptic remodeling, these findings suggest a fundamental shift in how hypothalamic neurons respond to an acute elevation of blood glucose levels in HFD-fed mice.

We observed a positive correlation between the hyperglycemia response in *Th/Slc6a3*, *Agrp*, and *Ghrh* neurons from control diet-fed mice and the effect of HFD feeding in saline-treated mice (Supplementary Fig. 11a). We infer from this finding that exposure to an HFD preconditions neuronal populations to adopt transcriptional states resembling those induced by chronic nutrient excess, thereby blunting a further acute response to hyperglycemia. This suggests that HFD feeding preconditions these neurons into a transcriptional state resembling their chronic adaptation to obesity, thereby blunting their response to acute glucose elevation. More broadly, HFD feeding appears to enforce a metabolically inflexible state that limits neuronal adaptability to changing homeostatic demands. This aligns with previous findings that diet-induced obesity impairs *Agrp* neuron responses to nutritional cues^18^, though whether similar impairments occur in other ARC/VMH populations remains unknown.

### *GHRH* neurons enrich for genetic variants associated with human glycemic traits

**Fig. 8:**
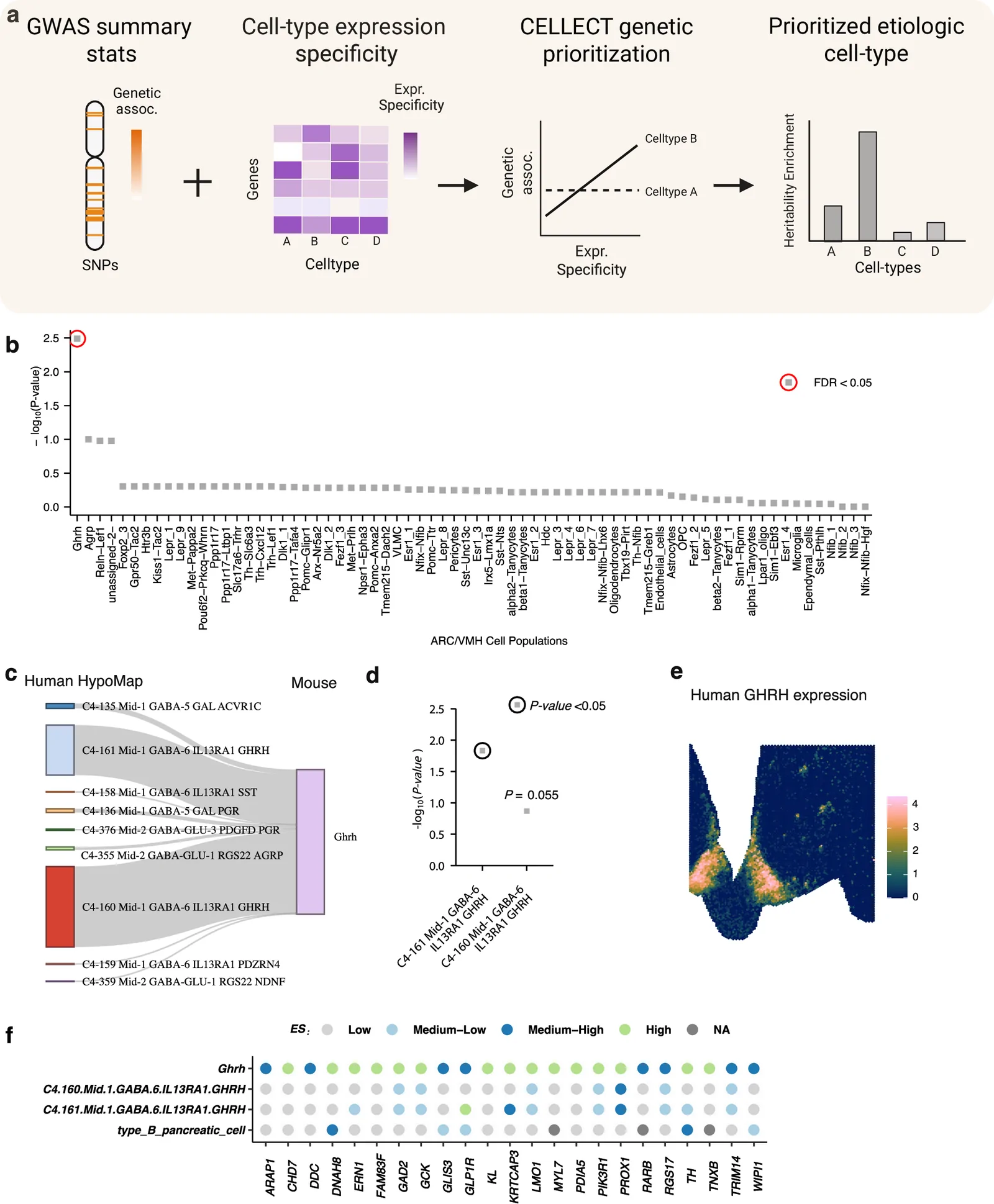
Ghrh neurons show enrichment for glucose-associated genetic variants across species. **a** Schematic of CELLECT analysis workflow integrating single-cell gene expression with genetic prioritization to identify cell populations enriched for glucose GWAS-associated genes. Created in BioRender. Brown, J. (2026) https://BioRender.com/hrxzstn. **b** A Manhattan-style plot of cell type enrichment across hypothalamic populations, identified by performing CELLECT with stratified LD score regression (S-LDSC), significant enrichments Benjamini–Hochberg-adjusted FDR < 0.05 indicated by red circles. **c** Alluvial plot showing transcriptional mapping between human and mouse GHRH-expressing neurons. **d** A Manhattan-style plot showing enrichment of genetic associations in human GHRH neuron populations from HypoMap, identified by performing CELLECT with stratified LD score regression (S-LDSC) with significant enrichments (*P* < 0.05) indicated by black circles (C4-161 *P* = 0.00599454 and C4-160 *P* = 0.05524773). **e** Spatial distribution of GHRH expression in human hypothalamus from the HypoMap dataset. **f** Top MAGMA-prioritized genes contributing to genetic enrichment in mouse Ghrh neurons, showing expression specificity across mouse and human GHRH neurons and human pancreatic β-cells.

While direct experimental validation of hyperglycemia-induced transcriptional changes in human brain cells is not feasible, genome-wide association study (GWAS) data can address the issue indirectly by asking whether genes linked to glucose regulation in humans are statistically overrepresented among marker or stimulus-responsive genes in relevant mouse cell populations (Fig. 8a). Given the crucial role that the ARC/VMH plays in both energy and glucose homeostasis and to enhance marker gene identification, we integrated our single-cell data with another (ARC/VMH dataset), yielding a combined dataset of 194,153 ARC/VMH cells. In addition, we utilized summary statistics from fasting glucose and random glucose GWAS studies^43,44^. We next applied the CELLEX tool^45^ to identify genes specifically expressed by each cell population of the combined hypothalamus snRNA-seq dataset and applied the CELLECT tool to identify cell populations that exhibited a strong enrichment for genes in fasting and random glucose GWAS studies. Agnostically testing a total of 70 cell populations, we identified one enriched cell population: namely, *Ghrh*-expressing neurons (*P*_adj_ < 0.005; Fig. 8b and Supplementary Data 7). These findings are not exclusive, and larger GWAS studies on random and fasting glucose could potentially reveal additional cell types.

To confirm that the mouse-derived cell populations expressed marker genes expressed in human *Ghrh* neurons, we identified cells in the human HypoMap^46^ (a publicly available atlas of 433,369 cells across the hypothalamus) that transcriptionally resembled the mouse *Ghrh*-expressing cells. Two human hypothalamic cell populations, ‘C4-160’ and ‘C4-161’, mapped to the mouse *Ghrh* neuron population, and expressed marker genes that significantly overlapped with human fasting and random glucose GWAS genes (*P* < 0.05, *P* < 0.005, respectively; Fig. 8c, d). Spatial transcriptomics data from the human HypoMap confirmed that *GHRH* neurons also reside in the human ARC (Fig. 8e)^46^. Expectedly, marker genes of *GHRH* neurons from both species co-localized with fasting and random glucose-associated genetic variants, some of which also overlap with genes expressed in human pancreatic beta cells (Fig. 8f**)**.

Together, these analyses support a model wherein genes expressed in mouse and human *GHRH* neurons mediate in part the effect of fasting and random glucose-associated GWAS variants on blood glucose concentrations. Although best known for their role in control of the growth hormone axis, we view these findings as evidence of a possible role for *Ghrh* neurons in glucose homeostasis as well.

## Discussion

Although the glucose-responsive nature of some hypothalamic neurons has been known for decades, and while the brain is clearly involved in the adaptive response to a low blood glucose level (hypoglycemia), the question of whether and how the brain responds to hyperglycemia has yet to be fully understood. This study aimed to apply unbiased, discovery-driven transcriptomics to uncover new candidate cell types involved in glucose sensing and generate testable hypotheses for future work. Therefore, we employed snRNA-seq to create a cell-type-resolved map of transcriptional responses to hyperglycemia in two key brain areas for energy and glucose homeostasis,the ARC/VMH and DVC, and then determined how these responses are impacted by HFD-feeding, with modest weight gain. Our data reveal that in control diet-fed mice, astrocytes and oligodendrocytes show transcriptional responses to hyperglycemia that are conserved across these two brain areas. Despite our initial finding that astrocyte populations from the ARC/VMH and DVC are transcriptionally distinct, the response of these glial cells to hyperglycemia is conserved across brain regions. We also report that in response to hyperglycemia, certain neurons in the ARC/VMH exhibit cell-type-specific transcriptional changes suggestive of reduced activity. Strikingly, HFD inverted these hyperglycemia-induced transcriptional signatures, indicating that even modest weight gain causes a fundamental shift in how these brain regions sense and respond to an elevated blood glucose level.

Among dozens of uniquely identified hypothalamic neuronal cell types, our unbiased approach identified ARC *Ghrh* and *Agrp* neurons as being particularly responsive to sustained hyperglycemia. Interestingly, both neuronal subsets are activated in conditions of fuel deficit, including fasting, hypoglycemia and in response to the gastric hormone ghrelin (which is secreted when the stomach is empty). In this context, our finding that prolonged hyperglycemia (60 min) triggers distinct gene expression changes suggestive of reduced activity raises the interesting possibility that acute exposure to an oversupply of glucose is sufficient to override the response of these neurons to fuel depletion (despite having minimal impact on body fuel stores). Notably, prior work has shown that *Ghrh* neurons are activated by hypoglycemia, suggesting a physiological role in glucose sensing and regulation via growth hormone release^36^. Viewed through this lens, it is interesting to note that the transcriptional response of *Ghrh* and *Agrp* neurons to hyperglycemia is prevented or even inverted by HFD feeding. Specifically, whereas hyperglycemia suppresses activity-related genes in *Ghrh* neurons of control diet-fed mice, these same genes are upregulated in HFD-fed mice, suggesting a switch from glucose-inhibited to glucose-excited behavior. Whether and how this inversion impacts glucose homeostasis is an important unanswered question. For example, *Ghrh* neurons stimulate growth hormone release, which plays a role in glucose metabolism, and the effects of both fasting and hypoglycemia to increase growth hormone secretion (which involve activation of these neurons) are blunted in obese humans^47,48^. We further observed that gene signatures upregulated by hyperglycemia in control animals were downregulated in HFD-fed mice across both neuronal and glial populations, indicating a widespread reversal of normal mechanisms underlying detection of and responsiveness to hyperglycemia.

We acknowledge that earlier studies using acute, high‑dose carotid glucose boluses showed increased c‑fos expression across hypothalamic cell types, but this stimulus produces rapid and transient glucose fluctuations that differ markedly from the stable hyperglycemia induced in our glucose‑clamp paradigm^49^. Because neuronal activation markers such as c‑fos respond strongly to changes in glucose rather than steady levels, our design would not be expected to replicate these earlier findings. Thus, the lack of widespread neuronal activation signatures in our dataset is consistent with the distinct experimental conditions and physiological responses involved. Moreover, in the HFD to control diet comparison, we identified a smaller number of differentially expressed genes relative to Dreng et al^50^ and Campbell et al^51^, which most likely is due to our more conservative significance threshold (FDR < 0.05 vs. 0.1) and pseudobulk‑based testing to account for donor‑level dependence.

To assess the relevance of our findings in humans, we integrated GWAS data for glucose traits with our snRNA-seq results. We found that genetic variants associated with the fasting glucose level were enriched near genes expressed in *Ghrh* neurons, supporting the notion that these neurons could play a critical role in human glucose regulation. Interestingly, although our dataset included several hypothalamic populations classically linked to energy and glucose regulation, including *Pomc* and *Pnoc* neurons^52^, these cell types did not exhibit enrichment for genes located near glycemic trait-associated loci in GWAS. Potential explanations are that the potential glucose-sensing function of these neurons is not under genetic control, that current GWAS are not well-powered enough to implicate such genetic variants, or that their contribution becomes more apparent under different physiological or developmental conditions. This highlights the importance of agnostic, data-driven strategies like ours for uncovering non-obvious neuronal mediators of metabolic control and suggests that therapeutic targeting of specific hypothalamic circuits could represent a promising strategy for reversing the effects of obesity and T2D on glucose homeostasis.

Such considerations highlight the importance of future studies that clarify whether obesity-induced disruption of the neuronal response to hyperglycemia contributes to obesity-associated metabolic impairment and, if so, to identify underlying mechanisms. For example, could obesity-associated insulin resistance, which has been reported in hypothalamic astrocytes and other cell populations, blunt the transcriptional response of these cells to hyperglycemia? Future studies may also *i)* use in vivo calcium imaging or electrophysiology to validate the suggestive neuron activity changes in *Ghrh* and *Agrp* neurons observed at the transcriptional level; *ii)* investigate the role of insulin, leptin and other candidate mediators of (or contributors to) either the effect of hyperglycemia in control diet-fed animals or the altered response in the setting of obesity, particularly given that obesity markedly increases the circulating level of these hormones; *iii)* explore whether restoring normal *Ghrh* neuron function can improve glucose homeostasis in diet-induced obesity models, potentially through chemogenetic or optogenetic approaches; *iv)* conduct longitudinal studies to map the progression of transcriptional changes in the brain during the development of obesity and insulin resistance, and *v)* clarify both the significance of and mechanisms underlying the relative lack of DVC neuron responsiveness to hyperglycemia. Another important unanswered question is whether the effects of hyperglycemia on these hypothalamic neuronal populations are mediated directly (e.g., by activating cell-intrinsic glucose-sensing machinery) or indirectly (by activating circuits lying upstream).

Our comprehensive, cell-type-resolved map of hyperglycemia-induced transcriptional responses in the hypothalamus and brainstem provides evidence that key brain regions for fuel homeostasis can detect and respond to a hyperglycemic stimulus. We further demonstrate that *Ghrh* and *Agrp* neurons in the ARC/VMH are responsive to this stimulus, and that the response of these neurons is dramatically altered by HFD feeding. The extent to which the inverted transcriptional profiles of these neurons contribute to obesity-associated glucose metabolic impairment is an important unanswered question. Together, these findings offer insights into the central regulation of glucose metabolism and create potential targets for therapeutic intervention in obesity and T2D.

## Methods

### Mice

Male 12-week-old C57BL/6 J(WT) mice were purchased from Scanbur (Karlslunde, Denmark) for infusion studies. An additional six adult mice (3 males and 3 females) 12-weeks-old, breed in-house, C57BL/6JBomTac, were used for brain in situ hybridization (ISH) experiments. Animals were maintained under specific pathogen-free conditions in a temperature-controlled environment with 45/65% humidity and a 12:12 h light: dark cycle. Animals were allowed to acclimate in the animal facility for at least 7 days and then placed on HFD (Research Diets, D12492i) or a control diet (Research Diets, D12450Ji) for 8 weeks before surgery. After surgery, mice were individually housed with ad libitum access to water and a control diet or a high-fat diet was maintained unless otherwise stated. All procedures were performed with approved protocols from the Danish Animal Experiments Inspectorate permit number 2020-15-0201-00715 and the University of Copenhagen project number P22-581.

### Surgery

For glucose or saline infusion and blood collection during clamped hyperglycemia, catheters were implanted under aseptic conditions into the right external jugular vein (C20PU-MJV1458; Instech, USA) and the left common carotid artery (C10PU-MCA1459; Instech, USA), exteriorized in the scapular region and secured using a dual-channel vascular access button (VABM2B/25R25; Instech, USA) under general isoflurane anesthesia. Mice were subcutaneously injected preoperatively with Carprofen (5 mg/kg, Norodyl Vet, Scanvet, Denmark) for analgesia and a mixture of Lidocaine (7 mg/kg, Xylocain, AstraZeneca, UK) and Bupivacaine (7 mg/kg, Marcaine, Orifarm, Denmark) at the incision sites for local anesthesia. Mice were allowed to recover for 7 days after surgery.

### Hyperglycemic clamp infusion studies

After 1 week of recovery, food was removed four hours before the infusion (−240 min). At −120 min, mice were connected to a low-stress two-channel swivel/tether system (Glucose Clamp Configuration; Instech, USA), where mice were freely moving for the duration of the clamp. Blood glucose measurements were taken every 5 min over the clamp period with Contour Next glucose strips and a Contour XT blood glucose monitor. At −5, 5, 20, and 60 min, blood samples were taken to measure plasma insulin. Glucose infusion rate was modified throughout the clamp to maintain hyperglycemia. Insulin values where not clamped; therefore, in this context, the glucose-infusion rate cannot directly be informative of insulin sensitivity.

### Single-nucleus RNA-seq tissue processing

#### Tissue collection

At the time of sacrifice, cervical dislocation was performed, and the brain was rapidly removed and cut with a razor blade to separate the forebrain and hindbrain. The forebrain was placed into an ice-chilled coronal mouse brain slicer (Zivic Instruments, #BSMAS005-1, or ASI Instruments, RBM-2000C). A 1–1.5 mm thick coronal slice was obtained between the rostral and caudal ends of the Circle of Willis and transferred to ice-cold PBS. From this coronal section, the arcuate nuclei ventromedial nucleus were dissected, placed in a tube on dry ice, and stored at –80 °C. The dorsal vagal complex (DVC) was isolated from the hindbrain region of a coronal slice identified from the obex, placed in a separate pre-chilled tube, and likewise stored at –80 °C for subsequent analysis.

#### Single nuclei suspension

Frozen tissue was transferred to a 2 ml glass douncer (Sigma-Aldrich, T2690) with 1 ml cold Nuclei EZ lysis Buffer (Sigma-Aldrich, NUC101) and homogenized using pastel B. The homogenized tissue was incubated for five minutes on ice and was filtered through a pre-wet 40 um mini cell strainer (PluriSelect, 43) into a 2 ml Protein LoBind tube. An additional 300 µl EZ Lysis Buffer was used to wash the strainer. Samples were centrifuged at 1000 *g* for 10 min, at 4 °C, with the brake set to 1(to avoid disrupting the pellet). The supernatant was removed, and the pellet was resuspended in 500 µl Nuclei Buffer (PBS with 1% BSA(Sigma-Aldrich, SRE0036), 2 mM MgCl2 (Sigma-Aldrich M1028), 40 U/ml Protector RNase inhibitor (Sigma-Aldrich, 3335399001) and incubated on ice for 15 min. Next, samples were centrifuged at 1000 *g* for 10 min, 4 °C, brake set to 1. The supernatant was removed, and the pellet was gently resuspended in 100 µl Nuclei Buffer for hashtag antibody incubation.

#### Nuclei hashtags for multiplexing

TotalSeq nuclei hash tag antibody with DNA oligonucleotide (Bioledgend) 0.5 ug was added to each sample and incubated for 30 min on ice. The nuclei were washed by adding 1 ml of Nuclei Buffer and centrifuging for 10 min, 4 °C, 1000 x *g*, brake set to 1. The supernatant was discarded, and the pellet was resuspended in Nuclei Buffer with 0.5 ug of DAPI/ul (Chemometec) 200 µl for ARC/VMH samples and 500 µl for DVC samples.

#### Nuclei sorting

DAPI-positive nuclei were sorted (SH800S (Sony)) using a 70-um sorting chip, 2000–3000 nuclei per sample, directly into 18.8 ul RT Buffer B(10x Genomics, 2000165) for GEM generation.

### Single-nucleus RNA sequencing analysis

#### Library generation and sequencing

Single-cell cDNA libraries were generated using the 10x Genomics (USA) Chromium single-cell controller and the 3′ v2 Reagent Kit according to the manufacturer’s protocol. Single-cell libraries were sequenced on a NovaSeq 6000 platform with 8 samples on one flow cell to obtain 100, and 32-bp paired-end reads using the following read length: read 1, 26 cycles, read 2, 98 cycles, and i7 index, 8 cycles.

#### Sequencing alignment and UMI counting

10x Genomics Cellranger mkfastq (v6.0.1) was run to convert binary base call files to fastq files. If the hamming distance between any adapter sequence was less than three, barcode mismatches were not allowed during the conversion.

Illumina sequencing reads were aligned to the mouse-optimized reference genome (ref) with CellRanger (v.7). To remove ambient RNA, CellBender (v.0.2.0) was run on the raw CellRanger output.

Alignment of HTO was performed in sketch mode with salmon alevin(v1.5.0)^53^. Library type was defined as ISR protocol and by the read geometry as read 2, position 1–15, barcode geometry as read 1, position 1–16, and UMI geometry as read 1, position 17–26.

Cell calling was done on the counts matrix and was carried out in R. First, barcode ranks were identified with the barcodeRanks function from the package DropletUtils v1.10.3^54^. A Hartigan’s dip test was performed on counts. If the resulting *P* > 0.05, the null hypothesis is accepted that the data is unimodal, and cells above the inflection value were used in downstream analysis. If the resulting *P* < 0.05, a bimodal model was fitted to the counts from cells above the original inflection with

the Mclust function from the mclust package (v5.4.7)^55^. Barcode ranks were then calculated again on the subset of cells assigned to the cluster with the highest average gene count. Cells above the recalculated inflection were used in downstream analysis. Cells overlapping between the RNA and HTO libraries were imported into Seurat v4.0.0^56^. The HTO assay was CLR-normalized and scaled. Cell barcodes were de-multiplex using HTODmux function. A positive quantile threshold was determined by running the function from 0.05–0.99 in 0.01 increments to maximize the number of classified singlets. To remove intra-sample cell doublets, RNA counts were log-normalized, and PCA was performed. Doublets were inferred from the inter-sample doublets with the recoverDoublets function from the scDblFinder v1.4.0 packages.

After doublet removal, cells were filtered for >1000 gene expressed per cell and genes expressed in ten or more cells were included. Nuclei were further filtered for <1 percent mitochondria and ribosomal genes, and the neuronal and nonneuronal cells were labeled with cellannotarR, and the function isOutlier was used with flag nmads = 5 to remove outliers in the neuronal and nonneuronal cells individually. Cells were filtered with the top and bottom 1% of the cells with nCount_RNA and were removed from the dataset.

### Annotating cell populations

A combination of approaches was used to label the cell clusters. First, the neuronal cells, from the ARC/VMH and DVC, were separated by the HTOs, identifying which particular regions the nuclei originated from. For the ARC/VMH nuclei label transfer from a Campbell et al^51^. data set that was repossessed and Affinati et al^34^. Cell populations not in the original data set were further classified based on marker genes with a high expression specificity score (see details for CELLEX below) and a top marker using pseudobulk sampling and the edgeR tool to account for the coupled structure of cells originating from the same mice. The gene with the highest CELLEX score also overlapped with an edgeR marker gene for the given cluster; if a gene overlapped in clusters, one or two additional genes were added for specificity. To label the neuronal population for the DVC label transfer from Ludwig et al., was used^31^.

### Differential gene expression

Differential gene expression analysis was performed using pseudobulk sampling and the edgeR tool^22^. Pseudobulk expression matrices were constructed for each animal across each cell population. For cell populations to be included in the differential expression analysis, they must have 25 cells or more and contain four samples with at least two from each treatment group, and samples must contain at least two cells. For genes to be included, the gene must have an expression value above zero and be expressed in more than 10 percent of the cells in that cell population. The function filterByExpr() was run to reduce the impact of outlier counts on the estimate of dispersion, the estimateGLMRobustDisp() function in edgeR was run, followed by glmFit() and the likelihood ratio test was performed with glmLRT(). Benjamini-Hochberg procedure (FDR) was used to correct *p*-values for multiple testing genes with FDR < 0.05 were considered significant.

### Identification of Shared Transcriptional responses

To generate rank scores, we split upregulated and downregulated genes, transformed *P*-values by −log_10_ and assigned a positive sign to upregulated and a negative sign to downregulated genes. We sorted by absolute rank score in descending order. Genes were matched across the two datasets, retaining only genes present in both. The final ranked lists were provided to the RRHO2_initialize() function from the RRHO2 package in R, generating a 2D matrix of hypergeometric enrichment *p*-values at each rank cutoff^57^. We used the resultant RRHO2 object to visualize overlap between gene expression profiles under control *vs*. HFD, highlighting regions of significant concordance or discordance response.

### Gene Ontology analysis

Gene list functional enrichment was performed on the overlapping gene sets from the RRHO2 analysis using the gost() function from the R package gprofiler2. The function gost() was subsequently run with all three Gene Ontology databases, biological process, molecular function, cellular component and KEGG. *P*-value cutoff of 0.05 and term_size equal to or less than 500 was used to determine significant Gene Ontology terms. The MetaBrain network was used to predict which pathways are enriched for a set of differentially expressed genes^37^. Differentially expressed genes with an FDR *P*-value of <0.05 were input into the online tool (https://network.metabrain.nl/), molecular function terms were downloaded, and the overlap among cell types was determined.

### Estimation of neuron activity

We used a short list of immediate early gene expression (*Fosb*, *Npas4*, *Fos*, *Junb*, *Nr4a1*, *Arc*, *Egr2*, *Egr1*, *Maff*, *Ier2*, *Klf4*, *Dusp1*, *Gadd45g*, *Dusp5*, *Btg2*, *Ppp1r15a*, *Amigo3*) that have commonly been used as markers of neuronal activity and used the Seurat function addmodulescore().

### Estimation of Glucose Sensing

We used a short list of glucose-sensing genes (*Gck, Slc2a2, Abcc8, and Kcnj11*) that are commonly associated with glucose-sensing capacity in the pancreatic beta cell and used the UCell() score to avoid negative values^58^ on the inferred gene expression data by ALRA^35^.

### CELLECT Analysis

Single-cell enrichment analysis for random and fasting glucose was tested using two computational toolkits, CELLEX and CELLECT^45^. Cell-type expressing specificity (CELLEX) (https://github.com/perslab/CELLEX) was used to compute Expression Specificity profiles for genes across cell types in the ARC/VMH atlas and the DVC. Followed by running CELLEX, Cell-type Expression-specific integration for Complex Traits (CELLECT) was run with the summary statistics from two genome-wide association studies (GWAS).

### Quantitative western blot

ARC/VMH and DVC samples were collected as stated above under **Tissue collection**, and a western blot was performed as previously described^59,60^. ARC/VMH and DVC samples were homogenized by sonication in T-PER Tissue Protein Extraction Reagent (Thermo Scientific #78501) and centrifuged at 10,000 *g* for 15 min supernatant was collected and assayed for protein concentration using a BAC Protein Assay Kit (Thermo Scientific #23225). Lysates were mixed in the Li-Cor protein sample loading buffer (Li-Cor #928-40004) and heated to 100 °C for 5 min. Samples were run on a 10% Bis-Tris Criterion XT gel (Bio-Rad Laboratory Inc., Hercules, CA) at 200 volts for 25 min electrophoretic transferred at 100 V for 45 min. Anti- GFAP antibody (Abcam ab7260) was used 1:1000 followed by IRDye 800CW Goat anti-Rabbit (Licor #P/N 926-32211) 1:15,000.

### RNAscope in-situ hybridization

RNAscope fluorescent in situ hybridization was performed on brains from six adult mice (3 males, 3 females). Animals were sacrificed by cervical dislocation, and brains were immersion-fixed in 4% paraformaldehyde at 4 °C for 24 h, cryoprotected in 25% sucrose for 48 h, and frozen in isopentane on dry ice. Coronal cryosections (14 µm) spanning the arcuate nucleus were processed using the RNAscope Multiplex Fluorescent Assay (Advanced Cell Diagnostics) on a Leica BOND RX automated staining system according to the manufacturer’s protocol. Probes targeted *Ghrh* (Cat. No. 470998), Slc6a3 (315448-C3), *Gck* (400978-C2), and *Foxp2* (428798-C2), with standard positive and negative controls (Cat. Nos. 322988 and 323208). Slides were coverslipped with ProLong Diamond Antifade Mounting medium (Thermo Fisher Scientific) and stored protected from light at 4 °C until imaging. Fluorescent images of the arcuate nucleus (ARC) were acquired using a Zeiss epifluorescence microscope (Carl Zeiss Microscopy). For each animal, 2–3 representative images were captured from matched coronal sections at comparable bregma levels. Z-stack image series spanning the full thickness of each section were acquired and processed by maximum intensity projection of the six most representative planes to generate a single composite image for analysis. Imaging parameters, including laser power, detector gain, pinhole aperture, and exposure settings, were held constant across all images within staining groups (*Slc6a3/Gck, Ghrh/Gck, Ghrh/Foxp2*) to enable direct comparison of fluorescence intensity. Quantitative image analysis was performed using QuPath (v0.5.x)^61^. Regions of interest corresponding to the ARC were manually delineated based on anatomical landmarks with reference to the Paxinos and Franklin mouse brain atlas. Cell detection was performed using the DAPI channel to segment nuclei, and fluorescence intensity features were extracted for each cell across all channels. Cells expressing *Ghrh* and *Slc6a3* were classified using single-measurement intensity thresholds. Thresholds were determined empirically based on inspection of intensity distributions and measurement maps. For targets with punctate expression patterns (*Gck* and *Foxp2*), subcellular spot detection was used, with parameters optimized to identify individual fluorescent puncta. Classification thresholds were validated by visual comparison to raw fluorescence images, and classifiers were saved for consistent application across all samples. Individual classifiers were combined into a composite classifier to enable multi-label classification of each cell. The composite classifier was applied uniformly across all images to generate all combinations of marker expression. Detection measurements and classification outputs were exported for downstream analysis in R.

### Statistics

For body weight, blood glucose, and plasma insulin data that was collected repeatedly on each individual animal over time a linear mixed model including fixed effect of treatment and day and random effects of animal. Linear mixed models were conducted with the R statistical package “nlme”, or the equivalent nonparametric test using the R statistical package “nparLD”^62^ and followed up by post hoc analysis using nparcomp^63^. Probability values less than 0.05 were considered statistically significant. Statistical significance between two groups was assessed using a two-sided two-sample Yuen’s trimmed mean t-test and Welch’s two-sample t-test. Correlation was assessed using a two-sided Kendall’s rank correlation test.

### Reporting summary

Further information on research design is available in the Nature Portfolio Reporting Summary linked to this article.

## Supplementary information

Supplementary Information

Peer Review file

Description of Additional Supplementary Files

Supplementary Data 1

Supplementary Data 2

Supplementary Data 3

Supplementary Data 4

Supplementary Data 5

Supplementary Data 6

Supplementary Data 7

Reporting Summary

## Source data

Source Data 1

Source Data 2

Source Data 3

Source Data 4

Source Data 5

Source Data 6

Source Data 7

Source Data 8

Source Data 9

Source Data 10

Source Data 11

Source Data 12

Source Data 13

Source Data 14

Source Data 15

Source Data 16

Source Data 17

## Data Availability

All snRNA-seq data are available in the Gene Expression Omnibus database under the accession code GSE262411. Source data are provided as a Source Data file. Source data are provided with this paper.

## Code availability

All code can be found at https://github.com/perslab/Brown-2025.
