# Supplementary material for: Single-cell profiling identifies obesity-disrupted brain glucose sensing
Source: Nat Commun. 2026 Aug 31;17:10342. doi: 10.1038/s41467-026-77116-9 (PMC13623956; doi:10.1038/s41467-026-77116-9)
Supplement: Supplementary file 1 [file 41467_2026_77116_MOESM11_ESM.pdf]

nature portfolio

Corresponding author(s): Tune H Pers

Last updated by author(s): 07-9-2026

Reporting Summary

Nature Portfolio wishes to improve the reproducibility of the work that we publish. This form provides structure for consistency and transparency in reporting. For further information on Nature Portfolio policies, see our Editorial Policies and the Editorial Policy Checklist.

Statistics

For all statistical analyses, confirm that the following items are present in the figure legend, table legend, main text, or Methods section.

n/a	Confirmed	
☐	☒	The exact sample size (n) for each experimental group/condition, given as a discrete number and unit of measurement
☐	☒	A statement on whether measurements were taken from distinct samples or whether the same sample was measured repeatedly
☐	☒	The statistical test(s) used AND whether they are one- or two-sided<br>*Only common tests should be described solely by name; describe more complex techniques in the Methods section.*
☐	☒	A description of all covariates tested
☐	☒	A description of any assumptions or corrections, such as tests of normality and adjustment for multiple comparisons
☐	☒	A full description of the statistical parameters including central tendency (e.g. means) or other basic estimates (e.g. regression coefficient) AND variation (e.g. standard deviation) or associated estimates of uncertainty (e.g. confidence intervals)
☐	☒	For null hypothesis testing, the test statistic (e.g. F, t, r) with confidence intervals, effect sizes, degrees of freedom and P value noted<br>*Give P values as exact values whenever suitable.*
☒	☐	For Bayesian analysis, information on the choice of priors and Markov chain Monte Carlo settings
☐	☒	For hierarchical and complex designs, identification of the appropriate level for tests and full reporting of outcomes
☒	☐	Estimates of effect sizes (e.g. Cohen's d, Pearson's r), indicating how they were calculated

Our web collection on statistics for biologists contains articles on many of the points above.

Software and code

Policy information about availability of computer code

Data collection	No software was used for data collection.
Data analysis	All codes for snRNA-seq data analysis are available at https://github.com/perslab/Brown-2025. All code for analysis snRNA-seq data were analyzed using Cell Ranger mkfastq v6.0.1 (10x Genomics), Cell Ranger v7 (10x Genomics), CellBender, salmon alevin v1.5.0, R v3.5.3 (R Core Team), RStudio v1.1.38, DropletUtils v1.10.3, mclust v5.4.7, Seurat v4.0.0, scDblFinder v1.4.0, edgeR, RRHO2, gprofiler2, ALRA, UCell, CELLEX, CELLECT, cellannotaR, nlme, nparLD, and nparcomp.

For manuscripts utilizing custom algorithms or software that are central to the research but not yet described in published literature, software must be made available to editors and reviewers. We strongly encourage code deposition in a community repository (e.g. GitHub). See the Nature Portfolio guidelines for submitting code & software for further information.

Data

Policy information about availability of data

All manuscripts must include a data availability statement. This statement should provide the following information, where applicable:

- Accession codes, unique identifiers, or web links for publicly available datasets
- A description of any restrictions on data availability
- For clinical datasets or third party data, please ensure that the statement adheres to our policy

All snRNA-seq data generated in this study are available through the NCBI Gene Expression Omnibus (GEO) under Super Series accession number GSE262411

(https://www.ncbi.nlm.nih.gov/geo/query/acc.cgi?acc=GSE262411). This study also used publicly available datasets including 'A Molecular Census of Arcuate Hypothalamus and Median Eminence Cell Types' (GSE93374; https://www.ncbi.nlm.nih.gov/geo/query/acc.cgi?acc=GSE93374), 'Cross-species analysis defines the conservation of anatomically segregated VMH neuron populations'(GSE172207; https://www.ncbi.nlm.nih.gov/geo/query/acc.cgi?acc=GSE172207), and GWAS studies from (https://www.nature.com/articles/s41467-023-36013-1, summary statistic can be down loaded at https://cnsgenomics.com/data/qiao_et_al_2023_nc/ and https://www.nature.com/articles/s41588-023-01462-3#data-availability, summary statistic can be down loaded at https://www.ebi.ac.uk/gwas/publications/37679419) .The data supporting the findings of this study are available within the article and its supplementary information files or from the corresponding authors upon request. Source data are provided with this paper including data underlying Figs. 1, 2, 3, 4, 5, 6, 7, 8.

Research involving human participants, their data, or biological material

Policy information about studies with human participants or human data. See also policy information about sex, gender (identity/presentation), and sexual orientation and race, ethnicity and racism.

Reporting on sex and gender	n/a
Reporting on race, ethnicity, or other socially relevant groupings	n/a
Population characteristics	n/a
Recruitment	n/a
Ethics oversight	n/a

Note that full information on the approval of the study protocol must also be provided in the manuscript.

Field-specific reporting

Please select the one below that is the best fit for your research. If you are not sure, read the appropriate sections before making your selection.

☒ Life sciences ☐ Behavioural & social sciences ☐ Ecological, evolutionary & environmental sciences

For a reference copy of the document with all sections, see nature.com/documents/nr-reporting-summary-flat.pdf

Life sciences study design

All studies must disclose on these points even when the disclosure is negative.

Sample size	Sample size for each study was determined based on published (Campbell et al., 2017) and prior work by the authors including surgical limitations.
Data exclusions	Due to the nature of the surgical procedure if the mice died during the clamp procedure likely caused by a blood clot there it was excluded from the study n=2.
Replication	Due to the nature of the clamp and snRNA-seq there was no attempts to replicate the entire study.
Randomization	Allocation of mice to groups were based on body weight to match the mean of both groups for the specific diet.
Blinding	Researcher performing the clamp were not blind to the treatment groups. Researcher demuliplexing the snRNA-seq data where bind to the treatment groups

Reporting for specific materials, systems and methods

We require information from authors about some types of materials, experimental systems and methods used in many studies. Here, indicate whether each material, system or method listed is relevant to your study. If you are not sure if a list item applies to your research, read the appropriate section before selecting a response.

Materials & experimental systems

n/a	Involved in the study
☐	☒ Antibodies
☒	☐ Eukaryotic cell lines
☒	☐ Palaeontology and archaeology
☐	☒ Animals and other organisms
☒	☐ Clinical data
☒	☐ Dual use research of concern
☒	☐ Plants

Methods

n/a	Involved in the study
☒	☐ ChIP-seq
☒	☐ Flow cytometry
☒	☐ MRI-based neuroimaging

Antibodies

Antibodies used	Anti- GFAP antibody (Abcam ab7260) was used 1:1000 follow by IRDye 800CW Goat anti-Rabbit (Licor #P/N 926-32211) 1:15,000.
Validation	Anti- GFAP has 1643 citations included on the manufactures website (https://www.abcam.com/en-us/products/primary-antibodies/gfap-antibody-astrocyte-marker-ab7260)

Animals and other research organisms

Policy information about studies involving animals; ARRIVE guidelines recommended for reporting animal research, and Sex and Gender in Research

Laboratory animals	Male 12-week-old C57BL/6J(WT) mice were purchased from Scanbur (Karlslunde, Denmark). An additional six adult mice (3 males and 3 females) 12-weeks-old breed C57BL/6JBomTac inhouse were used for brain in situ hybridization (ISH) experiments. Animals were maintained under specific pathogen-free conditions in a temperature-controlled environment with 45/65% humidity and a 12:12 h light: dark cycle. Animals were allowed to acclimate in the animal facility for at least 7 days and then placed on HFD (Research Diets, D12492i) or a control diet (Research Diets, D12450Ji) for 8 weeks before surgery. After surgery, mice were individually housed and ad libitum access to water and control diet or high-fat diet was maintained unless otherwise stated.
Wild animals	n/a
Reporting on sex	Male and female 12-week-old C57BL/6J(WT) mice were used for this study.
Field-collected samples	n/a
Ethics oversight	All procedures were performed with approved protocols from the The Danish Animal Experiments Inspectorate permit number 2020-15-0201-00715 and the University of Copenhagen project number P22-581.

Note that full information on the approval of the study protocol must also be provided in the manuscript.

Plants

Seed stocks	*Report on the source of all seed stocks or other plant material used. If applicable, state the seed stock centre and catalogue number. If plant specimens were collected from the field, describe the collection location, date and sampling procedures.*
Novel plant genotypes	*Describe the methods by which all novel plant genotypes were produced. This includes those generated by transgenic approaches, gene editing, chemical/radiation-based mutagenesis and hybridization. For transgenic lines, describe the transformation method, the number of independent lines analyzed and the generation upon which experiments were performed. For gene-edited lines, describe the editor used, the endogenous sequence targeted for editing, the targeting guide RNA sequence (if applicable) and how the editor was applied.*
Authentication	*Describe any authentication procedures for each seed stock used or novel genotype generated. Describe any experiments used to assess the effect of a mutation and, where applicable, how potential secondary effects (e.g. second site T-DNA insertions, mosiacism, off-target gene editing) were examined.*
