# Supplementary material for: Single-cell profiling identifies obesity-disrupted brain glucose sensing
Source: Nat Commun. 2026 Aug 31;17:10342. doi: 10.1038/s41467-026-77116-9 (PMC13623956; doi:10.1038/s41467-026-77116-9)
Supplement: Supplementary file 2 [file 41467_2026_77116_MOESM1_ESM.pdf]

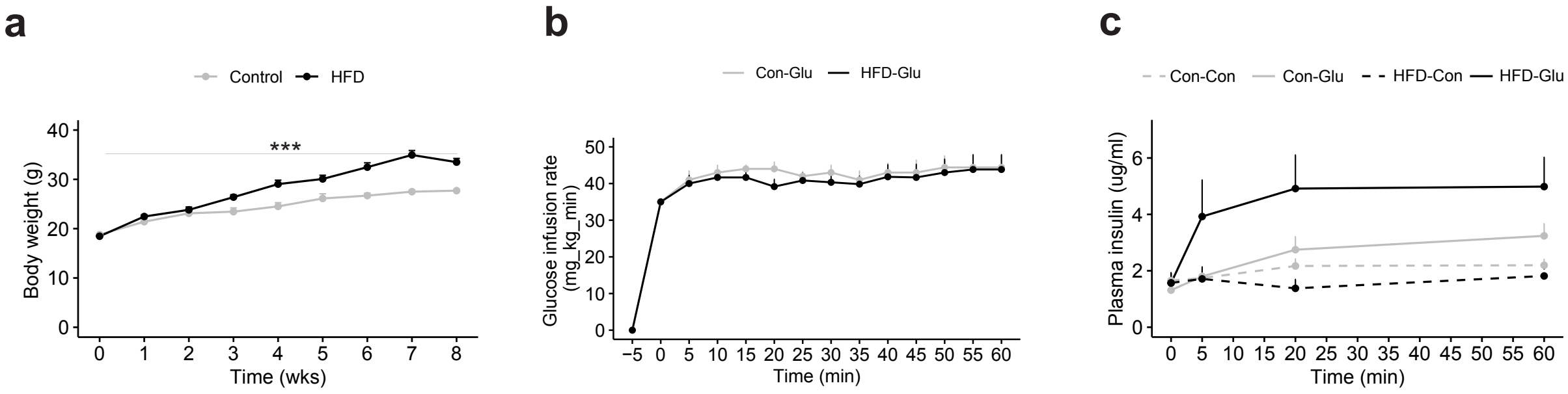

Supplementary Figure 1: Physiological parameters during modified hyperglycemic clamp. a, Body weight trajectories over 8 weeks in mice fed control diet (Con, n=9) or high-fat diet (HFD, n=10) (repeated measures nparLD ANOVA test statistic=61.16, df1 = 1, df2=17.68, P=3.8 e-07). **b**,Glucose infusion rates during hyperglycemic clamp in control diet (Con-Glu) and high-fat diet (HFD-Glu) mice. **c,** Plasma insulin levels measured at 0, 5, 20, and 60 minutes during the clamp period across all treatment groups.

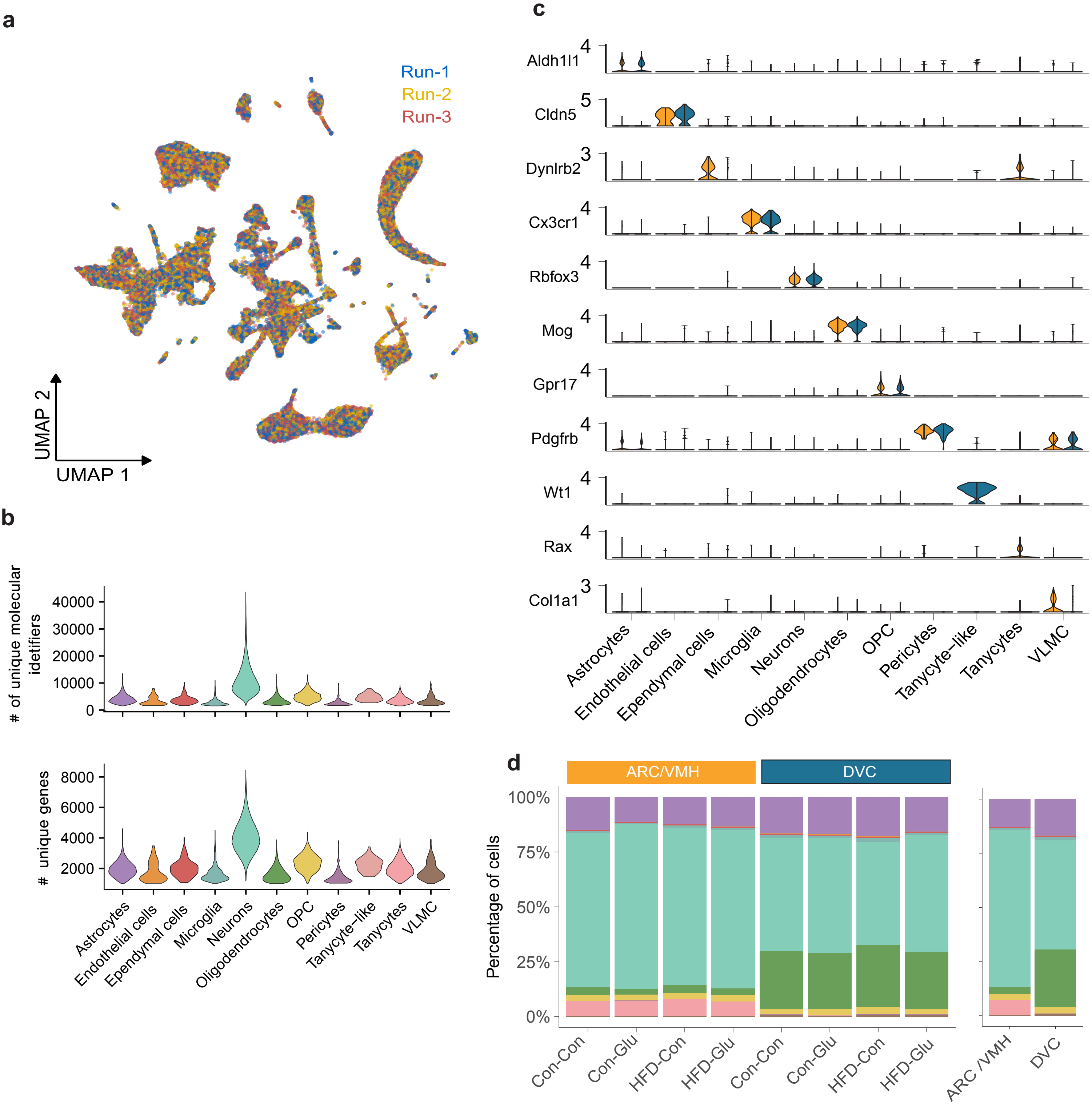

Supplementary Figure 2: Quality control metrics for single-nucleus RNA sequencing analysis. a, UMAP visualization of single-nucleus RNA-seq data colored by sequencing batch: Run-1 (blue), Run-2 (yellow), and Run-3 (red). b, Violin plots showing distribution of unique molecular identifiers and genes detected per nucleus across cell types. c, Violin plots displaying cell type-specific marker gene expression in ARC/VMH (orange) and DVC (blue). d, Distribution of cell types shown as percentage of total nuclei across treatment groups and brain regions (ARC/VMH and DVC).

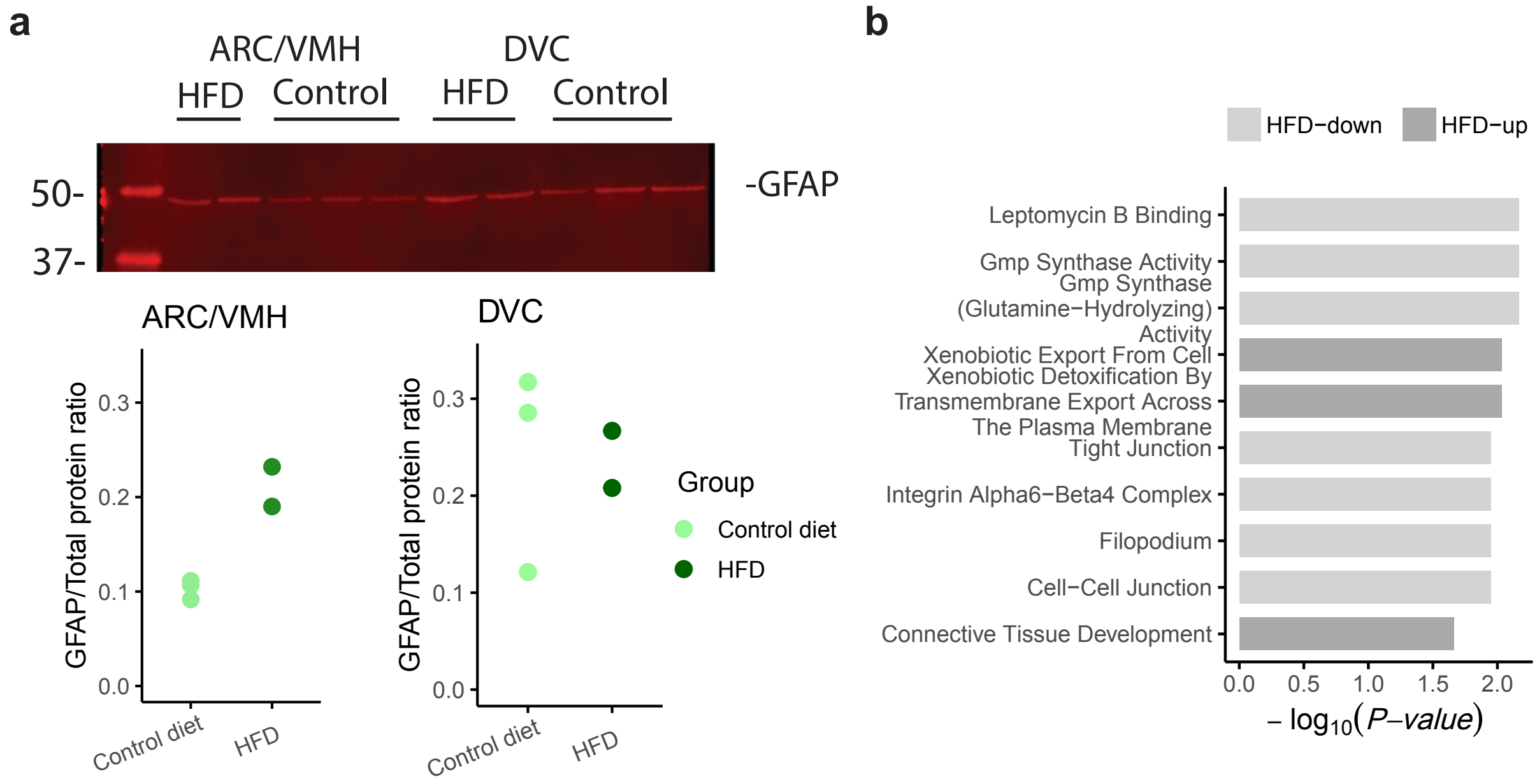

Supplementary Figure 3: High fat diet feeding alters transcriptional profiles in hypothalamic astrocytes. a, GFAP protein levels assessed by Western blot in the ARC/VMH and DVC of control diet- and HFD-fed mice (Control, n = 3; HFD, n = 2). One experimental replicate. b, Gene Ontology enrichment analysis of genes upregulated (HFD-up, dark grey) and downregulated (HFD-down, light grey) in ARC/VMH astrocytes identified by edgeR pseudobulk differential expression analysis (Benjamini–Hochberg-adjusted FDR < 0.05). Functional enrichment analysis was performed using g:Profiler (gost) with a cell type-specific background gene set. Statistical significance was determined using hypergeometric tests with Benjamini–Hochberg correction for multiple testing (FDR < 0.05).

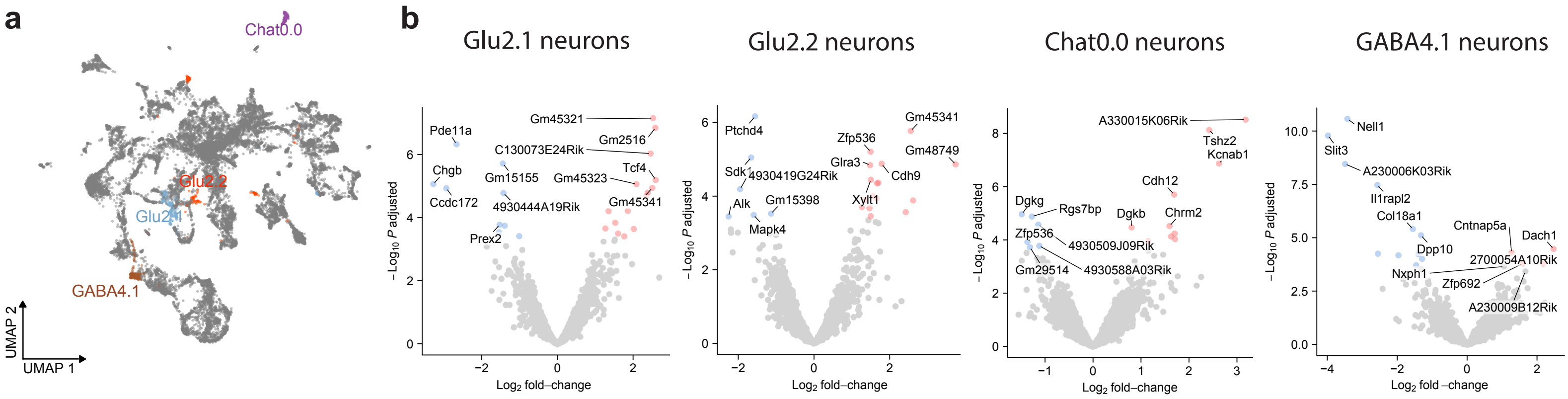

Supplementary Figure 4: Cell type-specific transcriptional responses to high fat diet feeding in DVC neurons. a, UMAP visualization of DVC neurons with key populations highlighted: Glu2.1, Glu2.2, Chat0.0, and GABA4.1 neurons. b, Volcano plots of differentially expressed genes Benjamini-Hochberg -adjusted edgeR likelihood ratio tests FDR <0.05 comparing HFD vs. control diet fed mice across four major DVC neuronal populations, with significant changes shown in red (upregulated) and blue (downregulated).

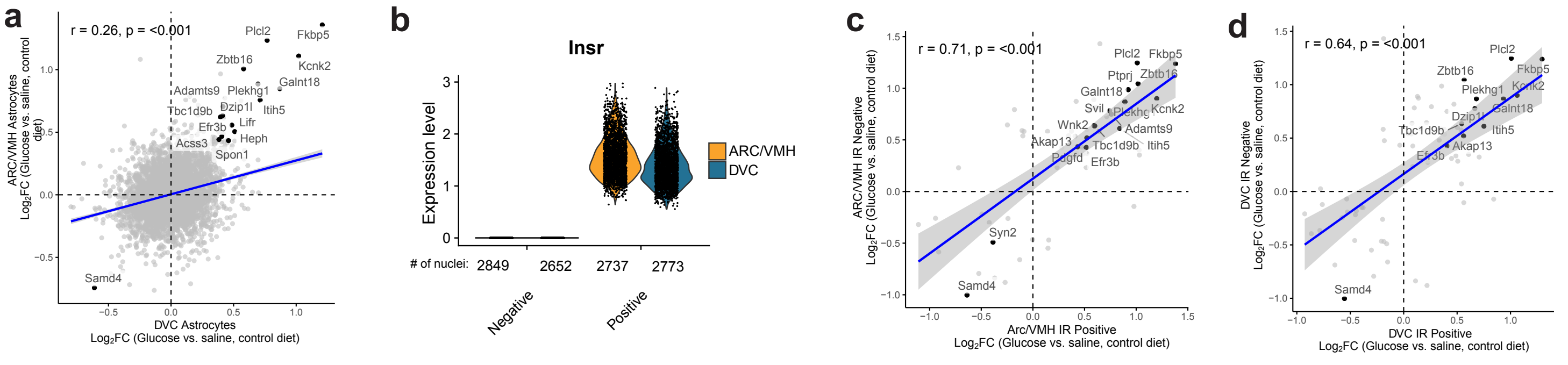

Supplementary Figure 5: Conserved brain region-specific and insulin receptor-dependent astrocyte responses to glucose in lean mice. a, Correlation analysis of glucose-induced transcriptional changes (glucose vs. saline) between ARC/VMH and DVC astrocytes in control diet mice, with overlapping significant genes (FDR <0.05) labeled. b, Distribution of insulin receptor (Insr) expression in insulin receptor-positive and -negative astrocytes from ARC/VMH and DVC regions. c, Correlation of glucose-induced transcriptional changes between insulin receptor-positive and -negative astrocytes in ARC/VMH, with overlapping significant genes (FDR <0.05) labeled. d, Correlation of glucose-induced transcriptional changes between insulin receptor-positive and -negative astrocytes in DVC, with overlapping significant genes (FDR <0.05) labeled.

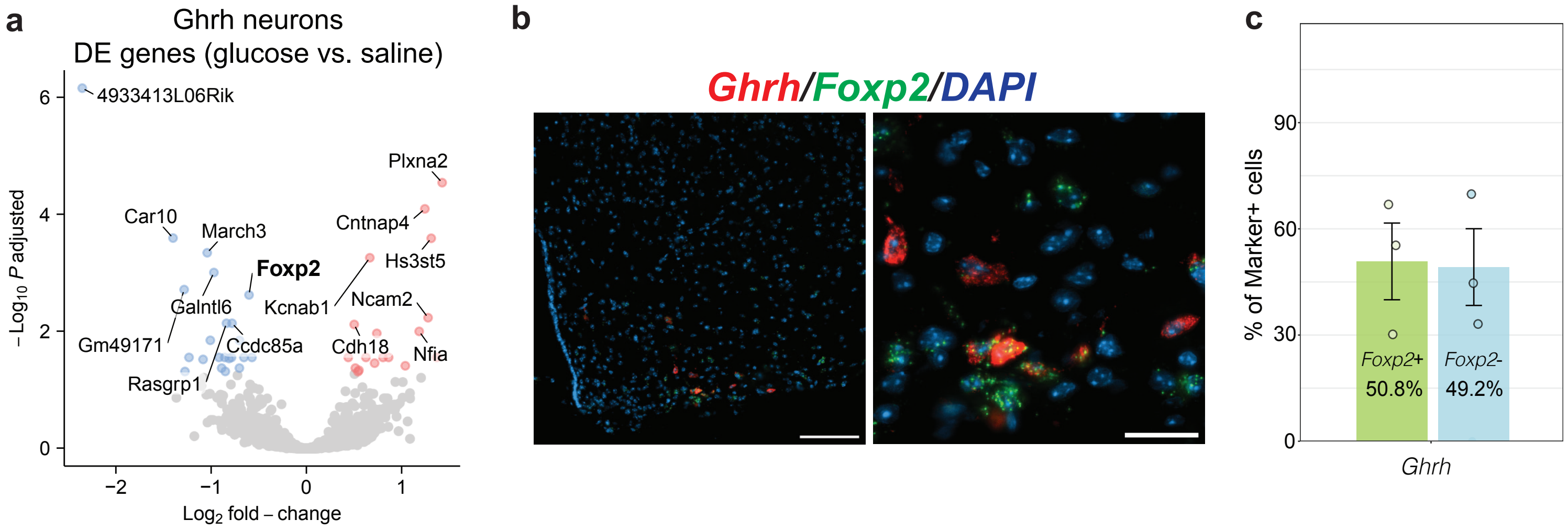

Supplementary Figure 6: Co-expression of Ghrh and Foxp2 in the ARC. a, Volcano plot showing differentially expressed genes in Ghrh neurons from glucose vs. saline infusion in control diet-fed mice. Differentially expressed genes (FDR<0.05) are shown in red for genes unregulated by glucose and blue for genes down regulated by glucose. Foxp2 is identified among glucose regulated transcripts. b, Representative RNAscope fluorescent in situ hybridization images from coronal hypothalamic sections of wild-type mixed-sex mice (n = 3), showing Ghrh (red), Foxp2 (green), and DAPI (blue) expression in the ARC/VMH. Low- and high-magnification are shown. Scale bars: 100 µm (left), 25 µm (right) c, Quantification showing approximately half of Ghrh+ cells co-express Foxp2 (Foxp2+: 50.8%; Foxp2-:49.2%). Individual data points represent biological replicates; bars show mean ± s.e.m.

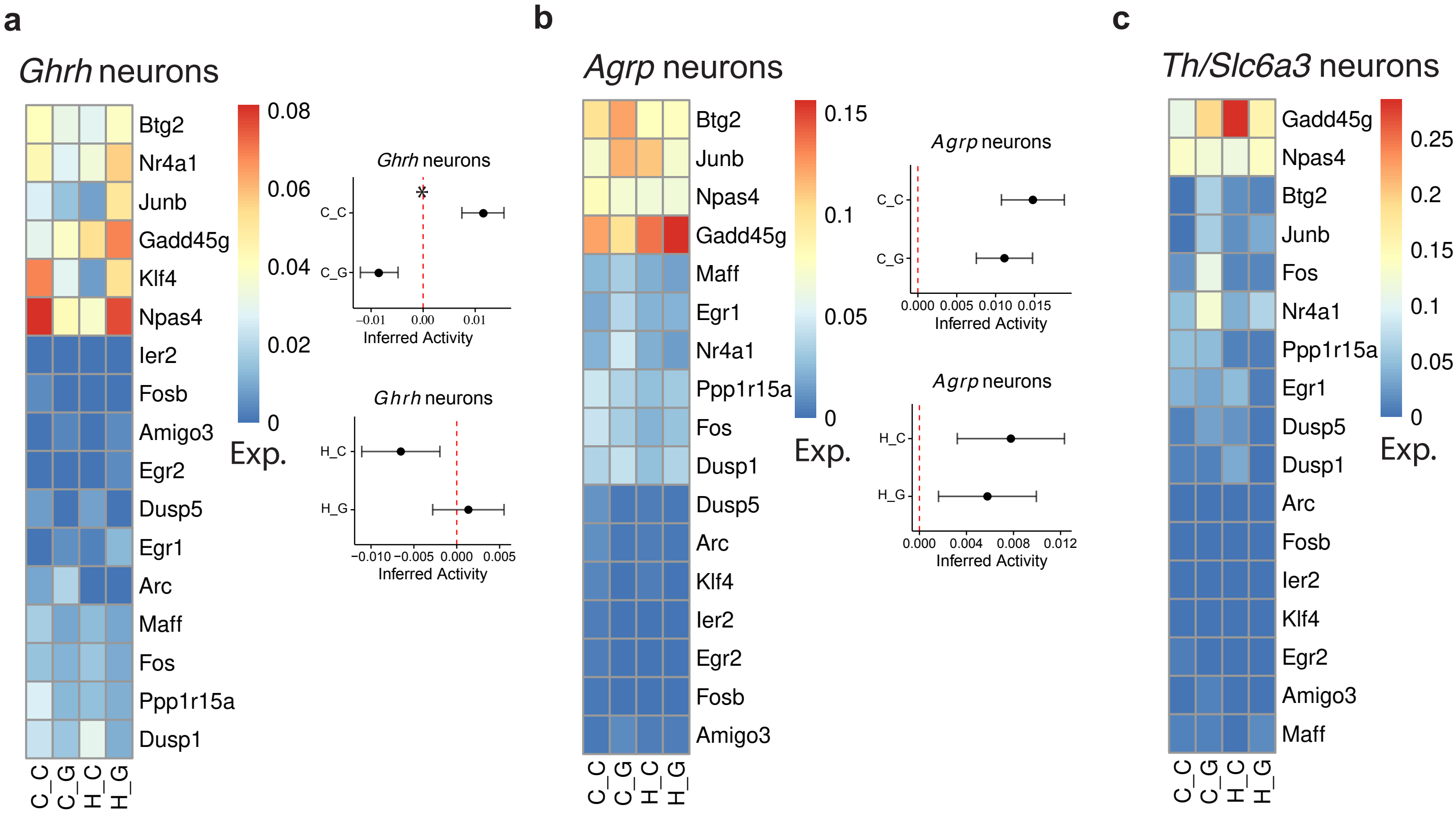

Supplementary Figure 7: Immediate early gene activation and inferred activity changes in Ghrh and Agrp neurons. a, Heatmap showing normalized expression of immediate early genes across all four treatment groups (C_C,C_G,H_C,H_G) in Ghrh neurons. Right average inferred neuronal activity estimate for all treatment groups top Control-fed (FDR<0.05) and bottom HFD-fed. b, Heatmap showing normalized expression of immediate early genes across all four treatment groups (C_C,C_G,H_C,H_G) in Agrp neurons. Right average inferred neuronal activity estimate for all treatment groups top Control-fed and bottom HFD-fed. c, Heatmap showing normalized expression of immediate early genes across all four treatment groups (C_C,C_G,H_C,H_G) in Th/Slc6a3 neurons. *Estimated marginal means were calculated from a linear mixed-effects model for each treatment group. Error bars represent ± SE. FDR < 0.05 for the glucose effect within diet. Exp. = expression

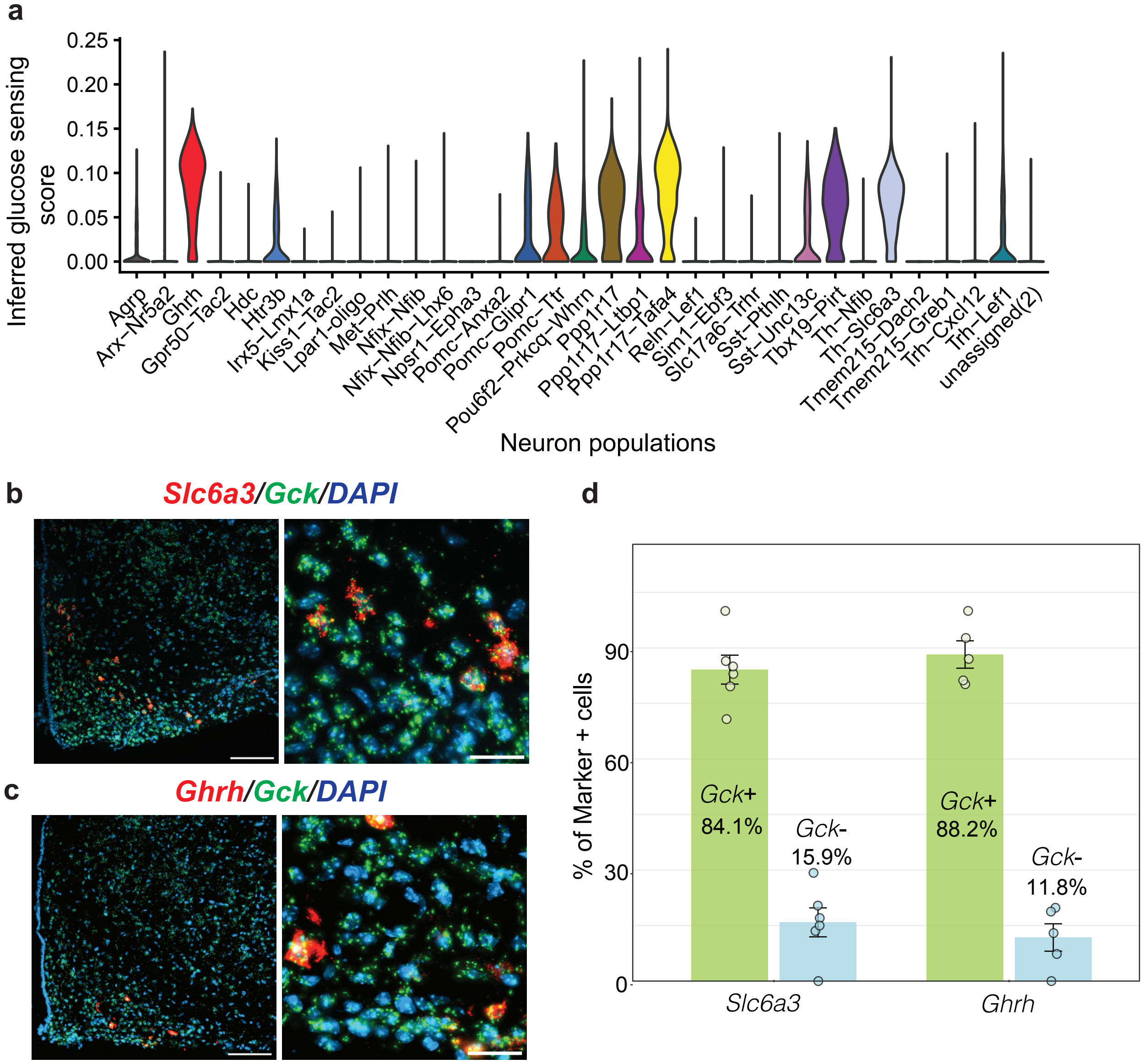

Supplementary Figure 8: Glucose sensing analysis and co-expression of Gck, Slc6a3 and Ghrh in the ARC. a,Glucose sensing score based on expression of key genes (Gck, Slc2a2, Abcc8, and Kcnj11) across ARC/VMH neuronal populations. b, Representative RNAscope fluorescent in situ hybridization images from coronal hypothalamic sections of wild-type mixed-sex mice (n = 5-6), showing Slc6a3 (red), Gck (green), and DAPI (blue) c, Ghrh (red), Gck (green) and DAPI (blue) expression in the ARC/VMH. Low- and high-magnification are shown. Scale bars: 100 μm (left), 25 μm (right) d, Quantification showing percentage of Slc6a3+ cells co-express Gck (Gck+: 84.1%; Gck-:15.9%) and Ghrh+ cells co-express Gck (Gck+: 88.2%; Gck-:11.8%). Individual data points represent biological replicates; bars show mean ± s.e.m.

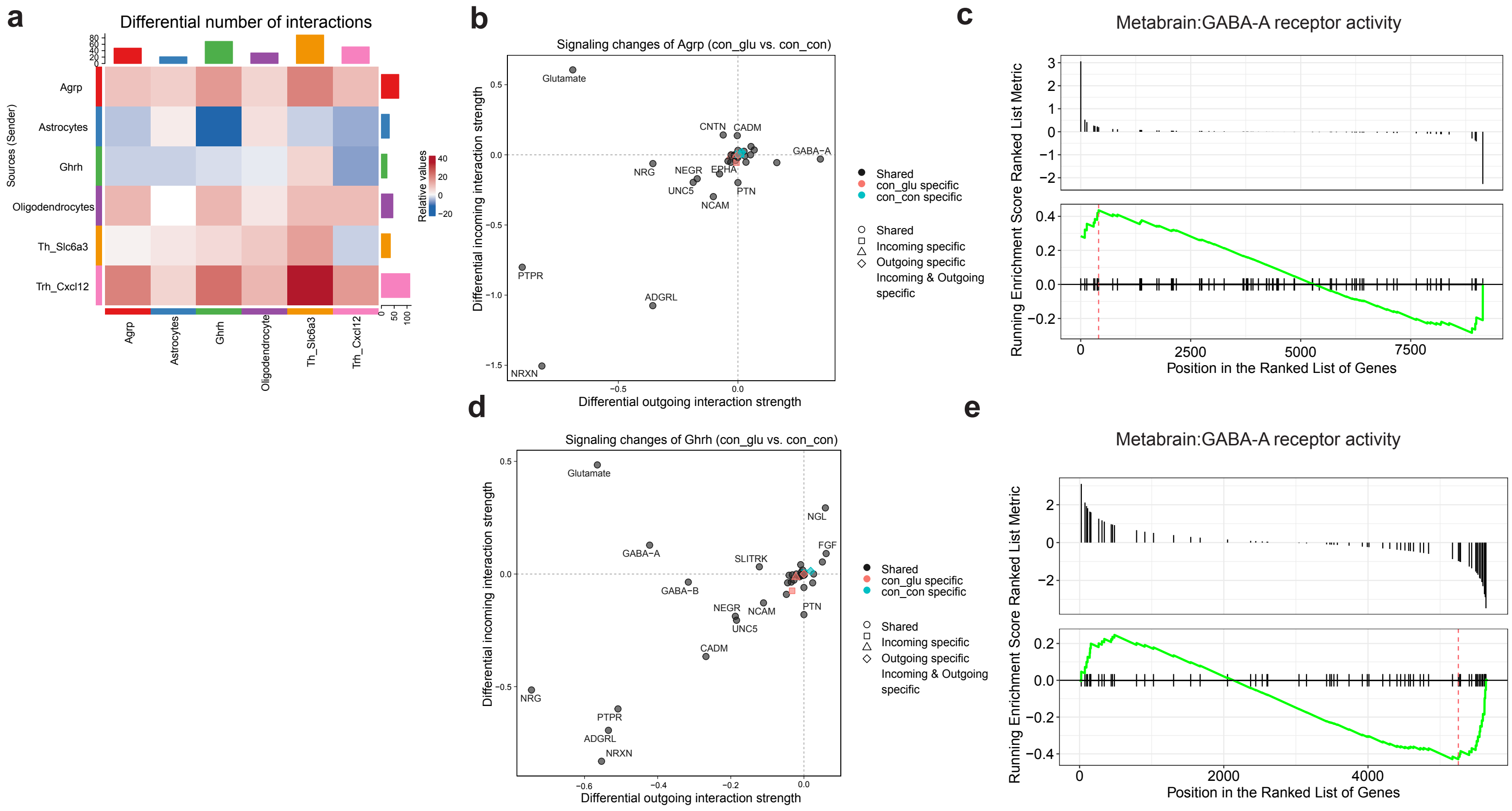

Supplementary Figure 9: Analysis of cell-cell signaling in Agrp and Ghrh neurons. a, Heatmap of predicted differential ligand-receptor interactions between neuronal and glial populations, with interaction strength scaled and color-coded by source and target cell type. **b,** Differential signaling strength in Agrp neurons comparing glucose vs. saline conditions in control diet fed mice, with arrows indicating signaling direction and node colors showing incoming, outgoing, or bidirectional changes. **c,** Gene set enrichment analysis (GSEA) of GABA-A receptor activity pathway in Agrp neurons, with enrichment score running sum (green line) and gene positions (black ticks) shown. **d,** Differential signaling strength in Ghrh neurons comparing glucose vs. saline conditions in control diet mice. **e,** Gene set enrichment analysis (GSEA) of GABA-A receptor activity pathway in Ghrh neurons.

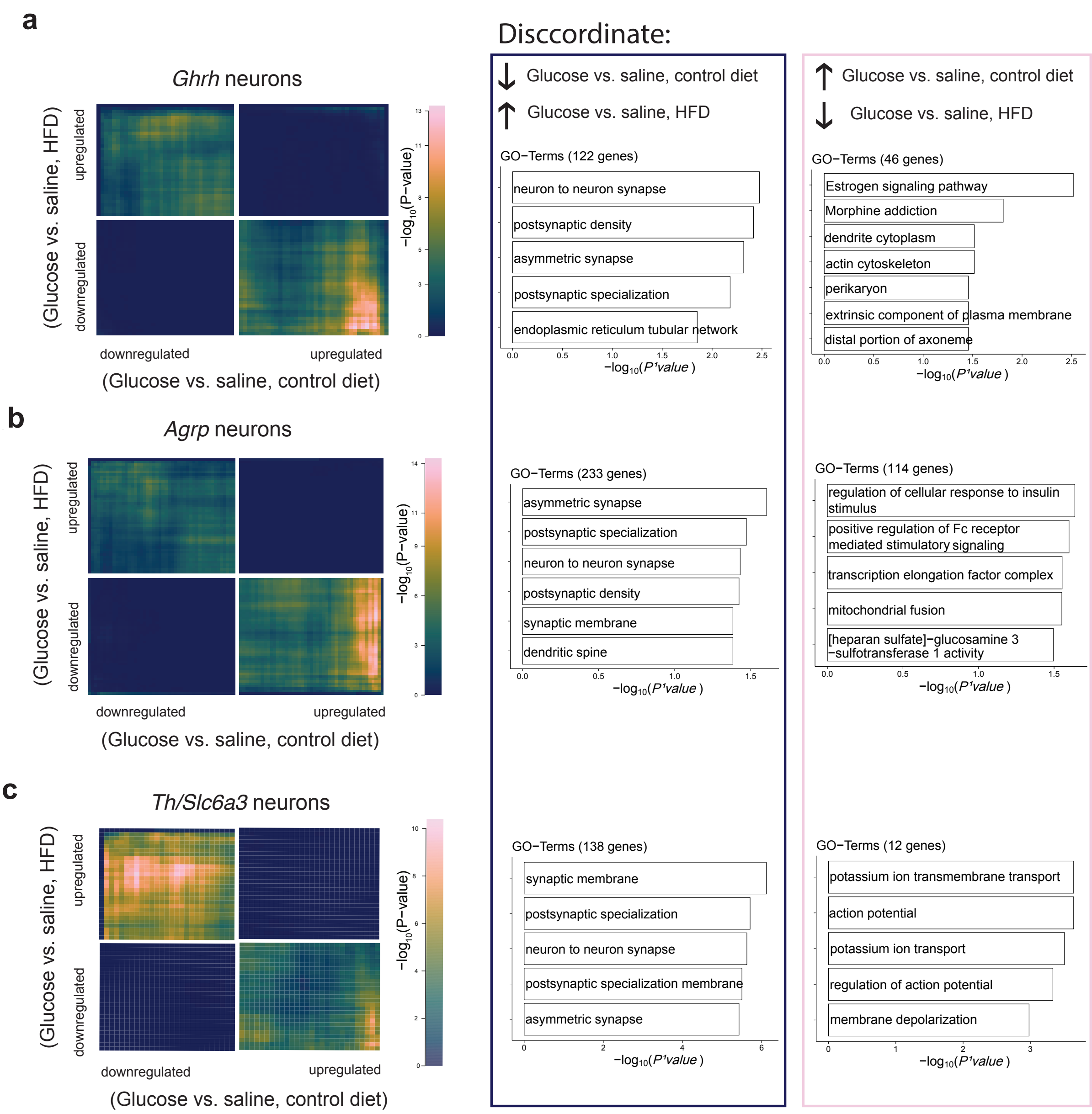

Supplementary Figure 10: High fat diet feeding reverses hyperglycemia-induced transcriptional responses in hypothalamic neurons. a, b, c, Rank-rank hypergeometric overlap analysis of glucose-responsive genes between control and high-fat diet conditions in Ghrh, Agrp, and Th/Slc6a3 neurons, with associated GO terms for genes showing reversed regulation (downregulated in control diet but upregulated in high-fat diet, and vice versa) performed using g:Profiler (gost) with a cell type specific background gene set. Statistical significance was determined using hypergeometric tests with Benjamini–Hochberg-adjusted FDR < 0.05.

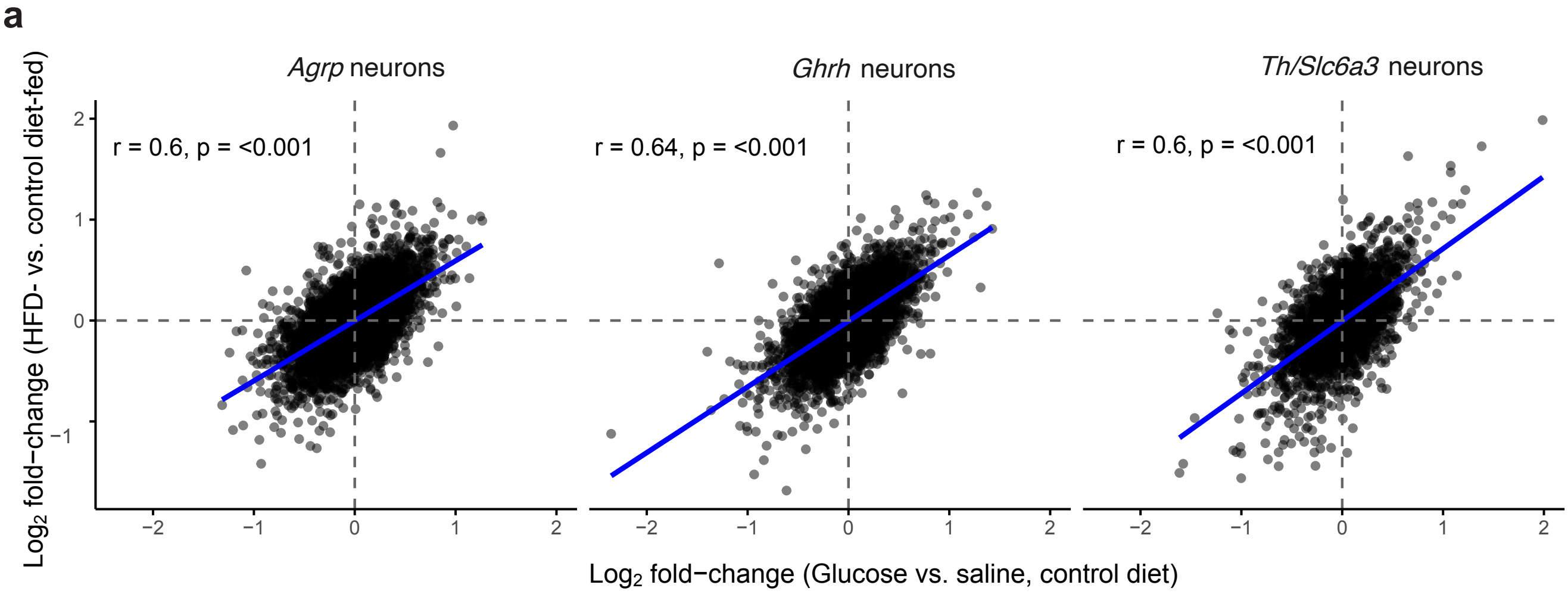

Supplementary Figure 11: Correlation between transcriptional responses to HFD feeding and acute hyperglycemia: a,Kendall correlation analysis of differentially expressed genes comparing HFD vs. control-fed mice (y-axis) against glucose vs. saline responses in control diet mice (x-axis) for Agrp, Ghrh, and Th/Slc6a3 neuronal populations.
