# Supplementary material for: Single-cell profiling identifies obesity-disrupted brain glucose sensing
Source: Nat Commun. 2026 Aug 31;17:10342. doi: 10.1038/s41467-026-77116-9 (PMC13623956; doi:10.1038/s41467-026-77116-9)

GFAP

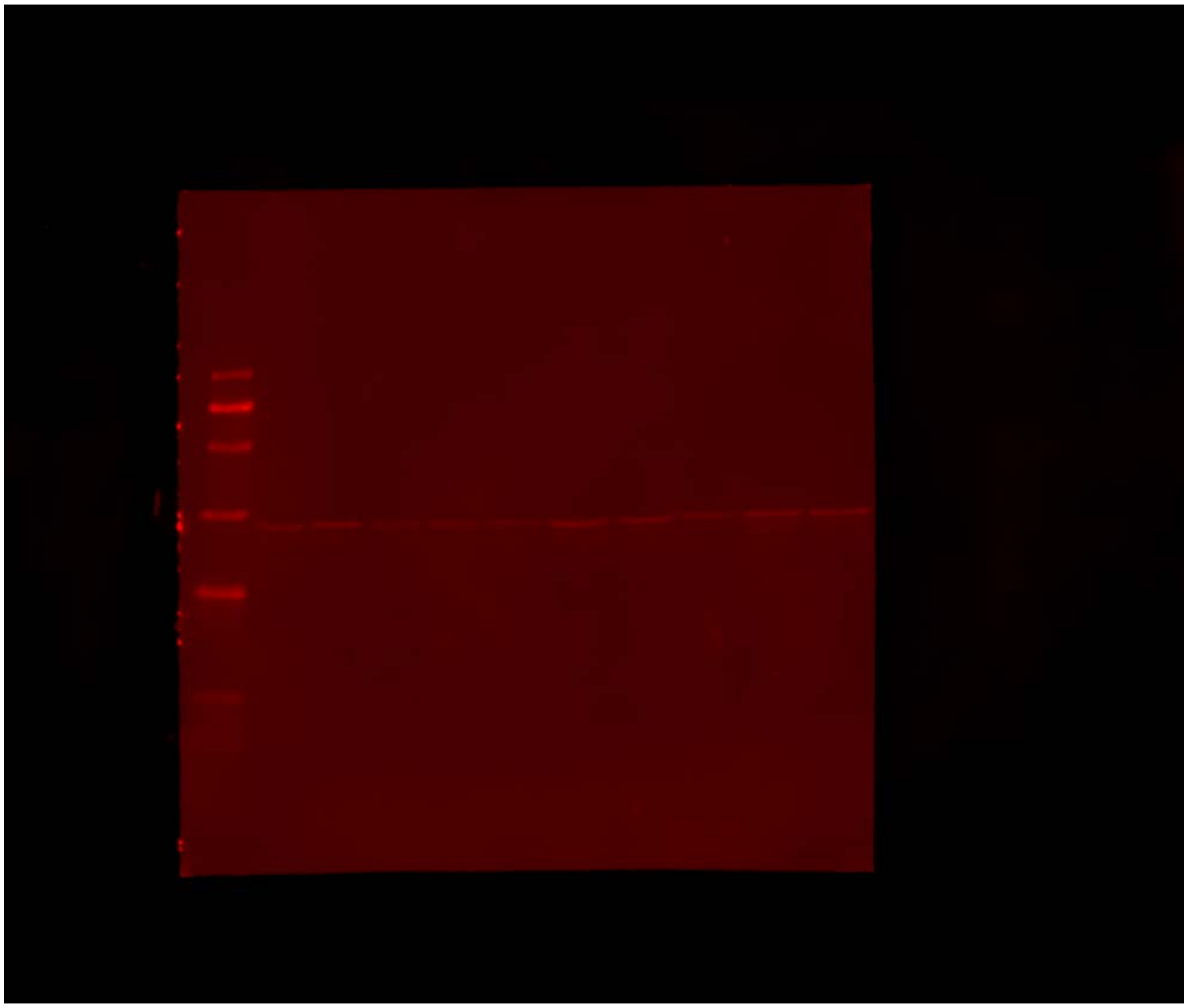

Total Protein

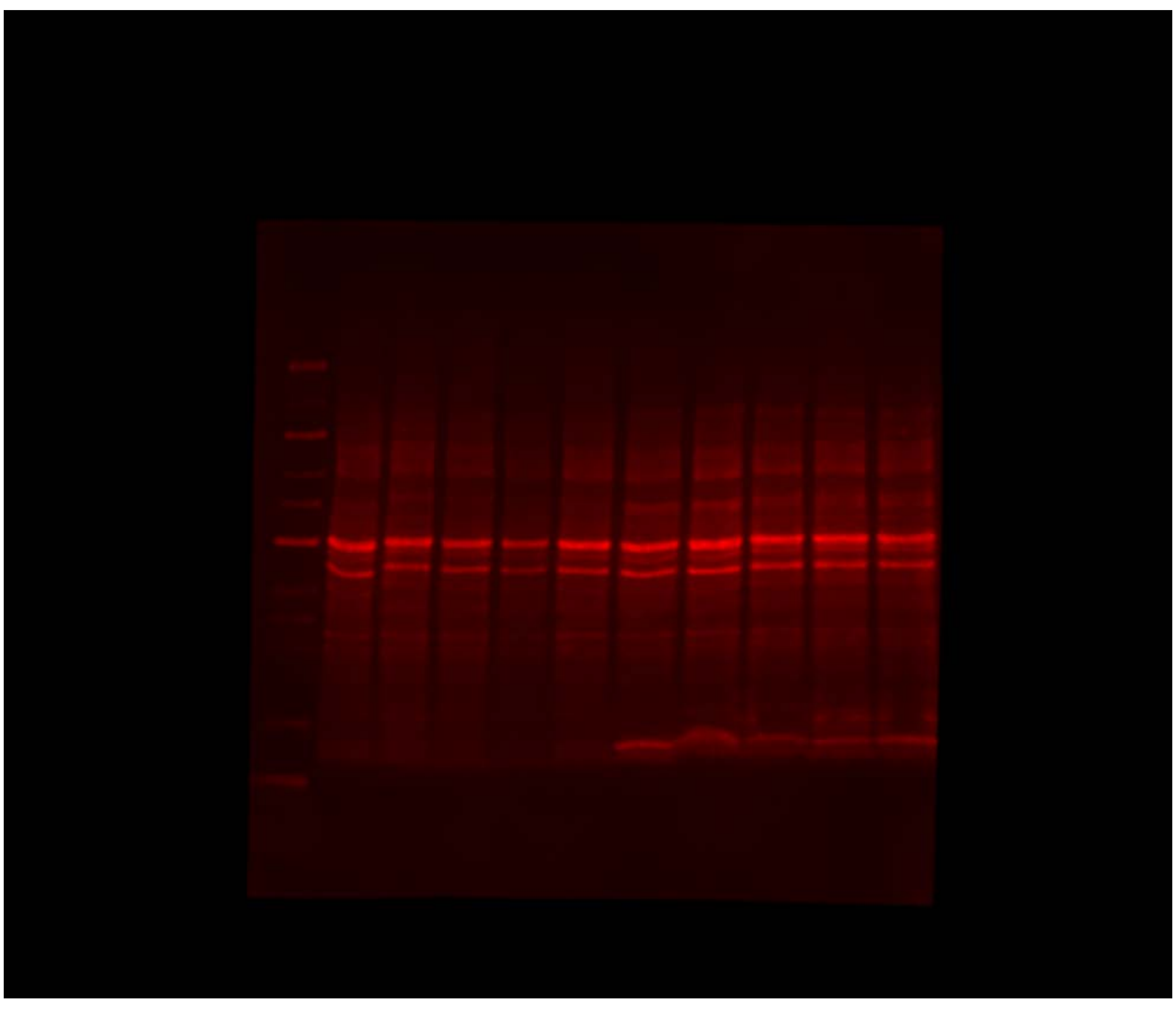

Supplement: Supplementary file 3 [file 41467_2026_77116_MOESM22_ESM.pdf]
