# Supplementary material for: Single-cell profiling identifies obesity-disrupted brain glucose sensing
Source: Nat Commun. 2026 Aug 31;17:10342. doi: 10.1038/s41467-026-77116-9 (PMC13623956; doi:10.1038/s41467-026-77116-9)
Supplement: Supplementary file 4 [file 41467_2026_77116_MOESM2_ESM.pdf]

nature portfolio

Peer Review File

Single-Cell Profiling Identifies Obesity-Disrupted Brain Glucose Sensing

Corresponding Author: Dr Tune Pers

This file contains all reviewer reports in order by version, followed by all author rebuttals in order by version.

Version 0:

Reviewer comments:

Reviewer #1

(Remarks to the Author)
This study presents a well-executed single-nucleus RNA-seq analysis of the arcuate/ventromedial hypothalamus and dorsal vagal complex in the context of acute hyperglycemia, shedding new light on how diet-induced obesity impairs central glucose sensing. By combining hyperglycemic clamps with immediate nuclei isolation, the authors effectively minimize batch effects and capture rapid transcriptional changes. In lean mice, they reveal a coordinated glial and neuronal response that likely underpins normal glycemic regulation, whereas this adaptive program was largely lost in obesity, which instead activates an unexpected dopaminergic population. Notably, markers of lean-state GHRH neurons are enriched for human glycemic GWAS loci, lending translational significance to the findings. Overall, the study compellingly shows that obesity disrupts a finely tuned glucose-sensing network and highlights GHRH neurons as a promising therapeutic target.

Functional validation of the key transcriptional findings is necessary. While the manuscript effectively maps obesity-induced transcriptional changes, it falls short in establishing causality. Incorporating targeted follow-up experiments—such as in situ hybridization for top marker genes, calcium imaging or electrophysiology to assess altered glucose responsiveness in GHRH and AgRP neurons, or chemogenetic gain- and loss-of-function approaches—would significantly strengthen the connection between the observed gene expression programs and corresponding changes in neuronal or glial function.

(Remarks on code availability)

Reviewer #2

(Remarks to the Author)
The authors employed snRNA-seq to identify transcriptional changes induced by hyperglycemia while comparing (1) lean mice treated with intravenous saline or glucose, and (2) diet-induced obese (DIO) mice treated with intravenous saline or glucose, all while focusing on cell-types within the ARC/VMH and the Hindbrain's Dorsal Vagal Complex (DVC). The study generated what appear to be the first snRNA-seq profiles in response to a glucose challenge in either the hypothalamus or hindbrain. It was observed that astrocytes and oligodendrocytes are the most responsive glial cells to DIO. Various neuronal types in the hypothalamus and hindbrain exhibited DIO-induced transcriptional changes, with Th/Slc6a3 neurons and AgRP neuron being the foremost transcriptionally responsive neuronal types. Glial and Neuronal population that are responsive to glucose were also identified, with Astrocytes in the hypothalamus, and Ghrh, AgRP, and Arx/Nr5a2 neurons being the most transcriptionally responsive. It was, via an immediate early gene panel, revealed that Ghrh neurons in lean mice are possibly silenced by glucose. In response to glucose, DIO mouse beta-tanycytes are the foremost responsive glial type, and Arx/Nr5a2, Th/Slc6a3, and AgRP neurons are the most transcriptionally responsive neuronal types. Notably, many of the transcriptional changes observed in response to glucose, in lean mice, were either abolished or inverted in DIO mice challenged with glucose. The authors posit that this represents evidence DIO confers a "metabolic inflexible state" that limits neuronal adaptations tied to the maintenance of glucose homeostasis; a novel and important conceptual framework for our field. Finally, the authors confirmed that a considerable number of mouse Ghrh neuron-specific genes are also human GWAS genes associated with glucose regulation.

While I have questions and comments related to the robustness of some of the transcriptional findings and other relatively

minor comments, the overarching trends and conceptual framework this manuscript offers warrant publication with only minor improvements.

My major comments are below:

1. The authors should explain why there is a discrepancy in terms of the number of HFD-changed genes, per neuronal type, in their study compared to what has been observed in Deng et al., 2020 paper (PMID: 32507042), and possibly Campbell et al., 2017, although mice in that study were only on a HFD for a few days. The Deng et al., 2020 paper observed an order of magnitude more genes (average of 418 across each of their cell clusters), compared to the tens of genes observed per cluster as being changed in this study, during the lean vs. DIO comparison. Also, the Deng et al., 2020 paper should be cited, and this apparent discrepancy should be addressed in the manuscript.

2. The body weight accrued during the 8 weeks of HFD was quite subtle. Is this truly a model of DIO or simply prolonged high-fat diet? Do these mice exhibit sufficient metabolic parameters to justify being called “obese”? Supplementary Figure 1c, I don’t see evidence of basal hyperinsulinemia. Moreover, the insulin infusion rate between lean and HFD mice was virtually indistinguishable, although the IS metric – which appears to largely be driven by plasma insulin levels rather than GIR – suggests some degree of insulin resistance. Was the intention to perform the experiment on pre-diabetic (these mice appear to be euglycemic), mildly insulin-resistant, mice? The authors should elaborate on their decision to use this particular model of hyperglycemia, and the decision to use, and the potential benefits of using, mice without overt obesity and signs of insulin resistance.

3. To assess the fidelity of their IEGs neuronal activation surrogate marker panel, authors should determine which neuronal types exhibit increased IEGs expression in response to DIO (saline conditions). Perhaps focusing on AgRP neurons, POMC neurons, and others. This might prove to be a powerful sanity check given the novelty of the continuous glucose infusion followed by snRNA-seq model. Essentially, before assuming this approach can accurately predict neuronal activity in this new system, it would be beneficial to demonstrate that this approach can accurately predict neuronal activity in a more common comparison where we can make predictions about the result.

4. It should be mentioned that a paper assessed c-fos to determine that neuronal and astrocyte populations across the hypothalamus respond to glucose (PMID: 15117340). This is important because it was shown that a marker of neuronal activity does up with glucose administration, which runs counter to your findings since you inferred activity measure did not detect a neuronal population exhibiting evidence of increased activity in the lean saline vs. glucose comparison.

5. Why do you think you don’t see evidence of inferred neuronal activation in response to glucose administration? Do you think that represents ground truth or is that finding owed to a limitation of your method for inferring neuronal activation, or possibly a consequence of your glucose administration protocol, including both the use of continuous infusion vs. a single bolus, and the time point used for tissue collection?

6. Show Supplementary Figures 3b and 4b in main Figure 2. If you need space, the schematic in Figure 2 is somewhat superfluous, and the subpanels in Figure 2b can be moved to the supplementary figure section.

7. Please explicitly list the number of nuclei that comprise each of your cellular subtypes. Is there a positive relationship between neuronal number of a cluster and the number of DEGs?

8. The heatmaps in Supplementary figure 7 should be in the main Figure 6.

9. Is it possible that you see the greatest response in astrocytes because, across brain regions, they are considerably homogenous (be it functionally or transcriptionally) compared to the neuronal populations in your dataset? And so, you're potentially underpowered in terms of the number of cells needed to appreciate robust transcriptional changes for your neuronal subtypes, but adequately powered for your astrocyte populations?

10. It's known that astrocytes can take up glucose, convert it to lactate, which can then be transported to neurons (i.e., astrocyte-neuron lactate shuttle). Do you see any genes that are implicated as being involved in lactate production or transport that are upregulated via acute glucose stimulation in astrocytes, or genes related to lactate transport and utilization in neurons?

11. For Ghrh, AgRP, and Arx/Nr5a2 neurons please show heatmaps revealing the IEG panel expression for all comparisons.

12. For the neuronal types that appear to show an expression panel that is inversely related between lean (glucose-induced) and DIO (glucose-induced), do you see a corresponding reversal of the IEG panel results, hinting at the transcriptional changes you see influencing neuronal activity?

Minor comments:

13. For Figure 6b (inferred activity) I don’t see a gray or red bar for the Arx/Nr5a2 population.

14. The authors intended to write Arx/Nr5a2 and not Arx/Nr5a1 in the figure 6 section of the results.

15. On pages 20-21 the authors wrote “We infer from this finding that DIO preconditions these neuronal populations to adopt

a transcriptional state in response to a glucose challenge that mirrors their chronic adaptation to obesity, with the consequence that the ability of these hypothalamic neurons to respond to hyperglycemia is blunted". I am confused by this statement. The graph appears to be comparing transcriptional changes in lean mice challenged with glucose to those changes that occur owed to DIO (saline). Transcriptional changes in DIO mice treated with glucose are not assessed in this figure. Should the word "obesity" here be replaced with hyperglycemia? If not, I'm not sure I follow.

16. The authors wrote: "Given the crucial role that the ARC/VMH plays in both energy and glucose homeostasis and to enhance marker gene identification, we integrated our single-cell data with an additional ARC/VMH dataset". Please cite the publication that generated the dataset you used here.

17. How novel is the finding that Arx/Slc6a3 neurons are exceptionally transcriptionally responsive to HFD? Should this be more heavily emphasized in the manuscript?

18. It is conceivable that the snRNA-seq detection method is only capturing a fragment of actual transcriptional changes, given the paucity of transcriptional changes observed in neuronal subtypes during DIO. Since the GWAS integration hits are limited to the snRNA-seq DEG list, if that list is artificially truncated due to possible limitations in the sensitivity of snRNA-seq, fewer GWAS hits will emerge. It seems Ghrh neurons are exceptionally responsive to changes in glucose, but it is unclear, based on the GWAS integration per se, whether it should be concluded that the transcriptional regulation of Pomc and Pnoc neurons does not play a major role in the ability of these neurons to influence glucose homeostasis.

19. It's not clear precisely how the Figure 6C correlation coefficient is calculated, this could be addressed in the figure legend, or even parenthetically beside the figure heading (Correlation coefficient).

(Remarks on code availability)
NA

Reviewer #3

(Remarks to the Author)
This work by Dr. Brown is a massive bioinformatics efforts to tease out transcriptional differences between control and obese mice, and then to compare (differential) responsiveness to 1-h hyperglycaemia. The study is well-performed, and the paper has all the by now standard features of high-quality output from the Pers group. As such, both the spatial (ARC vs. DVC), cell-type-specific, and "function-predicting" comparisons that had been made will be useful to the scientific community. While I am most supportive of the paper be published, I am of the view that the authors must perform critical experiments to both justify the biological significance of their data, and to prevent others from "spin-doctoring" their findings.

Major concerns:
1. Experimental design. The study has two inherent problems:
ad 1) the period of 4h fasting prior to hyperglycaemia. Back in 2004, Tamas Horvath's group published rapid synaptic rewiring upon fasting, and leptin exposure, i.e. the removal of GABA and glutamate afferents from Agrp/Pomc neurons and their temporary replacement with astrocyte end-feet, to allow for the plastic remodelling of excitatory/inhibitory synapse populations. The authors find plenty of axon guidance molecules, RhoGTPases, Ras etc. in astrocytes, and changes in GABA receptors. These all could suggest that the authors indeed observe (even if inadvertently) plastic remodelling in the ARC, perhaps even involving Gnrh-to-Agrp synapses. This is a testable hypothesis that minimally needs quantitative anatomy.
ad 2) 1-h hyperglycaemia: this is a very short period of time. Transcriptional waves often need much longer periods to manifest, and most importantly, stabilise. How can the authors argue that most of the changes they see (beyond immediate early and glucose-sensing-related genes) have anything to do with brain adaptation (and not just stochastic). It would be most impressive to see a later (e.g., 4h or 24h) time-point to resolve if any change becomes stable (or a "susceptibility gene set" emerges supporting and linking the experimental and human GWAS datasets).

2. General physiology: There is not a single biological benchmarking attempt to seriously advance physiology/metabolism research. This is a major caveat. Minimally, transcriptional changes for astrocytes, oligodendrocytes or neurons shall be justified by in situ hybridisation/histochemistry (e.g., "reactive gliosis" is not equivalent to an increase in GFAP expression but also a change in astrocyte morphology and positioning). Why not testing tight junction coupling by antibodies? Why not testing Gnrh cell input to Agrp neurons? If the authors are correct, Gnrh input shall decrease, which would allow by disinhibition to increase Agrp AP spiking/output.

3. Use of GO and other classifiers: The Authors use classifiers in a very confusing fashion. What is the difference between e.g., „neurotransmitter regulation“ vs. „synaptic regulation“? Why are these terms coming up, along with axon guidance molecules, in glial cells? Why are Slit2 and Robo classified differently although they are a ligand-receptor pair. A very prudent and didactical approach needs to be made to not just crudely sum pathways that could be very misleading for follow-up studies.

4. Interpretations: These are excessive in the results. Interpretations are starting points to experiments. The authors cannot argue for "later research" every time. Then, the work shall go to a more bioinformatics-minded journal. Please clear up the results such that it is to the point, and does not rely on excessive argumentation (which could equally be false) to the data.

More minor:
Line 73: can one talk or imply 'inhibition' when looking at RNA-seq data.
Lines 109-110 and 141-142: sentences seems fragmented.
Line 183: word missing after Agrp
Fig. 2: please split this into two figures to separate astrocytes and neurons. It would be helpful for Fig. 2f to have background „clouds" to encircle GABA vs. glutamate clusters because the many genes does not help the classifier. Conversely, DVC neurons are all „GABA" and „glutamate" subtypes but not specified by gene sets. This could be/should be resolved by adding in genes and encircle the neurotransmitter phenotypes instead.
Lines 178-179: „four GABA cell populations" Th/Slc6a3 are likely TIDA cells. Why would these be GABAergic and not dopaminergic? Do the authors refer to developmental origins?
Line 224: why have oligodendrocytes to do something with 'reduction of synaptic structural components'? This makes little sense even if GO ontology is used given that it is neuronal structures that should change. How can this be reconciled? 'Physiolgoical adaptation' needs to be addressed otherwise this section is meaningless.
Line 250: the statement means the cells that responded most are NOT within the DVC. Is this correct? If so, should not these cells be excluded from the analysis anyway?

(Remarks on code availability)

Version 1:

Reviewer comments:

Reviewer #2

(Remarks to the Author)
The authors meticulously responded to each of my comments, while incorporating additional analyses, updated figure layouts, and text edits, which greatly improved the interpretability of their findings. Whereas I previously recommended this manuscript for publication, I now strongly recommend this manuscript be published in its current form.

(Remarks on code availability)

Reviewer #3

(Remarks to the Author)
The reviewer thanks the Authors for returning this revision, and their extensive rebuttal. The paper certainly retains significant interest even if it being a "useful dataset" for follow-up by others is a major turn-off and undersells the study.

Unfortunately, the major issue independently spotted by all referees remains open, it being the lack of biological validation. This is most unexpected for experimentally-versed Authors with significant resources.

Whilst this reviewer would wish this paper be published here, it cannot be without validation. The neuroscience community has seen so many misleading studies: what about all the lost investment, potentially career-ending mishaps, if any of the authors' interpretations were incorrect yet followed up. Who is to blame then?

Beyond the "moral obligation", the Authors could have performed simple experiments: neuroanatomy by light microscopy (i.e., dual or triple labelling). In situ hybridisation by HCR or similar "out of the box" kit. The GHRH connectome could have been reconstructed using an AAV delivering GFP/mCherry and combined with a Fos or other gene from the DEG set.

This reviewer will not change their view even at a later stage: every bioinformatics study should be backed up by biology. Otherwise, the dataset could be made public on GEO with no strings attached for anyone to mine the data. At the same time, this reviewer would caution that it is customary to disclose reviews/rebuttals with published papers in full. Thus, Figure 1 could well become "published material" through that route (the data are fantastic yet collateral/dispensable to this particular study).

Lastly, and even if this had not been part of the original review (the discussion with Referee 2 was quite interesting): Arx is critical for the development, specification, and maintenance of pancreatic alpha cells. Thus, Arx-expressing neurons being foremost to glucose manipulation could be contextualised across tissues and organs.

(Remarks on code availability)
N/A

Version 2:

Reviewer comments:

Reviewer #3

(Remarks to the Author)
I appreciate the authors' renewed efforts to provide biological confirmation/support for some of their findings. While I am content with the experimentation itself, I suggest to better integrate the new neuroanatomy data and move them from the supporting figures to the main ones to increase visibility. I also think the authors shall highlight the minimal experimental follow-up as a limitation already in the abstract. I have no questions beyond this limited reorganisation.

(Remarks on code availability)
N/A

Open Access This Peer Review File is licensed under a Creative Commons Attribution 4.0 International License, which permits use, sharing, adaptation, distribution and reproduction in any medium or format, as long as you give appropriate credit to the original author(s) and the source, provide a link to the Creative Commons license, and indicate if changes were made.
In cases where reviewers are anonymous, credit should be given to 'Anonymous Referee' and the source.
The images or other third party material in this Peer Review File are included in the article's Creative Commons license, unless indicated otherwise in a credit line to the material. If material is not included in the article's Creative Commons license and your intended use is not permitted by statutory regulation or exceeds the permitted use, you will need to obtain permission directly from the copyright holder.
To view a copy of this license, visit https://creativecommons.org/licenses/by/4.0/

POINT-BY-POINT RESPONSE TO THE REVIEWER COMMENTS

We would like to thank the reviewers for their helpful comments. We found them to be thoughtful and constructive, and they helped us improve our manuscript considerably.

Reviewer #1 (Remarks to the Author):

This study presents a well-executed single-nucleus RNA-seq analysis of the arcuate/ventromedial hypothalamus and dorsal vagal complex in the context of acute hyperglycemia, shedding new light on how diet-induced obesity impairs central glucose sensing. By combining hyperglycemic clamps with immediate nuclei isolation, the authors effectively minimize batch effects and capture rapid transcriptional changes. In lean mice, they reveal a coordinated glial and neuronal response that likely underpins normal glycemic regulation, whereas this adaptive program was largely lost in obesity, which instead activates an unexpected dopaminergic population. Notably, markers of lean-state GHRH neurons are enriched for human glycemic GWAS loci, lending translational significance to the findings. Overall, the study compellingly shows that obesity disrupts a finely tuned glucose-sensing network and highlights GHRH neurons as a promising therapeutic target.

Functional validation of the key transcriptional findings is necessary. While the manuscript effectively maps obesity-induced transcriptional changes, it falls short in establishing causality. Incorporating targeted follow-up experiments—such as in situ hybridization for top marker genes, calcium imaging or electrophysiology to assess altered glucose responsiveness in GHRH and AgRP neurons, or chemogenetic gain- and loss-of-function approaches—would significantly strengthen the connection between the observed gene expression programs and corresponding changes in neuronal or glial function.

Response: We thank the reviewer for their insightful comments and appreciate the reviewer's emphasis on the importance of validating the transcriptional findings with complementary approaches. Multiple independent studies have established that glucose inhibits both AgRP and GHRH neurons (e.g., Fioramonti et al. Diabetes 2007; Stanley et al. Cell Metabolism 2013), providing functional context for our transcriptomic observations. Studies by Drs. Alhedeff, Beutler and others have also clearly shown that in AgRP-cre mice, AgRP neuron activity is rapidly and robustly inhibited following glucose administration, not only in response to oral glucose delivery but also following glucose infusion into the hepatic portal vein. As detailed below, unpublished work by one of us (Schwartz lab) has extended this work by examining the temporal relationship between changes of glycemia and AgRP neuron activity following either oral or iv glucose administration.

Also relevant is our recent finding (reported in Gou et al JCI 2025) that in the $Lep^{ob/ob}$ mouse model of type 2 diabetes, AgRP neuron inactivation normalizes elevated blood glucose levels. As AgRP neurons are hyperactive in these mice at baseline (and most other models of diabetes), this finding clearly implicates hyperactivity of these neurons as drivers of hyperglycemia in this model, adding additional context within which to interpret the observed transcriptional patterns.

To further strengthen this connection, we included in this point-by-point response unpublished data from the Schwartz lab (**Figure 1**). Their experimental approach combined continuous arterial blood glucose monitoring with fiber photometry in AgRP-Cre mice to analyze the temporal dynamics following glucose administration. These data show that in conscious free-living mice, the duration of AgRP neuron inhibition directly scales with the degree of glycemia increase across multiple doses of oral glucose, consistent with our transcriptional findings. Although the contribution of so-called ‘gut-brain’ signals vs. changing blood glucose dynamics to the AgRP neuron response awaits further study, these data clearly show that hyperglycemia associates significantly with AgRP neuron inhibition in vivo. We hope the reviewer will agree that although more work is needed, this information provides a functional context within which to consider hyperglycemia-induced gene expression programs.

These unpublished studies were conducted by an entirely different investigative team, involving primarily oral rather than iv glucose, and a manuscript describing this work is already in process. For these reasons, we do not feel it appropriate to include the data in the current paper. We hope instead that our revisions highlighting published evidence that the transcriptionally responsive AgRP and GHRH neurons are inhibited by glucose (see page 16) will suffice to address the reviewer’s concern.

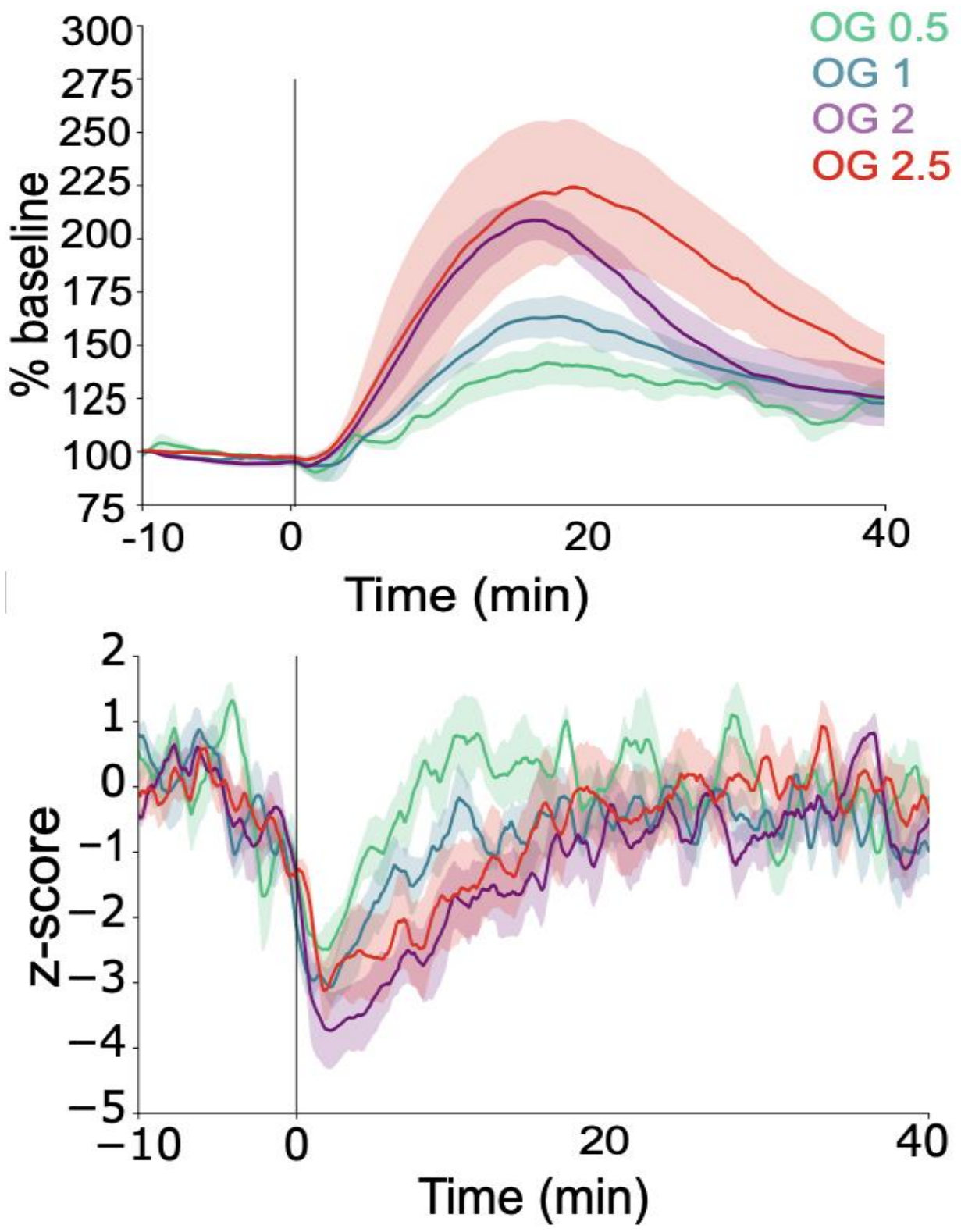

Figure 1: Arterial blood glucose levels (upper panel) and AgRP neuron activity (lower panel) were monitored continuously by continuous glucose monitoring (CGM) and fiber photometry, respectively, in response to oral gavage of one of 4 doses of glucose (0.5, 1.0. 2.0 or 2.5 grams). The studies were performed in adult male AgRP-cre mice fasted for 5 h before glucose was administered. AgRP neuron activity is rapidly inhibited following each glucose dose, with the duration of the inhibitory response varying with the degree of hyperglycemia.

Reviewer #2 (Remarks to the Author):

The authors employed snRNA-seq to identify transcriptional changes induced by hyperglycemia while comparing (1) lean mice treated with intravenous saline or glucose, and (2) diet-induced obese (DIO) mice treated with intravenous saline or glucose, all while focusing on cell-types within the ARC/VMH and the Hindbrain’s Dorsal Vagal Complex (DVC). The study generated what appear to be the first snRNA-seq profiles in response to a

glucose challenge in either the hypothalamus or hindbrain. It was observed that astrocytes and oligodendrocytes are the most responsive glial cells to DIO. Various neuronal types in the hypothalamus and hindbrain exhibited DIO-induced transcriptional changes, with Th/Slc6a3 neurons and AgRP neuron being the foremost transcriptionally responsive neuronal types. Glial and Neuronal population that are responsive to glucose were also identified, with Astrocytes in the hypothalamus, and Ghrh, AgRP, and Arx/Nr5a2 neurons being the most transcriptionally responsive. It was, via an immediate early gene panel, revealed that Ghrh neurons in lean mice are possibly silenced by glucose. In response to glucose, DIO mouse beta-tanycytes are the foremost responsive glial type, and Arx/Nr5a2, Th/Slc6a3, and AgRP neurons are the most transcriptionally responsive neuronal types. Notably, many of the transcriptional changes observed in response to glucose, in lean mice, were either abolished or inverted in DIO mice challenged with glucose. The authors posit that this represents evidence DIO confers a "metabolic inflexible state" that limits neuronal adaptations tied to the maintenance of glucose homeostasis; a novel and important conceptual framework for our field. Finally, the authors confirmed that a considerable number of mouse Ghrh neuron-specific genes are also human GWAS genes associated with glucose regulation.

While I have questions and comments related to the robustness of some of the transcriptional findings and other relatively minor comments, the overarching trends and conceptual framework this manuscript offers warrant publication with only minor improvements.

My major comments are below:

1. The authors should explain why there is a discrepancy in terms of the number of HFD-changed genes, per neuronal type, in their study compared to what has been observed in Deng et al., 2020 paper (PMID: 32507042), and possibly Campbell et al., 2017, although mice in that study were only on a HFD for a few days. The Deng et al., 2020 paper observed an order of magnitude more genes (average of 418 across each of their cell clusters), compared to the tens of genes observed per cluster as being changed in this study, during the lean vs. DIO comparison. Also, the Deng et al., 2020 paper should be cited, and this apparent discrepancy should be addressed in the manuscript.

Response: We thank the reviewer for highlighting this important point. We recognize that the number of differentially expressed genes (DEGs) per neuronal cluster observed in our study is lower than that reported by Deng et al. (Hypertension 2020). This discrepancy can be attributed to several key experimental and analytical differences. While both studies achieved similar degrees of weight gain in mice, Deng et al. used a 45% high-fat diet compared to normal chow, whereas our study employed a 60% high-fat diet compared to a matched control diet, which accounts for the highly processed nature of the diet.

Importantly, our analytical pipeline also applies more conservative and statistically appropriate criteria. Differentially expressed genes should be calculated in a manner that accounts for the non-independence of cells originating from the same donor, because single nuclei from one mouse are not statistically independent observations. To address this, we performed DEG analysis using a pseudobulk approach, in which all nuclei from a given cluster are aggregated per animal. This method directly controls for donor-level effects and aligns with best practices recommended in the field, including pseudobulking or the use of tools such as muscat (Crowell et al., Nat. Commun. 2020). Additionally, we used a stricter FDR threshold (FDR < 0.05) than

Deng et al. (FDR < 0.1), which also contributed to identifying fewer DEGs. We have now cited both Deng et al., 2020 and Campbell et al., 2017 in the revised manuscript and explicitly discuss these methodological distinctions in the Discussion section:

"Moreover, in the HFD to control diet comparison, we identified a smaller number of differentially expressed genes relative to Dreng et al [24] and Campbell et al [25], which most likely is due to our more conservative significance threshold (FDR<0.05 vs. 0.1) and pseudobulk-based testing to account for donor-level dependence."

2. The body weight accrued during the 8 weeks of HFD was quite subtle. Is this truly a model of DIO or simply prolonged high-fat diet? Do these mice exhibit sufficient metabolic parameters to justify being called "obese"? Supplementary Figure 1c, I don't see evidence of basal hyperinsulinemia. Moreover, the insulin infusion rate between lean and HFD mice was virtually indistinguishable, although the IS metric – which appears to largely be driven by plasma insulin levels rather than GIR – suggests some degree of insulin resistance. Was the intention to perform the experiment on pre-diabetic (these mice appear to be euglycemic), mildly insulin-resistant, mice? The authors should elaborate on their decision to use this particular model of hyperglycemia, and the decision to use, and the potential benefits of using, mice without overt obesity and signs of insulin resistance.

Response: We appreciate the reviewer's careful consideration of our experimental model. We agree that the mice used here are more accurately described as 'high-fat-diet (HFD) fed" rather than fully diet-induced-obese (DIO). We have therefore revised the text to consistently use "HFD-fed" throughout the manuscript and to clarify our rationale for employing this model.

We also clarify that our goal was not to model advanced obesity per se, but to investigate how chronic high-fat-diet exposure alters central glucose-sensing mechanisms before the onset of severe obesity or diabetes. After eight weeks of HFD feeding, the animals displayed modest weight gain but showed higher plasma insulin levels than chow-fed controls during the glucose infusion (Supplementary Fig. 1C), indicating an early stage of insulin resistance. This physiological profile is well suited for probing how diet exposure, independent of increased adiposity, affects hypothalamic and hindbrain transcriptional responses to acute hyperglycemia (see page 6 and forward).

An important clarification is that the glucose-infusion rate used to maintain target glycemia during the hyperglycemic challenge is not a reliable measure of insulin sensitivity, since insulin concentrations were not "clamped" (or matched) between groups. This approach stands in contrast to information gained from the glucose infusion rate during a euglycemic, hyperinsulinemic clamp. The latter is a valid measure of insulin sensitivity because it reflects the rate at which glucose leaves the blood stream in response to hyperinsulinemia – information that cannot be inferred from the rate of glucose infusion needed to induce stable hyperglycemia.

Finally, we note in the Discussion that the modest weight differences actually strengthen our interpretation, as the observed transcriptional changes cannot be attributed to potentially confounding effects of large variations in fat mass. Thus, the study isolates the impact of HFD exposure on central glucose sensing without the

potentially confounding effects of obesity and its sequelae. We have added to the Discussion:

"Strikingly, HFD inverted these hyperglycemia-induced transcriptional signatures, indicating that even modest weight gain causes a fundamental shift in how these brain regions sense and respond to an elevated blood glucose level."

3. To assess the fidelity of their IEGs neuronal activation surrogate marker panel, authors should determine which neuronal types exhibit increased IEGs expression in response to DIO (saline conditions). Perhaps focusing on AgRP neurons, POMC neurons, and others. This might prove to be a powerful sanity check given the novelty of the continuous glucose infusion followed by snRNA-seq model. Essentially, before assuming this approach can accurately predict neuronal activity in this new system, it would be beneficial to demonstrate that this approach can accurately predict neuronal activity in a more common comparison where we can make predictions about the result.

Response: We appreciate the reviewer's question regarding the validity of our inferred neuronal activation surrogate. As correctly noted, single-nucleus RNA-seq is not designed to capture rapid or transient electrical activity and can only assess transcriptional correlates of neuronal activation at a single time point. Our use of immediate early gene (IEG) expression as a surrogate follows established precedent (e.g., Hrvatin et al., 2018; Wu et al., 2021; Simon et al., 2024), where IEG-based scores are used to infer neuronal activation states in single-cell and single-nucleus datasets.

We have clarified in the revised manuscript that this strategy provides a qualitative indication of activation patterns, not a quantitative measure of neuronal firing. The Discussion now explicitly notes that electrophysiological or calcium-imaging methods would be required to directly assess real-time activity, whereas the transcriptomic approach provides complementary insights into gene expression programs that accompany such changes measured at a single time point:

"Future studies may also i) use in vivo calcium imaging or electrophysiology to validate the suggestive neuron activity changes in Ghrh and Agrp neurons observed at the transcriptional level [...]"

4. It should be mentioned that a paper assessed c-fos to determine that neuronal and astrocyte populations across the hypothalamus respond to glucose (PMID: 15117340). This is important because it was shown that a marker of neuronal activity does up with glucose administration, which runs counter to your findings since you inferred activity measure did not detect a neuronal population exhibiting evidence of increased activity in the lean saline vs. glucose comparison.

Response: We appreciate the reviewer's interest in why we did not observe widespread neuronal activation signatures similar to those reported in prior glucose infusion studies. We believe this discrepancy reflects both methodological and physiological differences.

First, our experimental design employed a continuous intravenous infusion to 'clamp' plasma glucose at a fixed value (~300 mg/dL) for one hour. This sustained and

modest hyperglycemia differs fundamentally from the acute, non-physiological stimulus provided by a carotid glucose bolus referenced by the reviewer (e.g., PMID: 15117340). The latter can be expected to raise local blood and brain glucose levels to values higher than were achieved in our study, but in a transient up-and-down manner, such that the glucose stimulus is continuously changing rather than held constant. It seems reasonable to anticipate differences in the transcriptional response in the face of a markedly different glucose profile.

Second, our team's independent fiber-photometry data show that AgRP neuron inhibition tracks closely with the rate of change of circulating glucose rather than its absolute level (not shown). Under a continuous glucose-infusion paradigm, the rate of change is effectively zero once stable hyperglycemia is achieved, potentially explaining why single-nucleus RNA-seq at a 60-minute time point did not reveal robust activity-associated transcriptional changes in AgRP neurons.

We have expanded the Discussion to include these points and to emphasize that our study aimed to characterize transcriptional responses to stable hyperglycemia across neurons and glia, rather than to elicit maximal neuronal activation, while avoiding confounding effects of a non-physiologic glucose surge:

"We acknowledge that earlier studies using acute, high-dose carotid glucose boluses showed increased c-fos expression across hypothalamic cell types, but this stimulus produces rapid and transient glucose fluctuations that differ markedly from the stable hyperglycemia induced in our glucose-clamp paradigm. Because neuronal activation markers such as c-fos respond strongly to changes in glucose rather than steady levels, our design would not be expected to replicate these earlier findings. Thus, the lack of widespread neuronal activation signatures in our dataset is consistent with the distinct experimental conditions and physiological responses involved."

5. Why do you think you don't see evidence of inferred neuronal activation in response to glucose administration? Do you think that represents ground truth or is that finding owed to a limitation of your method for inferring neuronal activation, or possibly a consequence of your glucose administration protocol, including both the use of continuous infusion vs. a single bolus, and the time point used for tissue collection?

Response: Please see our response to Comment 4, above.

6. Show Supplementary Figures 3b and 4b in main Figure 2. If you need space, the schematic in Figure 2 is somewhat superfluous, and the subpanels in Figure 2b can be moved to the supplementary figure section.

Response: We agree that these data are important for the reader and have moved the relevant panels from Supplementary Figures 3b and 4b into the main Figure 2. To accommodate this change, we split Figure 2 into two figures as suggested by Reviewer 3.

7. Please explicitly list the number of nuclei that comprise each of your cellular subtypes. Is there a positive relationship between neuronal number of a cluster and the number of DEGs?

Response: We thank the reviewer for this suggestion. The number of nuclei contributing to each cellular subtype is now listed in Supplementary Table S1. We also examined whether the number of differentially expressed genes correlated with cluster size and found no positive relationship, indicating that the observed DEG patterns are not driven by differences in the number of nuclei.

8. The heatmaps in Supplementary figure 7 should be in the main Figure 6.

Response: We agree that these heatmaps enhance interpretation and have moved them from the Supplementary section to the main Figure 6, as suggested.

9. Is it possible that you see the greatest response in astrocytes because, across brain regions, they are considerably homogenous (be it functionally or transcriptionally) compared to the neuronal populations in your dataset? And so, you're potentially underpowered in terms of the number of cells needed to appreciate robust transcriptional changes for your neuronal subtypes, but adequately powered for your astrocyte populations?

Response: We appreciate this insightful point and agree that statistical power to detect differentially expressed genes depends on cell numbers within each cluster. However, we believe the stronger astrocyte response is unlikely to reflect underpowered neuronal sampling. In control diet animals, ARC/VMH and DVC astrocyte populations contain similar cell numbers, yet the ARC/VMH shows a considerably stronger transcriptional response to glucose (5587 vs 5425 DEGs; Supplementary Figure 5). Furthermore, HFD astrocytes in the DVC show no differentially expressed genes despite comparable cell numbers. These observations suggest that cell number alone does not drive the observed responses. We acknowledge that larger neuronal populations would have increased our power to detect additional differentially expressed genes, and we have added Supplementary Table 3 providing cell numbers across all populations.

10. It's known that astrocytes can take up glucose, convert it to lactate, which can then be transported to neurons (i.e., astrocyte-neuron lactate shuttle). Do you see any genes that are implicated as being involved in lactate production or transport that are upregulated via acute glucose stimulation in astrocytes, or genes related to lactate transport and utilization in neurons?

Response: We thank the reviewer for raising this mechanistic question. As noted above, the astrocyte–neuron lactate shuttle hypothesis posits that astrocytes metabolize glucose to lactate, which is then transported to neurons via monocarboxylate transporters for oxidative metabolism. While we examined expression of key genes associated with this pathway (e.g., *Ldha, Ldhb, Slc16a1* [*MCT1*], *Slc16a3* [*MCT4*]), we did not detect consistent upregulation of these transcripts in astrocytes in response to acute hyperglycemia (see below **Figure 2**). We note, however, that the function of this shuttle does not necessarily require dynamic transcriptional changes, as these enzymes and transporters are constitutively expressed.

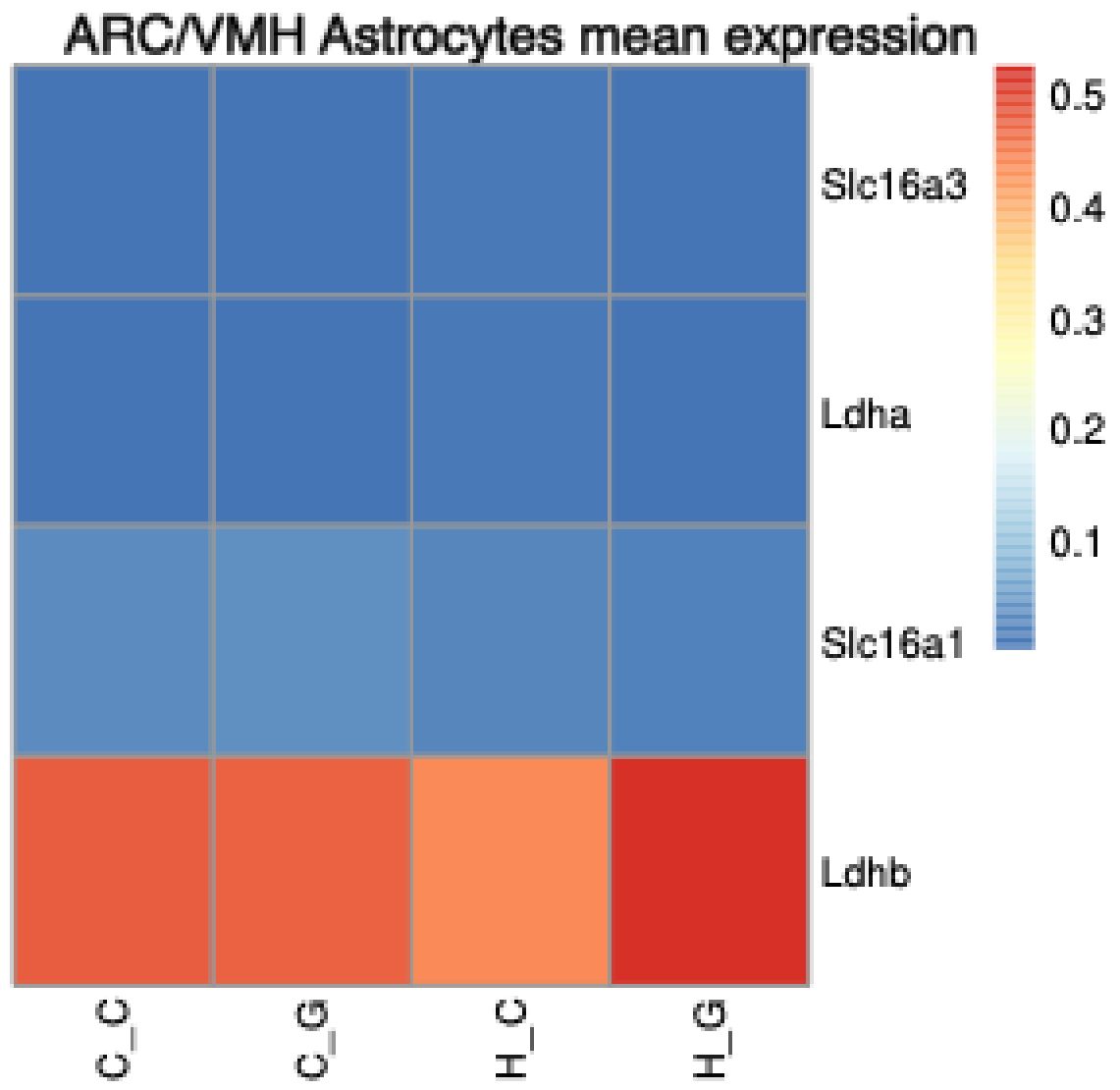

Figure 2: Expression of astrocyte-neuron lactate shuttle (*Ldha, Ldhb, Slc16a1*, *Slc16a3*) in ARC/VMH astrocytes (mean expression per cluster). None of the genes are regulated by any of the treatments (C_C, control diet and saline; C_G, control diet and glucose; H_C, HFD and saline; H_G, HFD and glucose).

11. For Ghrh, AgRP, and Arx/Nr5a2 neurons please show heatmaps revealing the IEG panel expression for all comparisons.

Response: We thank the reviewer for this helpful suggestion. We have now added heatmaps displaying the IEG panel expression for Ghrh, AgRP, and Th/Slc683 neuronal populations across all relevant experimental comparisons (see new Supplementary Figure S6). Arx/Nr5a2 neurons are not included because the expression of these genes are zero or they are not captured. These visualizations more clearly illustrate the relative activation states and inferred from our IEG-based approach.

12. For the neuronal types that appear to show an expression panel that is inversely related between lean (glucose-induced) and DIO (glucose-induced), do you see a corresponding reversal of the IEG panel results, hinting at the transcriptional changes you see influencing neuronal activity?

Response: We appreciate the reviewer's insightful question. Indeed, while global IEG changes across all neuronal populations were not statistically significant after multiple-testing correction, certain cell types exhibited consistent and biologically meaningful trends. Specifically, glucose administration in chow-fed mice led to a clear reduction in G expression in GHRH neurons, consistent with glucose-induced neuronal inhibition (Results Figure 5). However, this effect was abolished following HFD exposure, such that glucose no longer suppressed IEG expression in these neurons (Results Figure 6d and Supplemental Figure 6a). This pattern mirrors the transcriptional reversal observed in GHRH neurons between the lean and HFD-fed states and supports our interpretation that diet exposure disrupts normal glucose-sensing mechanisms.

Minor comments:

13. For Figure 6b (inferred activity) I don't see a gray or red bar for the Arx/Nr5a2 population.

Response: We thank the reviewer for noting this omission. We have adjusted the manuscript to reflect the inferred activity based on the mean expression and inferred activity in Supplementary Figure S6 and only included cell populations we reference in the text.

14. The authors intended to write Arx/Nr5a2 and not Arx/Nr5a1 in the figure 6 section of the results.

Response: We appreciate the reviewer catching this typographical error. All references to Arx/Nr5a1 have been corrected to Arx/Nr5a2 throughout the text.

15. On pages 20-21 the authors wrote "We infer from this finding that DIO preconditions these neuronal populations to adopt a transcriptional state in response to a glucose challenge that mirrors their chronic adaptation to obesity, with the consequence that the ability of these hypothalamic neurons to respond to hyperglycemia is blunted". I am confused by this statement. The graph appears to be comparing transcriptional changes in lean mice challenged with glucose to those changes that occur owed to DIO (saline). Transcriptional changes in DIO mice treated with glucose are not assessed in this figure. Should the word "obesity" here be replaced with hyperglycemia? If not, I'm not sure I follow.

Response: The sentence on pages 20–21 has been rephrased to clarify that exposure to a high-fat diet preconditions neuronal populations to adopt transcriptional states resembling those induced by chronic nutrient excess, thereby blunting a further acute response to hyperglycemia. We appreciate the reviewer's suggestion, which improves precision and readability. The updated sentence reads:

"We infer from this finding that exposure to a HFD preconditions neuronal populations to adopt transcriptional states resembling those induced by chronic nutrient excess, thereby blunting a further acute response to hyperglycemia. "

16. The authors wrote: "Given the crucial role that the ARC/VMH plays in both energy and glucose homeostasis and to enhance marker gene identification, we integrated our single-cell data with an additional ARC/VMH dataset". Please cite the publication that generated the dataset you used here.

Response: We agree and are currently uploading the data from which the ARC/VMH single-cell dataset was obtained to GEO and will update the citation to these data in the Methods section upon acceptance of this manuscript.

17. How novel is the finding that Arx/Slc6a3 neurons are exceptionally transcriptionally responsive to HFD? Should this be more heavily emphasized in the manuscript?

Response: We have added text in the results that addresses the transcriptional response observed in the Th/Slc6a3 neuronal population. This population has been described in previous hypothalamic scRNA-seq studies (Campbell et al., 2017) and our data provide additional insight into its transcriptional dynamics under high-fat-diet conditions. While interesting, we present this as an observation rather than a major novel finding, acknowledging that further work will be needed to determine the physiological significance of these changes. In the Results section on page 10 we now write:

“Two of these populations (Th/Slc6a3 and Agrp neurons) have previously been shown to be transcriptionally regulated by HFD feeding and fasting [24-26].”

18. It is conceivable that the snRNA-seq detection method is only capturing a fragment of actual transcriptional changes, given the paucity of transcriptional changes observed in neuronal subtypes during DIO. Since the GWAS integration hits are limited to the snRNA-seq DEG list, if that list is artificially truncated due to possible limitations in the sensitivity of snRNA-seq, fewer GWAS hits will emerge. It seems Ghrh neurons are exceptionally responsive to changes in glucose, but it is unclear, based on the GWAS integration per se, whether it should be concluded that the transcriptional regulation of Pomc and Pnoc neurons does not play a major role in the ability of these neurons to influence glucose homeostasis.

Response: The GWAS enrichment analysis was done using the CELLECT tool (Timshel et al., eLife, 2020) and did not depend on the number of snRNA-seq DEGs. However, we acknowledge that the number of glycemic GWAS loci detected depends on the depth and sensitivity of the snRNA-seq dataset. While our current data identify significant enrichment of glycemic-trait GWAS genes within GHRH neuronal clusters, we cannot exclude the possibility that other populations such as Pomc and Pnoc neurons also harbor relevant genes that fell below detection thresholds. What should also be noted is the importance of GWAS sample size. Larger GWAS studies typically identify more loci, which could potentially reveal additional cell-type associations not captured in our analysis. We have added text to the Discussion to note these limitations and to emphasize that the observed enrichment in GHRH neurons supports a strong, but not exclusive, link between these neurons and human glycemic regulation:

“Potential explanations are that the potential glucose sensing function of these neurons is not under genetic control, that current GWAS are not well-powered enough to implicate such genetic variants, or that their contribution becomes more apparent under different physiological or developmental conditions.”

19. It's not clear precisely how the Figure 6C correlation coefficient is calculated, this could be addressed in the figure legend, or even parenthetically beside the figure heading (Correlation coefficient).

Response: We thank the reviewer for this request. We now specify in the figure legend and Methods that the correlation coefficient in Figure 6c was calculated using a Kendall correlation, based on the correlation of average gene-expression fold changes between the experimental conditions lean (glucose-induced) and DIO (glucose-induced) across matched neuronal subtypes.

Reviewer #2 (Remarks on code availability):

NA

Summary: We thank Reviewer #2 for their extensive and highly constructive comments. Receiving such thorough and, importantly, constructive feedback is from our perspective a rare and valuable gift, particularly in today’s fast-paced and

competitive research environment. We have taken the comments seriously and believe they have meaningfully strengthened the revised manuscript.

Reviewer #3 (Remarks to the Author):

This work by Dr. Brown is a massive bioinformatics effort to tease out transcriptional differences between control and obese mice, and then to compare (differential) responsiveness to 1-h hyperglycaemia. The study is well-performed, and the paper has all the by now standard features of high-quality output from the Pers group. As such, both the spatial (ARC vs. DVC), cell-type-specific, and "function-predicting" comparisons that had been made will be useful to the scientific community. While I am most supportive of the paper be published, I am of the view that the authors must perform critical experiments to both justify the biological significance of their data, and to prevent others from "spin-doctoring" their findings.

Response: We thank Reviewer #3 for their positive remarks and thorough review. We have revised the manuscript as needed to clarify our conclusions and to avoid overinterpreting our findings. As noted above, our primary aim was to identify the transcriptional responses of defined hypothalamic and dorsal vagal complex cell populations to experimentally induced hyperglycemia, and to assess how these responses are altered by high-fat-diet feeding. Our intention is to provide a foundational dataset and new hypotheses for future mechanistic studies. We hope that the revised text now more clearly reflects the scope and focus of our work.

Major concerns:
1. Experimental design. The study has two inherent problems:
ad 1) the period of 4h fasting prior to hyperglycaemia. Back in 2004, Tamas Horvath's group published rapid synaptic rewiring upon fasting, and leptin exposure, i.e. the removal of GABA and glutamate afferents from Agrp/Pomc neurons and their temporary replacement with astrocyte end-feet, to allow for the plastic remodelling of excitatory/inhibitory synapse populations. The authors find plenty of axon guidance molecules, RhoGTPases, Ras etc. in astrocytes, and changes in GABA receptors. These all could suggest that the authors indeed observe (even if inadvertently) plastic remodelling in the ARC, perhaps even involving Gnrh-to-Agrp synapses. This is a testable hypothesis that minimally needs quantitative anatomy.

Response: We appreciate the reviewer’s insightful comment regarding the potential for fasting-induced synaptic remodeling in hypothalamic circuits. However, investigation into the this type of response would require repeating all of the studies and employing completely different methods, some of which are not immediately available to us (e.g., quantitative electron microscopy). Given our distinctly different study objectives, we believe that quantitative assessment of plasticity lies outside the scope of the present work, but we agree that future studies are warranted to address is interesting question.

We have clarified this in the revised manuscript, noting that the observed transcriptional changes in astrocytic and neuronal populations reflect molecular responses to glucose elevation, rather than direct evidence of synaptic remodeling. The manuscript now states this explicitly in the Results to avoid any ambiguity about the interpretation of these data. In the Results section on page 20 we write:

“Although not direct evidence of synaptic remodeling, these findings suggest a fundamental shift in how hypothalamic neurons respond to an acute elevation of blood glucose levels in HFD-fed mice.”

2. 1-h hyperglycaemia: this is a very short period of time. Transcriptional waves often need much longer periods to manifest, and most importantly, stabilise. How can the authors argue that most of the changes they see (beyond immediate early and glucose-sensing-related genes) have anything to do with brain adaptation (and not just stochastic). It would be most impressive to see a later (e.g., 4h or 24h) time-point to resolve if any change becomes stable (or a "susceptibility gene set" emerges supporting and linking the experimental and human GWAS datasets).

Response: We appreciate the reviewer's important point about temporal dynamics in transcriptional responses. The 1-hour hyperglycemic clamp represents an intentional experimental decision to capture the initial transcriptional wave following glucose elevation. This timepoint was selected in part based on studies by Dunn-Meynell et al. (J Neuroscience 2009) and Levin et al. (Diabetes 2004), which demonstrated that brain glucose levels equilibrate with plasma within 30-60 minutes during sustained hyperglycemia.

We agree that longer exposures would likely reveal distinct transcriptional programs. Our focus on the acute response was driven by the goal of identifying primary glucose-sensing mechanisms before the engagement of compensatory pathways. We have revised the manuscript to clarify these objectives and their limitations, explicitly stating that our conclusions pertain to early transcriptional events rather than stable adaptations. We acknowledge that examining later timepoints (4h, 24h) would provide valuable complementary insights into the stabilization of these responses – a promising direction for future investigation.

2. General physiology: There is not a single biological benchmarking attempt to seriously advance physiology/metabolism research. This is a major caveat. Minimally, transcriptional changes for astrocytes, oligodendrocytes or neurons shall be justified by in situ hybridisation/histochemistry (e.g., "reactive gliosis" is not equivalent to an increase in GFAP expression but also a change in astrocyte morphology and positioning). Why not testing tight junction coupling by antibodies? Why not testing Gnrh cell input to Agrp neurons? If the authors are correct, Gnrh input shall decrease, which would allow by disinhibition to increase Agrp AP spiking/output.

Response: We thank the reviewer for this important distinction. We agree that increased GFAP expression alone does not constitute reactive gliosis. Our assessment of GFAP protein in the arcuate nucleus was performed as a sanity check to confirm previously reported findings that in mice, HFD feeding induces early glial activation in the mediobasal hypothalamus. We have clarified this distinction in the revised manuscript and removed the phrase “a marker of reactive astrogliosis”, while also noting that comprehensive morphological characterization was beyond the scope of our transcriptomics-focused study.

Regarding the additional validation experiments suggested, we acknowledge their value for extending our transcriptomic findings. While in situ hybridization for key markers and evaluation of tight junction coupling would provide valuable spatial

context, and electrophysiological characterization of GHRH-AgRP connectivity could test functional hypotheses, these approaches require specialized tissue preparation and collaborative expertise that reflect a major departure from our single-nucleus isolation protocol.

Perhaps more importantly, our study was designed as an unbiased transcriptomic survey to identify novel cell types involved in glucose sensing, with the expectation that these findings would generate testable hypotheses for follow-up studies. Your insightful comments are precisely those that we hoped our findings would elicit and we anticipate that others will be motivated to pursue them. We have shared our data through publicly accessible repositories to facilitate such targeted investigations by our group and others in the field (GSE262411). In ongoing work, we are pursuing several of these validation approaches, including physiological characterization of glucose responses in identified neuronal populations.

3. Use of GO and other classifiers: The Authors use classifiers in a very confusing fashion. What is the difference between e.g., „neurotransmitter regulation“ vs. „synaptic regulation“? Why are these terms coming up, along with axon guidance molecules, in glial cells? Why are Slit2 and Robo classified differently although they are a ligand-receptor pair. A very prudent and didactical approach needs to be made to not just crudely sum pathways that could be very misleading for follow-up studies.

Response: We thank the reviewer for pointing out that the original wording in this section resembled GO-term enrichment results. To clarify, Slit2 and Robo2 genes are not classified differently. Instead, they are genes that distinguish ARC/VMH astrocytes from DVC astrocytes, as identified from the principal component analysis. Slit2 is highly expressed in ARC/VMH astrocytes and Robo2 is highly expressed in DVC astrocytes. We have removed the descriptions of the individual genes and revised this section to ensure this point is clearly communicated.

4. Interpretations: These are excessive in the results. Interpretations are starting points to experiments. The authors cannot argue for "later research" every time. Then, the work shall go to a more bioinformatics-minded journal. Please clear up the results such that it is to the point, and does not rely on excessive argumentation (which could equally be false) to the data.

Response: We agree with the reviewer that the Results section contained interpretive language that could be perceived as speculative. In the revised manuscript, we have carefully edited this section to ensure that interpretations are clearly separated from primary data presentation. Each conclusion is now directly supported by quantitative or statistical evidence derived from the transcriptomic analysis. Broader conceptual implications are restricted to the Discussion, where they are framed in the context of existing literature.

We also take the reviewers point about excessive reliance on “later research” and have endeavored to limit that type of statement. With this said, however, we re-emphasize the hypothesis-generating nature of the discovery-based approach to our study – our goal is to identify new possibilities that warrant future investigation, including those you have presented to us.

More minor:
Line 73: can one talk or imply 'inhibition' when looking at RNA-seq data.

Response: We agree that RNA-seq data measure transcriptional expression rather than direct electrophysiological inhibition. We have revised the text to specify “transcriptomic signatures consistent with neuronal inhibition (i.e., decreased IEG expression)” rather than simply “inhibition.”

Lines 109-110 and 141-142: sentences seems fragmented.

Response: Both sentences have been rewritten for grammatical completeness and clarity.

Line 183: word missing after Agrp

Response: Thank you for catching this typographical omission. The missing word (“neurons”) has been added.

Fig. 2: please split this into two figures to separate astrocytes and neurons. It would be helpful for Fig. 2f to have background „clouds“ to encircle GABA vs. glutamate clusters because the many genes does not help the classifier. Conversely, DVC neurons are all „GABA“ and „glutamate“ subtypes but not specified by gene sets. This could be/should be resolved by adding in genes and encircle the neurotransmitter phenotypes instead.

Response: We have divided the original Figure 2 into two panels: Figure 2 (Nonneuronal clusters) now and New Figure 3 (Neuronal). Because the neuronal clusters are based on the literature and on the use of GABA and Glu for the DVC cell populations, the DVC cell populations are kept consistent with the prior publication.

Lines 178-179: „four GABA cell populations“ Th/Slc6a3 are likely TIDA cells. Why would these be GABAergic and not dopaminergic? Do the authors refer to developmental origins?

Response: We have clarified that the Th/Slc6a3 cluster likely corresponds to tuberoinfundibular dopamine neurons which co-express dopaminergic and GABAergic markers. The Results section on page 10 now mentions this dual phenotype and specifies that the term “GABAergic” refers to their co-expression of Slc32a1, not an exclusion of dopaminergic identity:

“In the ARC/VMH of mice receiving intravenous saline, transcriptional responses to HFD-feeding were observed predominantly in three GABAergic cell populations (co-expressing Slc32a1), namely Agrp, Ghrh, and Trh/Cxcl12 neurons and one dopaminergic population, namely Th/Slc6a3 (Fig. 3b,c) […]”

Line 224: why have oligodendrocytes to do something with 'reduction of synaptic structural components'? This makes little sense even if GO ontology is used given that it is neuronal structures that should change. How can this be reconciled? 'Physiolgoical adaptation' needs to be addressed otherwise this section is meaningless.

Response: We agree, though we did not want to cherry-pick the GO term, because GO enrichment reflects the curated functional annotations of genes rather than the direct biological activities of the cell type in which the genes are expressed. Although oligodendrocytes do not form synapses, genes expressed by those cells are annotated to synaptic structure GO terms because they regulate neuronal function through non-cell-autonomous mechanism.

Line 250: the statement means the cells that responded most are NOT within the DVC. Is this correct? If so, should not these cells be excluded from the analysis anyway?

Response: We appreciate the reviewer's question. The glucose-responsive cells in question were retained in the dataset, but we now clearly state in the revised text that these nuclei lie outside the canonical DVC boundaries. Although they are not part of the DVC proper, we chose to keep them because researchers interested in glucose sensing within the broader caudal hindbrain may still find these transcriptional signatures valuable.

REVIEWER COMMENTS

We thank all reviewers for setting aside time to review our manuscript besides probably many other tasks and commitments.

Reviewer #2 (Remarks to the Author):

The authors meticulously responded to each of my comments, while incorporating additional analyses, updated figure layouts, and text edits, which greatly improved the interpretability of their findings. Whereas I previously recommended this manuscript for publication, I now strongly recommend this manuscript be published in its current form.

We thank the reviewer for their constructive comments throughout the review process. They have helped to substantially improve the manuscript.

Reviewer #3 (Remarks to the Author):

The reviewer thanks the Authors for returning this revision, and their extensive rebuttal. The paper certainly retains significant interest even if it being a "useful dataset" for follow-up by others is a major turn-off and undersells the study.

Unfortunately, the major issue independently spotted by all referees remains open, it being the lack of biological validation. This is most unexpected for experimentally-versed Authors with significant resources.

Whilst this reviewer would wish this paper be published here, it cannot be without validation. The neuroscience community has seen so many misleading studies: what about all the lost investment, potentially career-ending mishaps, if any of the authors' interpretations were incorrect yet followed up. Who is to blame then?

Beyond the "moral obligation", the Authors could have performed simple experiments: neuroanatomy by light microscopy (i.e., dual or triple labelling). In situ hybridisation by HCR or similar "out of the box" kit. The GHRH connectome could have been reconstructed using an AAV delivering GFP/mCherry and combined with a Fos or other gene from the DEG set.

This reviewer will not change their view even at a later stage: every bioinformatics study should be backed up by biology. Otherwise, the dataset could be made public on GEO with no strings attached for anyone to mine the data. At the same time, this reviewer would caution that it is customary to disclose reviews/rebuttals with published papers in full. Thus, Figure 1 could well become "published material" through that route (the data are fantastic yet collateral/dispensable to this particular study).

Lastly, and even if this had not been part of the original review (the discussion with Referee 2 was quite interesting): Arx is critical for the development, specification, and maintenance of pancreatic alpha cells. Thus, Arx-expressing neurons being foremost to glucose manipulation could be contextualised across tissues and organs.

We thank the reviewer for emphasizing the importance of biological validation and agree that experimental confirmation is generally essential to support bioinformatic inference. In direct response to this concern, we have now performed RNAscope fluorescent *in situ* hybridization experiments to orthogonally validate key findings from our single-nucleus RNA-sequencing analysis.

Specifically, we conducted RNAscope on coronal hypothalamic sections from wild-type male and female mice (n=3+3), focusing on the arcuate/ventromedial hypothalamic region. These experiments were designed to validate both *i)* the expression of glucose-sensing machinery (the *Gck* gene) in the *Ghrh* and Slc6a3 populations and *ii)* the cell-type specificity of differentially expressed genes (*Foxp2*) identified following experimentally induced hyperglycemia in *Ghrh* cells.

Our RNAscope analyses confirmed robust co-expression of the glucose-sensing enzyme *Gck* with *Ghrh* and *Slc6a3 in situ*. Quantification revealed that the majority of *Slc6a3*-positive cells (84.1%) and *Ghrh*-positive cells (88.2%) co-express *Gck*, providing direct anatomical validation that these glucose-responsive neuronal populations express canonical glucose-sensing machinery at the tissue level (new Supplementary Fig. 8). These data substantiate our transcriptomic inference that these populations express genes to directly respond to changes in glucose availability.
In addition, we experimentally validated the expression of *Foxp2*, one of the top differentially expressed genes identified in *Ghrh* neurons following hyperglycemia (control diet). RNAscope confirmed *Foxp2* puncta within *Ghrh*-positive neurons, with approximately 50.8% of *Ghrh*-expressing cells co-expressing *Foxp2* (new Supplementary Fig. 6). This supports the conclusion that *Foxp2* is genuinely expressed in this neuronal population *in vivo* and not an artifact of transcriptomic analysis.

Together, these RNAscope experiments provide independent and direct biological validation of our snRNA-seq findings. They confirm the anatomical presence and co-expression of key glucose-sensing and glucose-responsive genes in hypothalamic neuron populations, thereby addressing the reviewer's primary concern and strengthening the biological grounding of the study.

Key additions to the Results

Page 15:

> Among the differentially expressed genes identified in *Ghrh* neurons, *Foxp2* (Supplementary Fig. 6a), a marker of tuberal VMH neurons [34], was detected in approximately half of *Ghrh*-positive cells (50.8%) in wild-type mice by RNAscope (Supplementary Fig. 6b,c), confirming its expression in *Ghrh* neurons *in situ*.

Results p 18:

To determine whether glucose-responsive neuronal populations express core glucose-sensing machinery in situ, we performed RNAscope and found that the majority of both *Ghrh* and *Slc6a3*-positive neurons co-expressed *Gck* (Ghrh^{+}: 88.2%; Slc6a3^{+}: 84.1%; Supplementary Fig. 8b–d).

Supplemental Figures 6 and 8

Supplementary Figure 6: Co-expression of *Ghrh and Foxp2* in the ARC.

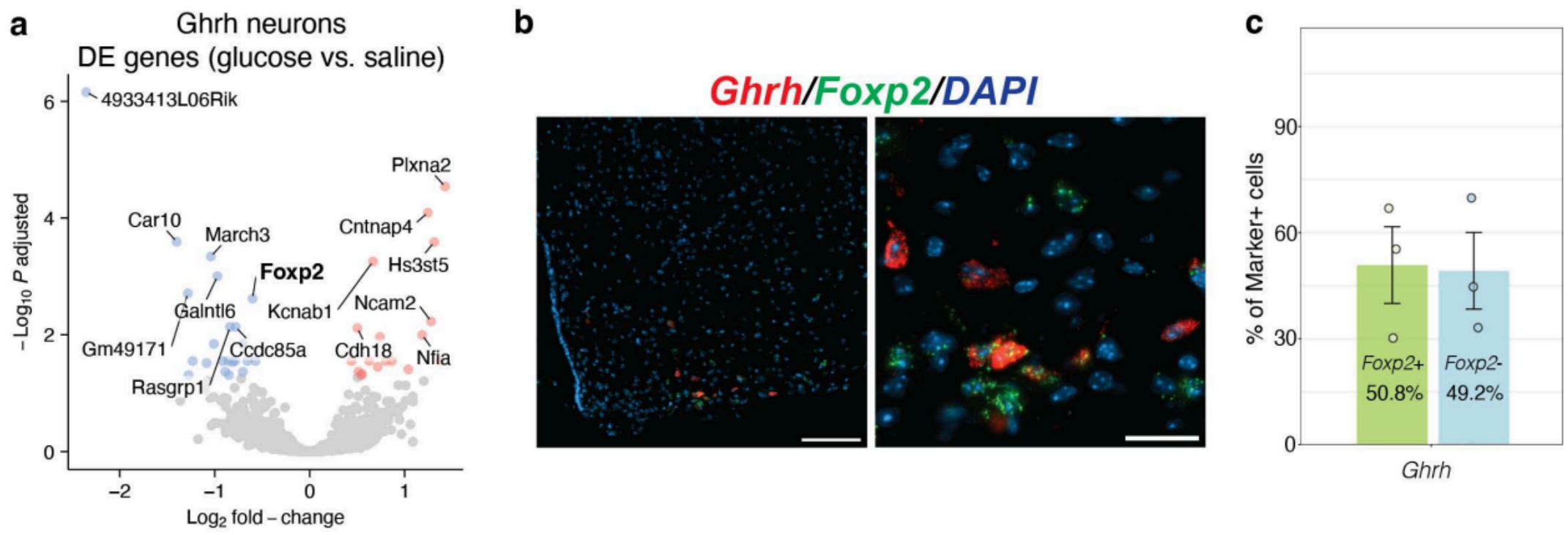

a, Volcano plot showing differentially expressed genes in Ghrh neurons from glucose *vs.* saline infusion in control diet-fed mice. Differentially expressed genes (FDR<0.05) are shown in red for genes unregulated by glucose and blue for genes down regulated by glucose. *Foxp2* is identified among glucose regulated transcripts. **b,** Representative RNAscope fluorescent in situ hybridization images from coronal hypothalamic sections of wild-type mixed-sex mice (n = 3), showing *Ghrh* (red), *Foxp2* (green), and DAPI (blue) expression in the ARC/VMH. Low- and high-magnification are shown. Scale bars: 100 µm (left), 25 µm (right) **c,** Quantification showing approximately half of *Ghrh*+ cells co-express *Foxp2 (Foxp2+:* 50.8%*; Foxp2*-:49.2%). Individual data points represent biological replicates; bars show mean ± s.e.m.

Supplementary Figure 8: Glucose sensing analysis and co-expression of *Gck*, *Slc6a3,* *and Ghrh* in the ARC.

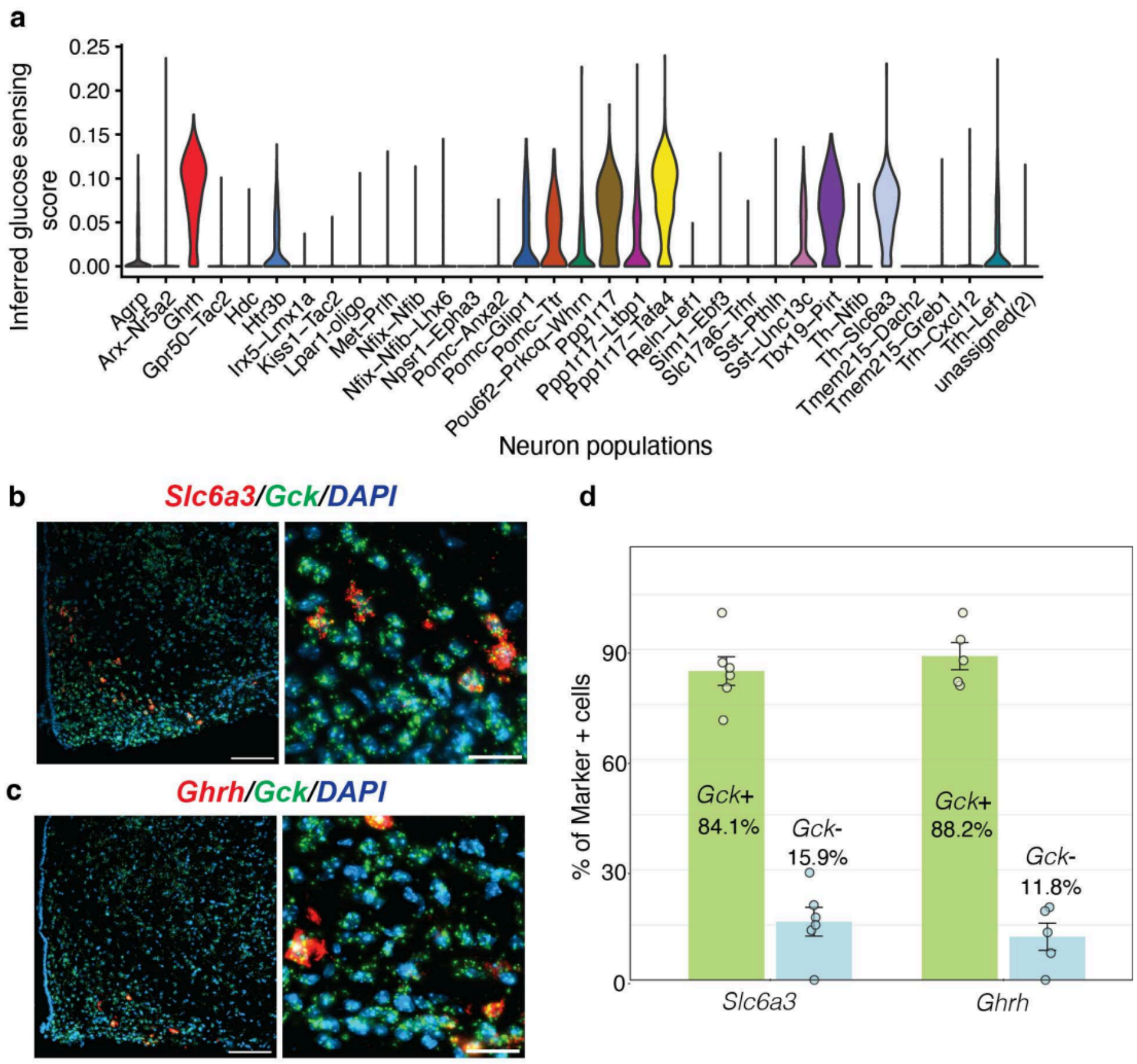

a, Glucose sensing score based on expression of key genes (Gck, Slc2a2, Abcc8, and Kcnj11) across ARC/VMH neuronal populations. **b,** Representative RNAscope fluorescent in situ hybridization images from coronal hypothalamic sections of wild-type mixed-sex mice (n = 5-6), showing *Slc6a3* (red), *Gck* (green), and DAPI (blue) **c,** *Ghrh* (red), *Gck* (green) and DAPI (blue) expression in the ARC/VMH. Low- and high-magnification are shown. Scale bars: 100 µm (left), 25 µm (right) **d,** Quantification showing percentage of *Slc6a3*+ cells co-express *Gck (Gck+:* 84.1%*; Gck-*:15.9%) and Ghrh+ cells co-express Gck (Gck+: 88.2%; Gck-:11.8%). Individual data points represent biological replicates; bars show mean ± s.e.m.

REVIEWER COMMENTS

Reviewer #3 (Remarks to the Author):

I appreciate the authors' renewed efforts to provide biological confirmation/support for some of their findings. While I am content with the experimentation itself, I suggest to better integrate the new neuroanatomy data and move them from the supporting figures to the main ones to increase visibility. I also think the authors shall highlight the minimal experimental follow-up as a limitation already in the abstract. I have no questions beyond this limited reorganisation.

Response to the Reviewer #3's comments:

We have updated the Abstract to accommodate that point, by changing the to: "These findings **point to [instead of 'reveal']** the brain's intrinsic capacity to sense hyperglycemia and **suggest [instead of 'demonstrate']** that this detection system is profoundly impaired in obesity."

Because we already have eight dense display items in the main text we have chosen to keep the validatory neuroanatomy data in the Supplement. We explicitly reference these data from the Results:

- "Among the differentially expressed genes identified in *Ghrh* neurons, *Foxp2* (Supplementary Fig. 6a), a marker of tuberal VMH neurons [34], was detected in approximately half of *Ghrh*-positive cells (50.8%) in wild-type mice by RNAscope (Supplementary Fig. 6b,c), confirming its expression in *Ghrh* neurons *in situ*."
- And: "To determine whether glucose-responsive neuronal populations express core glucose-sensing machinery *in situ*, we performed RNAscope and found that the majority of both *Ghrh* and *Slc6a3*-positive neurons co-expressed *Gck* ($Ghrh^+$: 88.2%; $Slc6a3^+$: 84.1%; Supplementary Fig. 8b–d)."
