# Supplementary material for: Single-cell profiling identifies obesity-disrupted brain glucose sensing
Source: Nat Commun. 2026 Aug 31;17:10342. doi: 10.1038/s41467-026-77116-9 (PMC13623956; doi:10.1038/s41467-026-77116-9)
Supplement: Supplementary file 5 [file 41467_2026_77116_MOESM3_ESM.pdf]

Description of Supplementary Data

Supplementary Data 1. Differential gene expression in ARC/VMH non-neuronal cell populations across experimental comparisons. Differential expression analysis was performed separately for each cell type using edgeR. Each worksheet contains genes with a nominal *P* value < 0.05 for one experimental comparison. Glucose_Response_Control_Diet compares glucose infusion with saline infusion in control diet-fed mice, Glucose_Response_HFD compares glucose infusion with saline infusion in high-fat diet-fed mice, and Diet_Response_Saline_Infusion compares high-fat diet-fed with control diet-fed mice following saline infusion. Tables report the log2 fold change (logFC), average log2 counts per million (logCPM), likelihood ratio statistic (LR), nominal *P* value, false discovery rate (FDR), gene symbol, and cell type.

Supplementary Data 2. Differential gene expression in DVC non-neuronal cell populations across experimental comparisons. Differential expression analysis was performed separately for each cell type using edgeR. Each worksheet contains genes with a nominal *P* value < 0.05 for one experimental comparison. Glucose_Response_Control_Diet compares glucose infusion with saline infusion in control diet-fed mice, Glucose_Response_HFD compares glucose infusion with saline infusion in high-fat diet-fed mice, and Diet_Response_Saline_Infusion compares high-fat diet-fed with control diet-fed mice following saline infusion. Tables report the log2 fold change (logFC), average log2 counts per million (logCPM), likelihood ratio statistic (LR), nominal *P* value, false discovery rate (FDR), gene symbol, and cell type.

Supplementary Data 3. Cell counts for cell populations in the ARC/VMH and DVC. The Cell Counts worksheet reports the total number of nuclei assigned to each cell population in each brain region following quality control and cell annotation. Columns indicate the celltype, brain_region, and total indicating the total number of nuclei included in downstream analyses.

Supplementary Data 4. Differential gene expression in ARC/VMH neuronal cell populations across experimental comparisons. Differential expression analysis was performed separately for each cell type using edgeR. Each worksheet contains genes with a nominal *P* value < 0.05 for one experimental comparison. Glucose_Response_Control_Diet compares glucose infusion with saline infusion in control diet-fed mice, Glucose_Response_HFD compares glucose infusion with saline infusion in high-fat diet-fed mice, and Diet_Response_Saline_Infusion compares high-fat diet-fed with control diet-fed mice following saline infusion. Tables report the log2 fold change (logFC), average log2 counts per million (logCPM), likelihood ratio statistic (LR), nominal *P* value, false discovery rate (FDR), gene symbol, and cell type.

Supplementary Data 5. Differential gene expression in DVC neuronal cell populations across experimental comparisons. Differential expression analysis was performed separately for each cell type using edgeR. Each worksheet contains genes with a nominal *P* value < 0.05 for one experimental comparison. Glucose_Response_Control_Diet compares glucose infusion with saline infusion in control diet-fed mice, Glucose_Response_HFD compares glucose infusion with saline infusion in high-fat diet-fed mice, and Diet_Response_Saline_Infusion compares high-fat diet-fed with control diet-fed mice following saline infusion. Tables report the log2 fold change (logFC), average log2 counts per

million (logCPM), likelihood ratio statistic (LR), nominal *P* value, false discovery rate (FDR), gene symbol, and cell type.

Supplementary Data 6. Functional enrichment analysis of differentially expressed genes. Functional enrichment analysis was performed separately for the FDR<0.05 differentially expressed genes from each cell population using the MetaBrain Network functional enrichment tool. The worksheet contains the enrichment results for the comparison of glucose infusion with saline infusion in control diet-fed mice. Tables report the Gene Ontology (GO) identifier, gene set name, nominal *P* value, the corresponding cell type, –log10(*P* value), and false discovery rate (FDR).

Supplementary Data 7. Cell type prioritization using CELLECT and MAGMA. CELLECT was used to integrate cell type-specific gene expression profiles with publicly available random glucose genome-wide association study (GWAS) summary statistics[43,44] to identify cell populations enriched for genetic associations with glycemic traits. The CELLECT_ldsc worksheet contains CELLECT-LDSC results, and the MAGMA_gene worksheet contains gene-level association results generated using CELLECT-gene including the GWAS dataset, specificity annotation, Ensembl gene ID, gene symbol, ESMU percentile, MAGMA gene percentile, ESMU score, and MAGMA gene association *P* value.
